# Comparative Effectiveness and Safety of Acupuncture-Related Therapies for Psoriasis Vulgaris: An Updated Systematic Review and Network Meta-Analysis

**DOI:** 10.3390/healthcare14213718

**Published:** 2026-11-02

**Authors:** Jiaqi Gao, Guotao Qiu, Sunyang Tan, Yuting Jin, Yuxin Liu, Kaitao Luo

**Affiliations:** 1 Graduate School, Zhejiang Chinese Medical University, Hangzhou 310053, China; 2 Department of Acupuncture and Moxibustion, Jiaxing Hospital of Traditional Chinese Medicine Affiliated with Zhejiang Chinese Medical University, Jiaxing 314001, China

**Keywords:** psoriasis vulgaris, acupuncture therapy, fire-needle acupuncture, moxibustion, network meta-analysis

## Abstract

**Highlights:**

**What are the main findings?**

• This network meta-analysis included 33 randomized controlled trials (2711 participants) and compared 15 clinically distinct acupuncture-related treatment nodes for psoriasis vulgaris.
• For PASI, MA + WM, MA + CHM, FN, and FN + CHM showed statistically significant favorable PASI estimates versus WM, while outcome-specific signals were also observed for total response rate, quality of life, pruritus, inflammatory markers, and adverse events.

**What are the implications of the main findings?**

• Separating acupuncture techniques and add-on regimens into distinct treatment nodes may improve the clinical interpretability of comparative evidence.
• Treatment rankings should be interpreted cautiously because of substantial heterogeneity, very low certainty of PASI evidence, and sensitivity to comparator-node definitions.

**Abstract:**

Background/Objectives: A previous network meta-analysis of acupuncture-related treatments for psoriasis was based on searches completed in 2016 and grouped several distinct techniques into broad treatment categories. Since then, substantial randomized evidence has accumulated. We therefore updated the evidence and compared the effectiveness and safety of distinct acupuncture-related therapies for psoriasis vulgaris. Methods: Nine Chinese and international databases were searched from inception to March 30, 2026 for randomized controlled trials. Risk of bias was assessed using RoB 2, and certainty of evidence for the primary outcome was evaluated with CINeMA. A frequentist network meta-analysis was performed, and treatment rankings were estimated using SUCRA. Results: Thirty-three RCTs involving 2711 participants were included. For post-treatment PASI, significant reductions compared with WM were observed for MA + WM (SMD −2.44, 95% CI −4.85 to −0.03), MA + CHM (−1.79, −3.19 to −0.39), FN (−1.77, −3.17 to −0.37), and FN + CHM (−1.74, −2.55 to −0.93); however, all corresponding 95% prediction intervals crossed the prespecified equivalence range, and CINeMA rated these comparisons as very low certainty. Statistically significant favorable estimates were also observed for total response rate with MA + WM, FN, FN + CHM, and MWA + CHM; for DLQI with FN, FN + CHM, MA + CHM, and MOX; and for Pruritus Score with MWA + CHM, FN, FN + CHM, and MA + CHM. Some regimens showed lower reported odds of adverse events than WM, although safety estimates were based on sparse events and several zero-event arms. Post hoc sensitivity analyses retained the direction of several key effects, although some rankings and the MA + WM comparison were sensitive to alternative node definitions. Conclusions: Several acupuncture-related monotherapies and add-on regimens showed favorable effects across clinically relevant outcomes, but no single treatment was consistently superior. Interpretation remains limited by heterogeneity, inconsistency in some secondary outcomes, and very low certainty of evidence for PASI.

## 1. Introduction

Psoriasis is a chronic, relapsing inflammatory disease shaped by genetic susceptibility, environmental factors, and immune dysfunction. Plaque psoriasis, the most common form, appears as well-defined red papules or plaques covered with silvery-white scales and may cause itching, pain, or burning [1]. The disease also affects health beyond the skin and is associated with psoriatic arthritis, nail disease, cardiovascular disease, metabolic syndrome, inflammatory bowel disease, depression, and anxiety [1,2,3].

At least 100 million people worldwide are affected by psoriasis, and reported prevalence across countries ranges from approximately 0.09% to 11.4% [3]. Recent multinational data further demonstrate substantial geographic variation in psoriasis prevalence [4]. Global Burden of Disease data show that the age-standardized incidence reached 62 cases per 100,000 population in 2021 and has continued to rise since 1990 [5]. Recurrent flares, persistent itching, and visible lesions can substantially reduce quality of life and create major psychological, social, and financial burdens [1].

The biological mechanisms of psoriasis are not fully understood, but abnormal activation of dendritic cells and the IL-23/IL-17 pathway appears to play a central role [6]. Activated dendritic cells release tumor necrosis factor-α (TNF-α) and IL-23, which promote T-helper 17-cell development and the release of IL-17 and IL-22. These inflammatory signals stimulate excessive growth of skin cells and further immune activity, creating a cycle that sustains inflammation [6,7].

Other pathways may also contribute to the disease. Substance P and its receptors may promote nerve-related inflammation, itching, and persistent skin lesions [8]. Bidirectional communication between cutaneous nerves and immune cells may contribute to inflammation and pruritus and can be modulated by environmental and psychological stressors [9]. Cardiometabolic comorbidities, including obesity, insulin resistance, and dyslipidemia, share inflammatory pathways with psoriasis, particularly TNF-α and the IL-23/IL-17 axis [10].

Treatment options include topical medicines, phototherapy, conventional systemic drugs, targeted small-molecule therapies, and biologic agents. Treatment is selected according to the extent and severity of the lesions, involvement of sensitive body sites, and coexisting conditions [2]. Long-term topical corticosteroid use may cause local adverse effects such as skin atrophy and telangiectasia [11]. Conventional systemic therapies such as methotrexate, ciclosporin, and acitretin require treatment-specific safety monitoring because of potentially serious toxicities [12]. Biologic therapies have substantially improved skin clearance in moderate-to-severe psoriasis [13]. Targeted small-molecule agents have expanded systemic treatment options, although long-term real-world evidence remains comparatively limited for some newer agents [14,15]. Biologic therapies also require treatment-specific safety monitoring, including attention to infection risk [16,17]. Safer supportive treatments that can be used as part of long-term care are therefore needed.

Acupuncture is widely used in traditional Chinese medicine for psoriasis. Because cutaneous trauma can trigger new psoriatic lesions through the Koebner phenomenon, needling-related skin injury is also clinically relevant [18].

A psoriasis flare following acupuncture has been reported, although the frequency and clinical significance of this event remain uncertain [19]. Current approaches include manual acupuncture, electroacupuncture, fire-needle acupuncture, moxibustion, catgut embedding, and microwave acupuncture. Systematic reviews suggest that acupuncture-related therapies may improve psoriasis outcomes, including PASI-related measures, although the evidence remains limited by small trials, clinical heterogeneity, and methodological weaknesses [20,21]. Fire-needle acupuncture has been associated with improvements in plaque psoriasis and higher PASI response rates, while previous meta-analytic evidence suggests that fire-needle-based treatment may also reduce recurrence [22,23].

Moxibustion has been associated with improvements in PASI and quality of life in clinical studies and with modulation of inflammatory responses in experimental models [24,25,26]. Experimental studies suggest that acupuncture-related stimulation may modulate T-cell responses, inflammatory cytokines, and substance P signaling [8,27,28].

The comparative evidence currently available is outdated and insufficiently technique-specific. Yeh et al. published a network meta-analysis in 2017 based on literature searches completed on 15 March 2016 [29]. That review included only 13 randomized controlled trials, of which 10 contributed to the network analysis, and grouped several clinically distinct interventions into broad categories such as “acupuncture,” acupressure, bloodletting, catgut embedding, drug treatment, and narrowband ultraviolet B phototherapy. Consequently, it could not determine whether individual acupuncture techniques differed meaningfully in their clinical effects. Since 2016, a substantial number of randomized trials evaluating fire-needle acupuncture, manual acupuncture, moxibustion, microwave acupuncture, and combined acupuncture techniques have been published. An updated synthesis using contemporary risk-of-bias and certainty-of-evidence methods is therefore warranted.

Accordingly, we conducted an updated systematic review and network meta-analysis of randomized controlled trials to compare clinically distinct acupuncture-related therapies for psoriasis vulgaris. Unlike the previous network meta-analysis, we analysed manual acupuncture, fire-needle acupuncture, moxibustion, microwave acupuncture, and their clinically relevant combinations as separate treatment nodes, while excluding interventions based on substantially different therapeutic principles, such as bloodletting, catgut embedding, and acupoint injection. The primary outcome was post-treatment Psoriasis Area and Severity Index (PASI), with total response rate, Dermatology Life Quality Index (DLQI), pruritus, inflammatory biomarkers, vascular endothelial growth factor, and adverse events evaluated as secondary outcomes. Risk of bias was assessed using RoB 2, and certainty of evidence for the primary outcome was evaluated using CINeMA (Confidence in Network Meta-Analysis; Institute of Social and Preventive Medicine, University of Bern, Bern, Switzerland). By incorporating evidence published through March 2026, this study aimed to provide a more current, technique-specific, and clinically interpretable comparison of acupuncture-related therapies for psoriasis.

## 2. Materials and Methods

The protocol for this systematic review and network meta-analysis was registered with PROSPERO (CRD420261435406). The review was conducted in accordance with the PRISMA 2020 statement and the PRISMA extension for network meta-analysis (PRISMA-NMA) [30,31]. The completed PRISMA-NMA checklist is provided in Supplementary Section S12.

### 2.1. Eligibility Criteria

#### 2.1.1. Study Design

Randomized controlled trials published in Chinese or English were eligible if they evaluated acupuncture-related treatment for psoriasis vulgaris. Because blinding and allocation concealment are difficult to implement in acupuncture trials, studies were not excluded solely for not using these methods.

#### 2.1.2. Participants

Participants were required to have a clear diagnosis of psoriasis vulgaris. No restrictions were placed on sex, ethnicity, age, or disease duration.

#### 2.1.3. Interventions and Comparators

Eligible interventions included acupuncture monotherapy, combinations of acupuncture techniques, and acupuncture-based add-on regimens involving Chinese herbal medicine (CHM), Western medicine (WM), or ultraviolet radiation (UR). Eligible comparators included WM, UR, or manual acupuncture (MA) when used as an active acupuncture comparator.

Treatment nodes were defined according to the components actually administered in each study. Add-on regimens were therefore retained as combination nodes rather than being classified as acupuncture monotherapy. For example, fire-needle acupuncture plus Western medicine versus the same Western medicine was coded as FN + WM versus WM, and fire-needle acupuncture plus ultraviolet therapy versus ultraviolet therapy alone was coded as FN + UR versus UR.

The primary network comprised 15 nodes: fire-needle acupuncture (FN), manual acupuncture (MA), moxibustion (MOX), Western medicine (WM), ultraviolet radiation (UR), FN + CHM, FN + MA, FN + UR, FN + WM, MA + CHM, MA + MOX, MA + UR, MA + WM, MOX + UR, and microwave acupuncture plus CHM (MWA + CHM). Accordingly, each node represented the complete clinical regimen actually administered rather than the acupuncture component alone.

For the primary analysis, different forms of Western medicine were grouped within the WM node, including topical, systemic, and combined topical and systemic treatments. Similarly, narrowband ultraviolet B (NB-UVB) phototherapy and 308-nm excimer therapy were grouped within the UR node. These broader definitions were examined in a post hoc sensitivity analysis using more specific comparator nodes. The broad WM node was used in the primary analysis as a pragmatic modelling choice to preserve network connectivity and to avoid creating multiple sparsely informed comparator nodes. This grouping was not intended to imply clinical equivalence among topical, systemic, and combined topical/systemic Western-medicine regimens; its influence was therefore examined in the post hoc sensitivity analysis using more specific comparator nodes.

Acupoint selection, needling technique, treatment frequency, and needle-retention time were not used as eligibility restrictions.

#### 2.1.4. Outcomes

The primary outcome was the PASI score measured at the end of treatment. Lower scores indicate less severe psoriasis.

Secondary outcomes included total response rate, Dermatology Life Quality Index (DLQI), Pruritus Score, serum tumor necrosis factor-α (TNF-α), vascular endothelial growth factor (VEGF), and adverse events. Total response rate was analyzed using the dichotomous clinical response definition reported in each trial rather than imposing a uniform PASI-response threshold. Most studies defined response using PASI improvement thresholds of ≥60% or ≥70%; however, some trials used different criteria, including overall score improvement ≥55% [32], TCM syndrome score reduction ≥65% [33], and skin-lesion clearance ≥50% [34]. The original definitions and responder counts used in the analysis are therefore provided in Supplementary Table S2.4, and the pooled outcome should be interpreted as an author-defined total response rate rather than a uniform PASI-response outcome. Pruritus was assessed using quantitative pruritus measures, including visual analogue scales or numerical rating scales.

### 2.2. Exclusion Criteria

The report was not a randomized controlled trial or did not provide clinically relevant evidence, such as a case report, review, theoretical paper, or animal study.

Studies were excluded if they evaluated acupuncture-related techniques outside the prespecified intervention framework, such as catgut embedding, acupoint injection, cutting therapy, bloodletting, scraping, intradermal needles, or plum-blossom needling.

The report duplicated another publication or described the same study population.

Outcome data were incomplete, treatment response was poorly defined, and the missing information could not be obtained from the study authors.

### 2.3. Search Strategy

Nine Chinese- and English-language databases were searched from inception to 30 March 2026, for randomized controlled trials of acupuncture-related treatments for psoriasis. The English-language databases were PubMed, the Cochrane Library, Web of Science, Embase, and Scopus. The Chinese databases were China National Knowledge Infrastructure, Wanfang Data, VIP Database, and the Chinese Biomedical Literature Database.

Two reviewers conducted the searches independently. Search terms were developed using the PICOS framework and combined database-specific subject headings with free-text terms. Disagreements were resolved by discussion with a third reviewer. Full search strategies for all databases are provided in Supplementary Section S1.

### 2.4. Study Selection and Data Extraction

Two reviewers independently screened the records against the eligibility criteria. After duplicate records were removed in EndNote (EedNote version 2025, Clarivate, Philadelphia, PA, USA) and checked manually, titles and abstracts were screened, followed by full-text review of potentially relevant reports.

At full-text review, eligibility was confirmed from the study population, treatments, comparators, and outcomes. Reports with missing data were excluded when the information could not be obtained from the authors. When several reports described the same participants, the report with the most complete data and strongest methods was retained. Disagreements were resolved by discussion or by a third reviewer.

The reviewers extracted publication details, sample size, randomisation and blinding methods, participant age and disease duration, acupuncture technique, accompanying drug treatment, treatment duration, PASI, total response rate, DLQI, TNF-α, VEGF, and adverse events. All data were cross-checked before analysis.

### 2.5. Risk of Bias and Certainty of Evidence

Two reviewers independently assessed risk of bias using the revised Cochrane Risk of Bias tool for randomized trials (RoB 2). The assessment covered five domains: bias arising from the randomization process, bias due to deviations from intended interventions, bias due to missing outcome data, bias in measurement of the outcome, and bias in selection of the reported result. Domain-level and overall judgments were assigned as low risk, some concerns, or high risk in accordance with the RoB 2 signaling questions and judgment algorithms. Disagreements were resolved by discussion or consultation with a third reviewer.

For outcome measurement, particular attention was given to whether knowledge of the assigned intervention could have influenced investigator-assessed or participant-reported outcomes. Open-label studies using subjective outcomes were not automatically classified as low risk in this domain unless the available information supported such a judgment.

The certainty of network evidence was assessed for the primary outcome, PASI, using CINeMA. The six CINeMA domains were within-study bias, reporting bias, indirectness, imprecision, heterogeneity, and incoherence. For imprecision and heterogeneity, an SMD range of −0.10 to 0.10 was prespecified as the clinical equivalence range. Indirectness was assessed by examining the distribution of potential effect modifiers across treatment nodes. Incoherence was evaluated using SIDE analyses where both direct and indirect evidence were available, together with the global design-by-treatment interaction test. Overall confidence ratings were generated using the CINeMA framework.

### 2.6. Statistical Analysis

Network meta-analyses were conducted in a frequentist framework using the mvmeta command within the Stata network suite (Stata version 19.5; StataCorp LLC, College Station, TX, USA) [35].

Continuous outcomes were reported as mean differences (MDs) or standardized mean differences (SMDs), as appropriate. Binary outcomes were summarized using relative risks (RRs) or odds ratios (ORs), as appropriate. Total response rate was analyzed using RRs because the event represented a desirable clinical response, whereas adverse events were analyzed using ORs because these events were relatively sparse. Zero-event cells were retained using the default 0.5 continuity correction implemented in the Stata network suite. Given the sparse events and variation in adverse-event definitions, SUCRA-based safety rankings were not emphasized.

All estimates are presented with 95% confidence intervals (CIs). For PASI, 95% prediction intervals (PrIs) were additionally calculated, and an SMD range of −0.10 to 0.10 was prespecified as the equivalence range.

Random-effects models were used throughout, with between-study heterogeneity estimated using restricted maximum likelihood. Network heterogeneity was summarized using τ^2^ and I^2^ and was reported numerically without applying categorical thresholds.

Global inconsistency was assessed using the design-by-treatment interaction test. For comparisons with both direct and indirect evidence, local inconsistency was assessed using node-splitting analyses. When a network contained no closed loops, local inconsistency was considered not estimable. Network plots showed the available treatment comparisons, and surface under the cumulative ranking curve (SUCRA) values were used to describe treatment rankings. Funnel plots were used to examine small-study effects and possible publication bias.

The transitivity assumption was assessed before network synthesis by comparing the distribution of potential clinical and methodological effect modifiers across treatment nodes. These included participant age, disease duration, clinical stage, treatment duration, and key characteristics of the acupuncture protocol. Acupuncture protocols were reviewed descriptively because acupoint selection, treatment frequency, and needling procedures were not consistently reported in a form suitable for quantitative comparison. Heterogeneity in comparator treatment was further examined in the post hoc sensitivity analysis that separated WM and UR into more specific treatment categories (Supplementary Section S11).

Two post hoc sensitivity analyses were conducted for PASI.

First, the influence of comparator-node definitions was examined by subdividing the broad WM and UR nodes used in the primary analysis. WM was reclassified as topical Western medicine (WM-T), systemic Western medicine (WM-S), or combined topical and systemic Western medicine (WM-TS), while UR was separated into NB-UVB and excimer therapy. After this subdivision, the two studies involving excimer therapy formed a disconnected component and were therefore excluded from the connected sensitivity network. The analysis was otherwise repeated using the same random-effects model and SMD effect measure as in the primary analysis (Supplementary Section S11.1).

Second, an acupuncture-focused sensitivity analysis was conducted to evaluate the findings in a network restricted to acupuncture monotherapy, combinations of acupuncture techniques, and acupuncture add-on regimens with a non-acupuncture comparator (WM or UR). Fourteen studies involving CHM-containing interventions were excluded. Lv Shiqi 2019 (FN + MA vs MA) was also excluded because the comparator was an active acupuncture intervention. The same random-effects model and SMD effect measure were used (Supplementary Section S11.2).

## 3. Results

### 3.1. Study Selection

**Figure 1:**
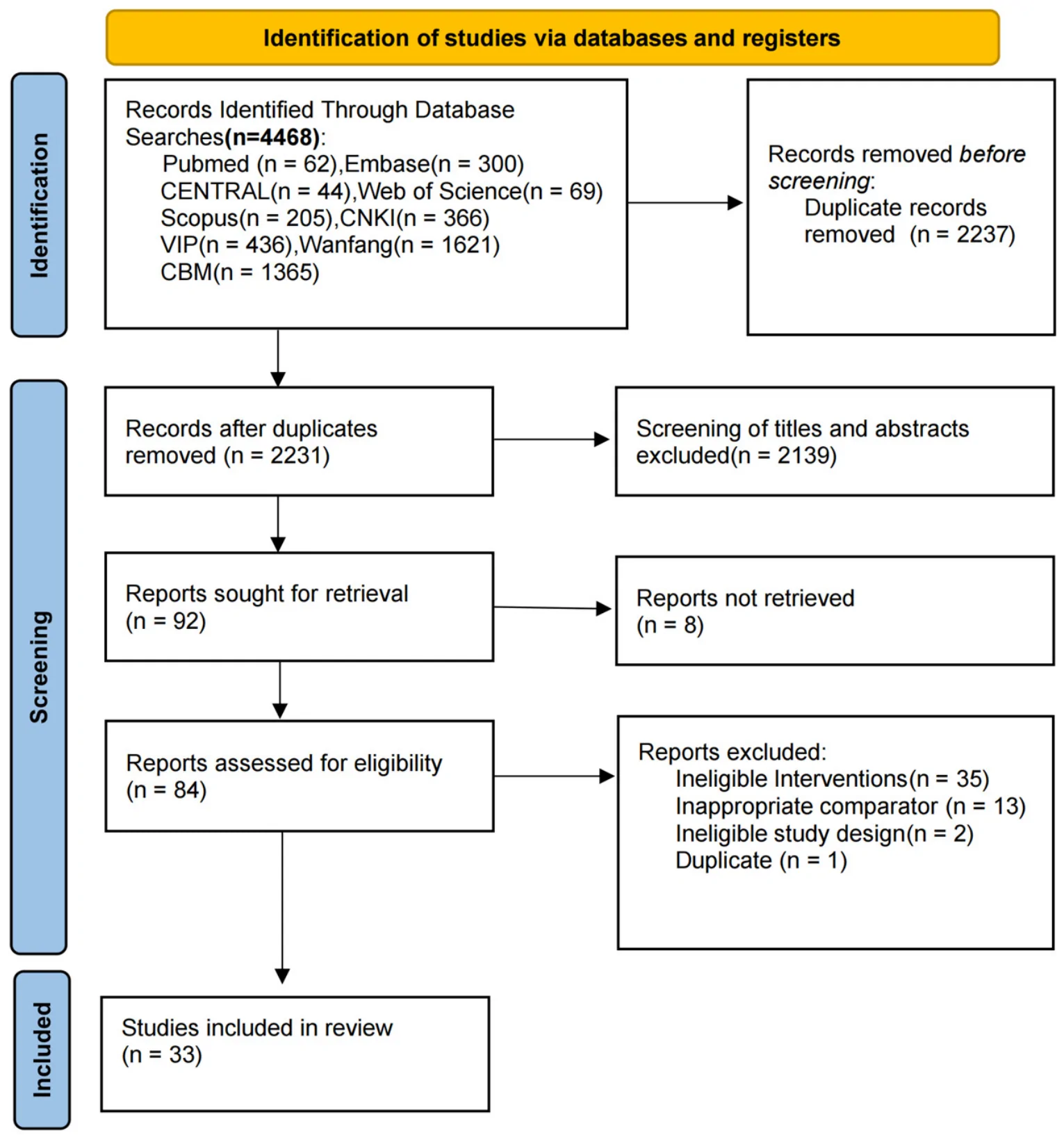
PRISMA 2020 flow diagram of study selection.

A total of 4468 records were identified through database searches, and 2237 duplicates were removed. Of the remaining 2231 records, 2139 were excluded after title and abstract screening. Ninety-two reports were sought for retrieval, of which eight could not be obtained, leaving 84 reports for full-text assessment. Fifty-one reports were excluded because of ineligible interventions (*n* = 35), inappropriate comparators (*n* = 13), ineligible study designs (*n* = 2), or duplication (*n* = 1). Ultimately, 33 reports representing 33 studies were included in the review (Figure 1).

### 3.2. Study Characteristics

All 33 included RCTs were conducted in China and involved a total of 2711 participants [32,33,34,36,37,38,39,40,41,42,43,44,45,46,47,48,49,50,51,52,53,54,55,56,57,58,59,60,61,62,63,64,65]. The trials were published between 2011 and 2025, with individual sample sizes ranging from 39 to 133 participants. All studies enrolled patients with stable-stage psoriasis vulgaris/plaque psoriasis, although some further specified traditional Chinese medicine syndrome subtypes or refractory disease. Reported group-level mean ages ranged from 27.84 to 53.02 years, and mean disease duration ranged from 0.47 to 15.80 years. Full study characteristics are provided in Table 1.

Assessment of potential effect modifiers showed broadly overlapping mean ages across treatment nodes (35.58–43.25 years), whereas mean disease duration (2.69–14.20 years) and treatment duration (3.00–12.00 weeks) varied more substantially (Supplementary Section S9, Table S9.1). Variation in acupuncture protocols and comparator definitions was considered when assessing the transitivity assumption, with comparator heterogeneity further examined in the post hoc sensitivity analyses.

Across the included studies, 15 intervention nodes were represented: FN, MA, MOX, WM, UR, FN + CHM, FN + MA, FN + UR, FN + WM, MA + CHM, MA + MOX, MA + UR, MA + WM, MOX + UR, and MWA + CHM. WM was the most common comparator (24 studies), followed by UR (8 studies) and MA (1 study). Treatment duration ranged from 3 to 12 weeks, and reported treatment frequency ranged from once daily to once every 7 days; seven studies did not report treatment frequency. Concomitant therapies included phototherapy, Western medication, and Chinese herbal medicine. Drug or procedural doses and separate post-treatment follow-up periods could not be consistently determined.

### 3.3. Risk of Bias

**Figure 2:**
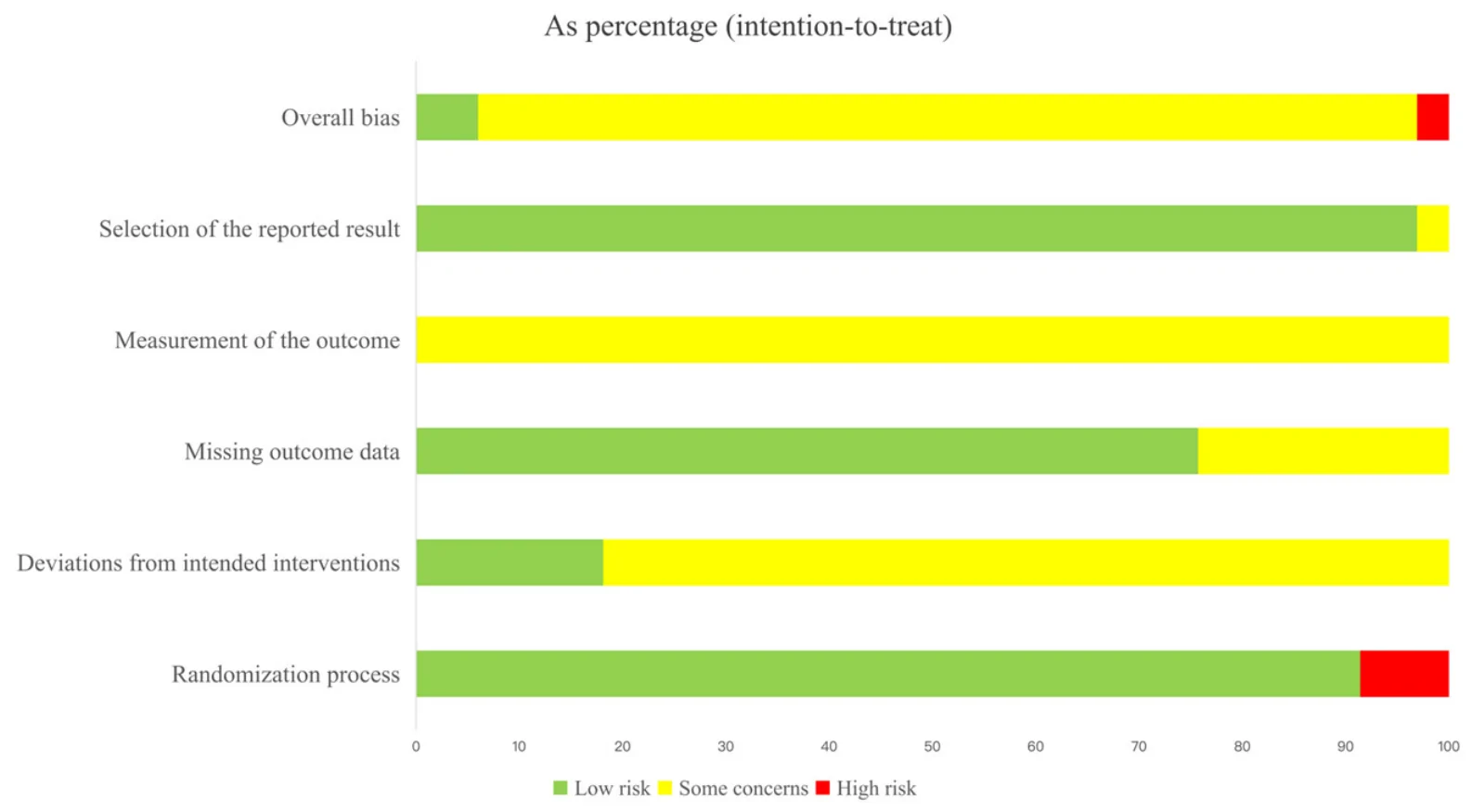
Risk-of-bias assessment of the included randomized controlled trials using RoB 2. Green indicates low risk, yellow indicates some concerns, and red indicates high risk.

The main risk-of-bias concerns involved deviations from intended interventions and measurement of the outcome. Deviations from intended interventions were rated as having some concerns in 27 of 33 studies (81.8%), whereas 6 studies (18.2%) were rated as low risk in this domain. Because the included acupuncture trials were generally open-label and adequate blinding of outcome assessors was not reported, measurement of the outcome was rated as having some concerns in all 33 studies (100%). Missing outcome data raised some concerns in 8 studies (24.2%), while the remaining 25 studies (75.8%) were rated as low risk. For the randomization process, 32 studies (97.0%) were rated as low risk and 1 study (3.0%) as high risk. Selection of the reported result was rated as low risk in 32 studies (97.0%) and as having some concerns in 1 study (3.0%). Overall, 30 studies (90.9%) were rated as having some concerns, 2 studies (6.1%) as low risk, and 1 study (3.0%) as high risk (Figure 2; Supplementary Section S4, Table S4.1).

### 3.4. Network Heterogeneity and Inconsistency

For PASI, heterogeneity was τ^2^ = 1.46 (I^2^ = 93.83%). The global design-by-treatment interaction test showed no statistical evidence of inconsistency (χ^2^ = 0.38, *p* = 0.5381). The network was predominantly star-shaped, with only one closed loop involving FN + MA, MA, and WM. Accordingly, meaningful local incoherence assessment was restricted to the three comparisons within this loop (FN + MA vs MA, FN + MA vs WM, and MA vs WM), none of which showed statistical evidence of inconsistency (all *p* = 0.538). For the remaining comparisons, independent indirect evidence was not estimable (Supplementary Section S5, Tables S5.1 and S5.2).

For DLQI, τ^2^ was 0.14 (I^2^ = 61.87%) and the global inconsistency test yielded χ^2^ = 4.77 (*p* = 0.0589). For Pruritus Score, τ^2^ was 0.28 (I^2^ = 81.07%), with evidence of global inconsistency (χ^2^ = 58.64, *p* < 0.001). For TNF-α, τ^2^ was 0.00 (I^2^ = 0%), with evidence of global inconsistency (χ^2^ = 17.87, *p* < 0.001). For VEGF, τ^2^ was 0.17 (I^2^ = 76.14%) and the global test yielded χ^2^ = 4.88 (*p* = 0.1271). Local inconsistency could not be assessed for these four outcomes because their networks contained no closed loops. For adverse events, τ^2^ was 0.00 (I^2^ = 0%); the global test yielded χ^2^ = 0.14 (*p* = 0.7056), and none of the 11 estimable node-splitting comparisons showed evidence of local inconsistency (all *p* ≥ 0.923) (Supplementary Section S5, Tables S5.1 and S5.4).

### 3.5. Primary Outcome: PASI

**Figure 3:**
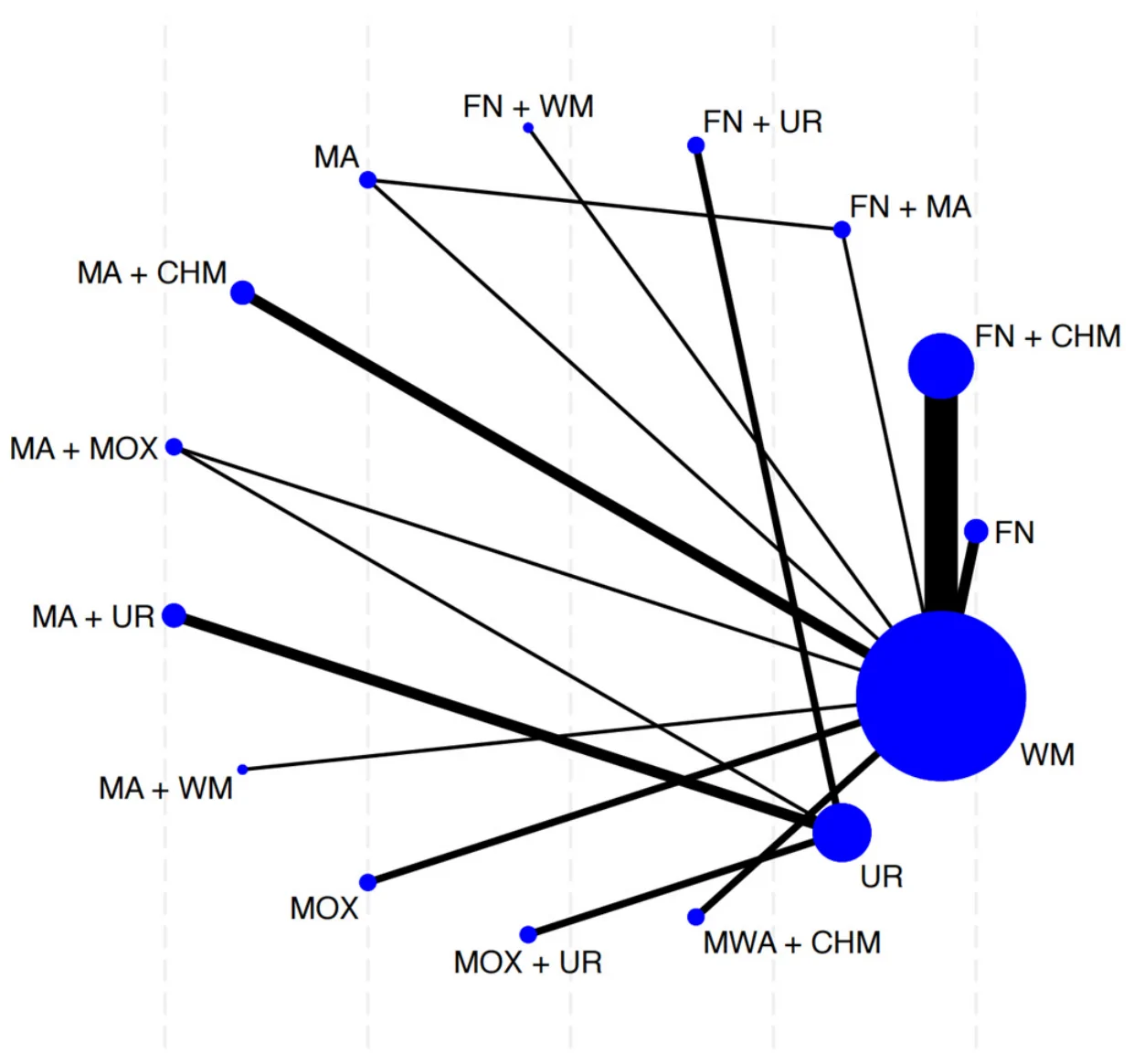
Network plot of post-treatment PASI. Node size is proportional to the total number of participants assigned to each intervention, and line thickness is proportional to the amount of direct comparative evidence.

**Figure 4:**
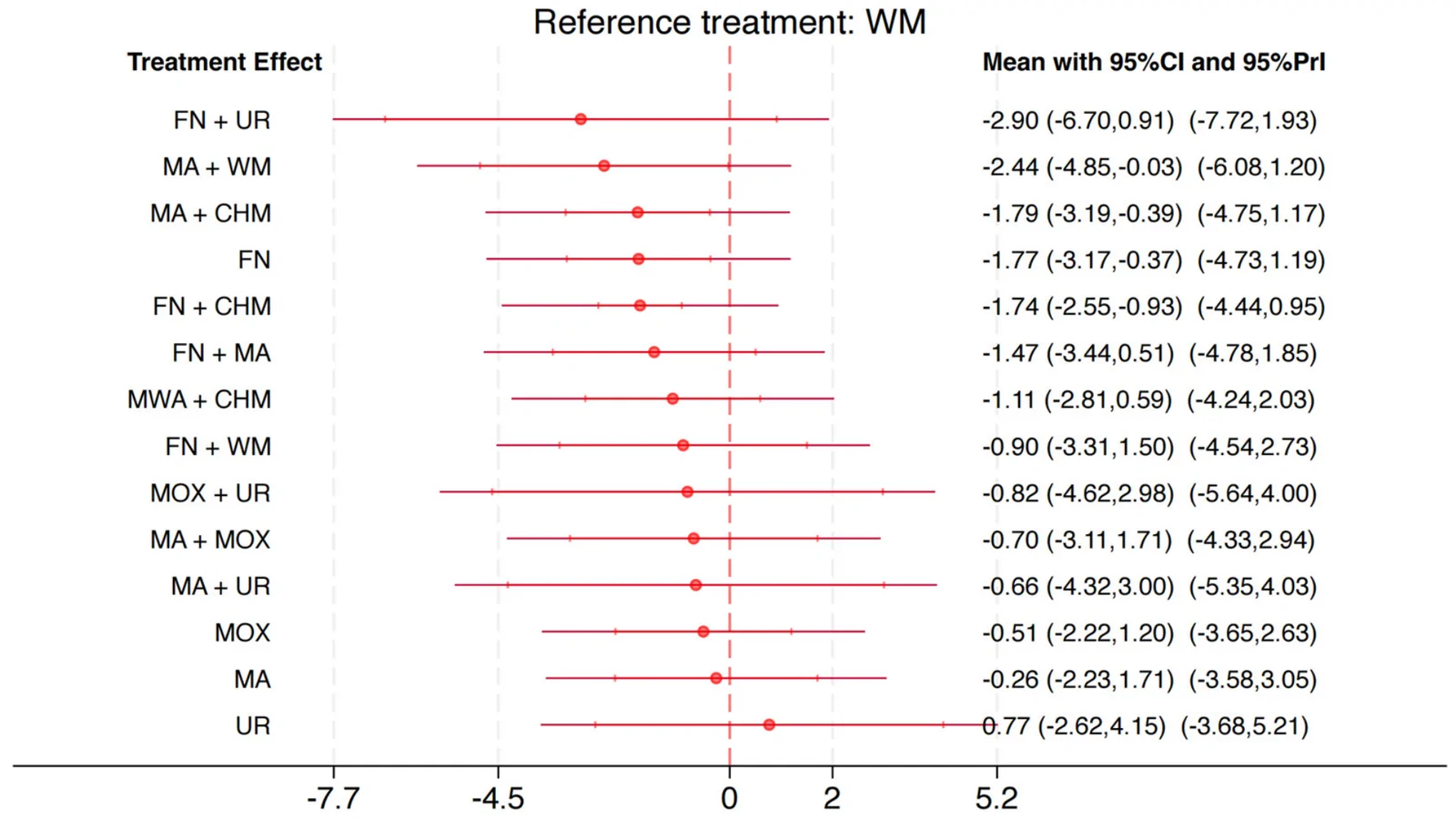
Effect estimates are standardized mean differences (SMDs) with 95% confidence intervals (CIs) and 95% prediction intervals (PrIs); negative values favor the acupuncture-related intervention.

The post-treatment PASI network included 33 studies, 2707 participants, and 15 intervention nodes, with WM as the reference treatment (Figure 3 and Figure 4). The difference from the overall sample of 2711 participants resulted from four dropouts: three in Hu 2018 [50] and one in Jie 2015 [38]. Compared with WM, PASI was significantly lower with MA + WM (SMD −2.44, 95% CI −4.85 to −0.03; 95% PrI −6.08 to 1.20), MA + CHM (SMD −1.79, 95% CI −3.19 to −0.39; 95% PrI −4.75 to 1.17), FN (SMD −1.77, 95% CI −3.17 to −0.37; 95% PrI −4.73 to 1.19), and FN + CHM (SMD −1.74, 95% CI −2.55 to −0.93; 95% PrI −4.44 to 0.95). However, all corresponding 95% prediction intervals crossed the prespecified equivalence range of −0.10 to 0.10.

FN + UR had the highest SUCRA value (83.4%), followed by MA + WM (77.7%) and FN (68.7%). The estimate for FN + UR versus WM was not statistically significant (SMD −2.90, 95% CI −6.70 to 0.91; 95% PrI −7.72 to 1.93) (Figure S7.1; Table S7.1). CINeMA rated the evidence for the significant comparisons of MA + WM, MA + CHM, FN, and FN + CHM versus WM as very low certainty (Supplementary Section S9, Table S9.2). The league table also showed significant differences favoring FN + UR over UR (SMD −3.66, 95% CI −5.42 to −1.91) and MA + UR over UR (SMD −1.43, 95% CI −2.82 to −0.03) (Supplementary Section S8, Table S8.1).

### 3.6. Secondary Outcomes and Safety

#### 3.6.1. Total Response Rate

The total response rate network included 31 studies, 2541 participants, and 15 intervention nodes, with WM as the reference treatment (Figure S6.1). Compared with WM, response rates were significantly higher with MA + WM (RR 1.71, 95% CI 1.04–2.82), FN (RR 1.68, 95% CI 1.27–2.22), FN + CHM (RR 1.56, 95% CI 1.35–1.81), and MWA + CHM (RR 1.36, 95% CI 1.02–1.81). The estimate for FN + MA was no longer statistically significant (RR 1.47, 95% CI 0.97–2.23). All other comparisons with WM also included the null value, including MOX + UR (RR 7.03, 95% CI 0.37–133.80), despite having the highest SUCRA value (87.7%). FN (75.5%) and MA + WM (74.0%) ranked second and third, respectively (Figure S7.2; Table S7.2). The league table further showed higher response rates for FN (RR 2.15, 95% CI 1.05–4.37), FN + CHM (RR 2.00, 95% CI 1.02–3.91), FN + UR (RR 1.38, 95% CI 1.05–1.81), and MA + MOX (RR 1.68, 95% CI 1.11–2.55) compared with UR (Supplementary Section S8, Table S8.2).

#### 3.6.2. Dermatology Life Quality Index

The DLQI network included 8 studies, 634 participants, and six intervention nodes, with WM as the reference treatment (Figure S6.2). Compared with WM, DLQI scores were significantly lower with FN (MD −4.26, 95% CI −5.54 to −2.97), FN + CHM (MD −3.47, 95% CI −4.84 to −2.10), MA + CHM (MD −3.26, 95% CI −4.99 to −1.53), and MOX (MD −2.83, 95% CI −4.90 to −0.76). The estimate for MA included no difference (MD −2.13, 95% CI −4.60 to 0.33). MA + CHM ranked first by SUCRA (94.9%), followed by FN + CHM (72.7%) and FN (63.6%) (Figure S7.3; Table S7.3). The league table showed no additional statistically significant differences among the active interventions (Supplementary Section S8, Table S8.3).

#### 3.6.3. Pruritus Score

The Pruritus Score network included 9 studies, 731 participants, and five intervention nodes, with WM as the reference treatment (Figure S6.3). All four active interventions significantly reduced Pruritus Scores compared with WM: MWA + CHM (SMD −3.35, 95% CI −4.21 to −2.49), FN (SMD −1.25, 95% CI −2.40 to −0.11), FN + CHM (SMD −1.25, 95% CI −1.83 to −0.66), and MA + CHM (SMD −1.19, 95% CI −2.01 to −0.37). MWA + CHM ranked first by SUCRA (99.9%), followed by FN + CHM (50.8%), FN (50.2%), and MA + CHM (48.6%) (Figure S7.4; Table S7.4). Formal league-table comparisons favored MWA + CHM over FN (SMD 2.10, 95% CI 0.67 to 3.53), FN + CHM (SMD 2.10, 95% CI 1.07 to 3.14), and MA + CHM (SMD 2.16, 95% CI 0.97 to 3.35). No statistically significant differences were found among FN, FN + CHM, and MA + CHM (Supplementary Section S8, Table S8.4).

#### 3.6.4. Tumor Necrosis Factor-α

The TNF-α network included 5 studies, 434 participants, and four intervention nodes, with WM as the reference treatment (Figure S6.4). Compared with WM, TNF-α levels were significantly lower with FN (SMD −3.35, 95% CI −3.87 to −2.83), MA + CHM (SMD −3.34, 95% CI −3.81 to −2.87), and MA + WM (SMD −0.80, 95% CI −1.18 to −0.43). FN ranked first by SUCRA (83.6%), closely followed by MA + CHM (83.1%), with MA + WM ranking third (33.3%) (Figure S7.5; Table S7.5). The league table showed no statistically significant difference between FN and MA + CHM (SMD −0.01, 95% CI −0.71 to 0.69), while both FN (SMD −2.55, 95% CI −3.19 to −1.91) and MA + CHM (SMD −2.54, 95% CI −3.14 to −1.94) were favored over MA + WM (Supplementary Section S8, Table S8.5).

#### 3.6.5. Vascular Endothelial Growth Factor

The VEGF network included 4 studies, 413 participants, and three intervention nodes, with WM as the reference treatment (Figure S6.5). Compared with WM, VEGF levels were significantly lower with FN + CHM (SMD −1.15, 95% CI −1.85 to −0.44) and MWA + CHM (SMD −0.76, 95% CI −1.45 to −0.06). FN + CHM ranked first by SUCRA (89.0%), followed by MWA + CHM (60.0%) and WM (1.0%) (Figure S7.6; Table S7.6). The league table showed no statistically significant difference between FN + CHM and MWA + CHM (SMD −0.49, 95% CI −1.40 to 0.42) (Supplementary Section S8, Table S8.6).

#### 3.6.6. Adverse Events

The adverse-event network included 17 studies involving 1513 participants and 12 intervention nodes, with WM as the reference treatment (Figure S6.6). Compared with WM, lower odds of reported adverse events were observed for FN + UR (OR 0.00, 95% CI 0.00–0.11), MA + MOX (OR 0.01, 95% CI 0.00–0.10), MOX + UR (OR 0.01, 95% CI 0.00–0.35), UR (OR 0.01, 95% CI 0.00–0.33), and MA + UR (OR 0.02, 95% CI 0.00–0.48). However, these network estimates were based on sparse events, several zero-event arms, and heterogeneous adverse-event definitions and reporting; therefore, no treatment ranking was presented.

Connectivity between the WM- and UR-based parts of the safety network depended heavily on the MA + MOX node. In particular, Wu 2011 reported 0/30 adverse events with MA + MOX versus 23/30 with WM, producing an extreme direct estimate (OR 0.01, 95% CI 0.00–0.10). By comparison, the direct estimates versus UR were less extreme: FN + UR versus UR, OR 0.30 (95% CI 0.14–0.64); MA + MOX versus UR, OR 0.35 (95% CI 0.13–1.00); MOX + UR versus UR, OR 0.75 (95% CI 0.17–3.33); and MA + UR versus UR, OR 1.19 (95% CI 0.38–3.74). Thus, the very low network odds versus WM for several UR-containing regimens were strongly influenced by this single bridging comparison rather than by uniformly large direct safety effects. Under-ascertainment or selective reporting of adverse events cannot be excluded. Study-level event counts and reported adverse-event types are provided in Supplementary Section S2, Table S2.3. CINeMA certainty-of-evidence assessment was performed for PASI only.

### 3.7. Funnel Plot

Visual inspection of the comparison-adjusted funnel plots suggested possible small-study effects, particularly for PASI, although interpretation was limited by substantial heterogeneity and the small number of studies for several secondary outcomes (Supplementary Section S10).

### 3.8. Post Hoc Sensitivity Analyses

In the comparator-node sensitivity analysis, WM and UR were divided into more specific comparator categories. Two excimer studies became disconnected from the main network and were excluded, leaving 31 connected studies. FN, FN + CHM, and MA + CHM remained statistically significant, whereas FN + MA, MWA + CHM, MA + MOX, MA, and MOX continued to have confidence intervals that included 0. Because the reference definition changed, the primary comparison of MA + WM versus WM (SMD −2.44, 95% CI −4.85 to −0.03) corresponded to MA + WM-S versus WM-T in the sensitivity analysis (SMD −2.32, 95% CI −4.98 to 0.35). MA + WM-S had the highest SUCRA value (81.1%) in this analysis (Supplementary Section S11, Tables S11.2 and S11.3).

In the acupuncture-focused sensitivity analysis, 14 studies containing CHM interventions and Lv Shiqi 2019, which used an active acupuncture comparator, were excluded. The resulting network included 18 studies and 12 connected nodes. The FN estimate was similar to that of the primary analysis (sensitivity analysis: SMD −1.78, 95% CI −3.42 to −0.14; primary analysis: SMD −1.77, 95% CI −3.17 to −0.37). For MA + WM, the point estimate remained −2.44, while the 95% CI widened from −4.85 to −0.04 in the primary analysis to −5.27 to 0.39 in the sensitivity analysis. The FN + MA estimate changed from −1.47 (95% CI −3.44 to 0.51) in the primary analysis to −1.02 (95% CI −3.86 to 1.82) in the sensitivity analysis. FN + UR, MA + WM, and FN remained the three highest-ranked shared interventions, although the FN + UR comparison with WM continued to include 0 (Supplementary Section S11, Tables S11.5 and S11.6).

## 4. Discussion

### 4.1. Comparison with Previous Clinical and Meta-Analytic Evidence

Current psoriasis management remains centred on topical agents, phototherapy, systemic non-biologic treatments, targeted therapies, and biologic agents, selected according to disease severity, lesion distribution, comorbidities, and patient preferences [2,11,12,13,14,15,16,17]. The present findings are broadly consistent with previous clinical evidence suggesting that acupuncture-related treatments may improve skin lesions and patient-reported outcomes in psoriasis, while providing a more treatment-specific interpretation.

The results also extend those of the earlier network meta-analysis by Yeh et al. [29]. That review included 13 randomized controlled trials identified through March 2016 and grouped a wide range of interventions, including acupuncture, acupressure, bloodletting, catgut embedding, medication, and phototherapy, into relatively broad treatment categories. In contrast, the present review included 33 randomized controlled trials involving 2711 participants and defined treatment nodes according to the components actually administered. This approach separated acupuncture monotherapy from combinations of acupuncture techniques and from add-on regimens containing Chinese herbal medicine, Western medicine, or ultraviolet radiation. As a result, the present analysis does not reproduce the treatment hierarchy reported in the earlier review; instead, favorable PASI estimates were observed for MA + WM, MA + CHM, FN, and FN + CHM compared with WM. These differences may reflect the later search period, revised eligibility criteria, and, importantly, the more clinically specific definition of treatment nodes.

Our findings are also partly consistent with more recent pairwise evidence on fire-needle-based combination therapy. A 2024 systematic review reported that fire needle combined with Chinese herbal medicine was associated with improvements in PASI, pruritus, and DLQI compared with Chinese herbal medicine alone [66]. This is directionally compatible with the favorable estimates observed for FN + CHM in the present network. However, the comparisons are not equivalent: the previous pairwise analysis mainly evaluated the incremental effect of adding fire needling to Chinese herbal medicine, whereas the present network compared several complete treatment regimens across different network comparators. The current findings should therefore not be interpreted as a direct replication of the earlier pooled effects.

An important difference from previous reviews is the greater uncertainty demonstrated in the present analysis. Substantial heterogeneity was observed in the PASI network (τ^2^ = 1.46; I^2^ = 93.83%). Differences in disease duration, treatment duration, acupuncture protocols, concomitant therapies, and comparator definitions may have contributed to this variability. Although MA + WM, MA + CHM, FN, and FN + CHM significantly reduced PASI compared with WM, all corresponding 95% prediction intervals crossed the prespecified equivalence range, and CINeMA rated these comparisons as very-low-certainty evidence.

In addition, FN + UR had the highest SUCRA value but did not differ significantly from WM. The post hoc sensitivity analyses further showed that some rankings and the statistical significance of MA + WM changed when comparator nodes or the eligible intervention set were modified. These findings indicate that treatment ranking alone provides an incomplete summary of the comparative evidence and may partly explain differences from earlier meta-analytic findings.

### 4.2. Potential Biological Mechanisms

Psoriasis is driven by complex interactions among keratinocytes, immune cells, inflammatory cytokines, and the cutaneous vasculature, with the IL-23/Th17 axis and TNF-α playing central roles in sustaining inflammation [6,7,67]. In the present analysis, FN, MA + CHM, and MA + WM were associated with lower TNF-α levels than WM. Experimental psoriasis models further support the importance of inflammatory signaling pathways, including IL-17- and TNF-related mechanisms [68,69]. Acupuncture-related experiments have also suggested reductions in local inflammatory responses and modulation of immune-cell activity [8,27,28]. These findings are biologically compatible with an immunomodulatory effect, but the clinical biomarker data do not establish that changes in TNF-α mediate improvements in PASI or other clinical outcomes. Moreover, because MA + CHM and MA + WM are combination regimens, their effects cannot be attributed to acupuncture alone.

Vascular changes may represent another relevant pathway. VEGF contributes to angiogenesis and the abnormal microvascular changes observed in psoriatic lesions [70,71]. In this review, VEGF levels were lower with FN + CHM and MWA + CHM than with WM. These findings are consistent with the possibility that acupuncture-related regimens may influence local inflammatory and vascular processes. However, both interventions contained Chinese herbal medicine, and the available trials did not allow the contribution of individual treatment components to be separated. Differences in laboratory methods, sampling times, and the small number of contributing studies further limit mechanistic interpretation.

Pruritus reflects interactions among the skin, immune system, and peripheral nervous system. Substance P and related neuroimmune signalling have been implicated in psoriasis-associated itching, and experimental work has suggested that acupuncture stimulation may influence the substance P–neurokinin-1 receptor pathway [8]. In the present network, MWA + CHM, FN, FN + CHM, and MA + CHM were associated with lower Pruritus Scores than WM, with MWA + CHM ranking highest. Nevertheless, the Pruritus Score network showed statistical evidence of global inconsistency, and three of the four favorable treatments contained Chinese herbal medicine. The observed clinical effects therefore support further investigation of neuroimmune mechanisms but do not establish a specific mechanism attributable to acupuncture.

### 4.3. Clinical Implications

Several clinical implications arise from these findings. First, acupuncture-related therapies should not be treated as a single homogeneous intervention. The present network distinguished acupuncture monotherapy from acupuncture combinations and add-on regimens, and their effects differed across outcomes. These differences suggest that the clinically relevant unit of interpretation is the complete treatment regimen rather than the acupuncture technique in isolation.

Second, treatment rankings should be interpreted together with effect estimates and their uncertainty rather than as evidence of a clinically definitive best treatment. Because CINeMA was applied only to PASI, the certainty of the secondary-outcome network estimates was not formally graded.

Third, the present findings are more consistent with a possible adjunctive role for acupuncture-related regimens than with replacement of established psoriasis treatment. The favorable signals observed in the present network may justify further evaluation of these regimens as adjunctive options for selected patients; however, the very low certainty of the principal PASI comparisons, substantial heterogeneity, and sensitivity of some results to node definition preclude firm treatment recommendations.

The safety findings also require caution. Several regimens showed lower reported odds of adverse events than WM, but adverse events were sparse and some estimates were extreme or imprecise. These findings suggest possible differences in reported tolerability rather than establishing a reliable safety hierarchy.

### 4.4. Strengths and Limitations

This study has several strengths. First, it updates the evidence base through March 2026 and includes 33 randomized controlled trials involving 2711 participants, substantially expanding the evidence available in the previous network meta-analysis. Second, treatment nodes were reconstructed according to the components actually administered. This avoided classifying an acupuncture add-on regimen as acupuncture monotherapy and allowed clinically distinct combinations involving Chinese herbal medicine, Western medicine, and ultraviolet radiation to be represented separately. Third, the review evaluated a broad range of outcomes, including PASI, total response rate, DLQI, Pruritus Score, inflammatory markers, VEGF, and adverse events. Finally, the reliability of the primary findings was examined using RoB 2, CINeMA, prediction intervals, heterogeneity and inconsistency analyses, and two post hoc sensitivity analyses that specifically tested the influence of comparator-node definitions and restriction to a more acupuncture-focused network.

Several limitations should also be considered. First, risk-of-bias concerns remained common, particularly for deviations from intended interventions and outcome measurement. Because blinding of participants and practitioners is difficult in acupuncture trials, subjective outcomes such as DLQI and Pruritus Score may have been especially susceptible to knowledge of treatment allocation. Second, heterogeneity was substantial for PASI and several secondary outcomes. Definitions of total response rate varied across trials. Although most studies used PASI improvement thresholds of ≥60% or ≥70%, several trials used other clinical or syndrome-based response criteria. Pooling these author-defined responder outcomes may therefore have introduced additional clinical heterogeneity, and the total response rate should not be interpreted as a uniform PASI-response endpoint. Statistical evidence of global inconsistency was detected for Pruritus Score and TNF-α. Local inconsistency could not be evaluated for DLQI, Pruritus Score, TNF-α, or VEGF because these networks contained no closed loops. These findings further limit confidence in the relative treatment estimates for these outcomes.

Third, CINeMA was applied only to PASI, so the certainty of evidence for secondary outcomes was not formally graded. Even for PASI, the statistically significant comparisons with WM were rated as very low certainty, and all corresponding prediction intervals crossed the prespecified equivalence range.

Fourth, several nodes represented combination regimens, particularly those containing Chinese herbal medicine, Western medicine, or ultraviolet radiation. Although this node structure more accurately reflects what was actually tested, it also means that the effects of the individual components cannot be separated. The two post hoc sensitivity analyses partly addressed this issue, but they also showed that some findings, including the MA + WM comparison and treatment rankings, were sensitive to changes in network definition.

Finally, all included studies were conducted in China and generally had modest sample sizes and short treatment periods. Long-term follow-up was limited, preventing firm conclusions regarding recurrence, sustained benefit, and long-term safety. Some treatment nodes were supported by few trials, and splitting UR into NB-UVB and excimer therapy caused the excimer studies to form a disconnected component in the sensitivity analysis. Treatment-duration meta-regression was not performed because many network contrasts were supported by few studies and treatment duration showed limited replication within individual contrasts, making a study-level network meta-regression vulnerable to unstable estimates and ecological bias. Treatment duration was instead examined as a potential effect modifier in the transitivity assessment. Consequently, the extent to which treatment effects vary according to disease severity, treatment duration, acupuncture protocol, or other patient characteristics remains uncertain.

## 5. Conclusions

This updated network meta-analysis suggests that acupuncture-related monotherapies and add-on regimens may provide different benefits across clinically relevant outcomes in psoriasis vulgaris. MA + WM, MA + CHM, FN, and FN + CHM showed favorable effects on PASI, while different regimens demonstrated potential benefits for treatment response, quality of life, pruritus, biomarkers, and safety. However, no single treatment was consistently superior across outcomes, and the findings should be interpreted cautiously because of heterogeneity, the very low certainty of PASI evidence, and sensitivity to network definitions. Further well-designed multicenter trials with standardized interventions, outcome measures, longer follow-up, and more geographically diverse populations are warranted.

## Figures and Tables

**Table 1:** Baseline characteristics of the included studies.

Study	Treatment Duration	Participants, n	Randomized Treatments	Age, Years (mean ± SD)	Disease Duration, Years (mean ± SD)
Wu 2011, [36]	12 weeks	60	MA + MOX: 30 WM: 30	37.38 ± 11.36 39.25 ± 10.21	0.76 ± 0.61 0.72 ± 0.54
Han 2014, [37]	3 weeks	80	MOX + UR: 40 UR: 40	46.2 ± 6.8 45.6 ± 7.3	3.98 ± 0.90 3.62 ± 0.87
Jie 2015, [38]	8 weeks	80	MA: 40 WM: 40	38.28 ± 10.89 41.37 ± 11.04	12.78 ± 8.26 13.56 ± 8.71
Zhang Cuixia 2016, [39]	8 weeks	95	FN + UR: 50 UR: 45	33.2 ± 10.6 31.2 ± 9.7	6.1 ± 2.4 6.5 ± 2.1
Zhang Chen 2016, [40]	8 weeks	76	FN + CHM: 38 WM: 38	51.12 ± 3.87 53.02 ± 3.31	7.12 ± 1.02 6.54 ± 1.42
Deng 2017, [41]	4 weeks	60	MOX: 30 WM: 30	40.4 ± 10.9 41.7 ± 11.5	5.7 ± 10.6 5.4 ± 11.1
Luo 2017, [42]	3 weeks	40	MOX + UR: 20 UR: 20	37.36 ± 11.32 36.96 ± 12.02	0.75 ± 0.60 0.74 ± 0.64
Wei 2017, [43]	8 weeks	55	MA + CHM: 27 WM: 28	32.73 ± 5.36 33.24 ± 5.19	4.45 ± 2.38 4.39 ± 2.31
Hu 2018, [44]	30 days	120	FN + CHM: 60 WM: 60	32.2 ± 11.6 32.7 ± 9.9	5.2 ± 3.4 5.4 ± 2.3
Lv 2019, [45]	2 months	80	FN + MA: 40 MA: 40	38 ± 6 39 ± 6	4.28 ± 2.72 4.33 ± 1.98
Wang 2019, [32]	4 weeks	133	FN + CHM: 67 WM: 66	42.6 ± 12.5 41.9 ± 11.1	3.3 ± 1.4 3.4 ± 1.6
Huang 2020, [46]	12 weeks	80	MA + MOX: 40 UR: 40	46.93 ± 4.11 45.62 ± 3.57	4.14 ± 1.72 4.52 ± 2.98
Tong 2020, [47]	5 weeks	96	FN + UR: 48 UR: 48	38.05 ± 5.44 39.48 ± 5.28	2.37 ± 1.12 2.32 ± 1.34
Chen 2021, [48]	8 weeks	74	MOX: 38 WM: 36	43 ± 9 41 ± 12	13.6 ± 9.8 15.8 ± 12.0
Meng 2021, [49]	30 days	90	FN + WM: 45 WM: 45	38.6 ± 11.3 38.6 ± 11.3	14.2 ± 9.5 14.2 ± 9.5
Jiang 2022, [50]	8 weeks	63	MA + UR: 31 UR: 32	38 ± 9 37 ± 8	10.93 ± 2.89 10.03 ± 3.92
Xiao 2022a, [51]	4 weeks	62	FN: 31 WM: 31	42.13 ± 15.20 45.24 ± 14.02	0.51 ± 0.32 0.48 ± 0.25
Yin 2022, [52]	8 weeks	70	FN + MA: 35 WM: 35	40.57 ± 7.88 43.80 ± 6.56	7.74 ± 2.85 8.43 ± 3.69
Zhou 2022, [53]	8 weeks	65	MA + UR: 32 UR: 33	40.09 ± 6.34 40.01 ± 6.23	9.96 ± 3.81 9.88 ± 3.75
Xiao 2022b, [54]	8 weeks	80	FN: 40 WM: 40	42 ± 16 45 ± 14	0.52 ± 0.39 0.47 ± 0.27
Tian 2023, [33]	3 months	112	FN + CHM: 56 WM: 56	40.47 ± 6.85 38.74 ± 6.63	3.45 ± 0.61 3.31 ± 0.57
Tong 2023, [55]	4 weeks	97	FN + CHM: 48 WM: 49	39.6 ± 6.89 37.8 ± 6.53	2.03 ± 1.15 2.17 ± 1.30
Wang 2023, [56]	12 weeks	124	MWA + CHM: 62 WM: 62	32.31 ± 4.46 32.45 ± 4.11	8.30 ± 2.18 8.32 ± 2.12
Yuan 2023, [57]	3 months	86	MA + CHM: 43 WM: 43	39.05 ± 4.16 38.96 ± 4.12	6.62 ± 1.37 6.58 ± 1.34
Cui 2024, [58]	12 weeks	120	MA + WM: 60 WM: 60	36.9 ± 6.3 37.8 ± 6.5	4.6 ± 2.5 4.7 ± 2.6
Qi 2024, [59]	8 weeks	130	MA + UR: 65 UR: 65	46.17 ± 10.69 48.24 ± 11.23	9.63 ± 4.87 9.19 ± 5.61
Wang 2024, [34]	1 month	60	FN + CHM: 30 WM: 30	45.39 ± 9.47 43.76 ± 8.83	2.96 ± 0.80 2.68 ± 0.70
Yang 2024, [60]	4 weeks	58	FN + CHM: 28 WM: 30	40.1 ± 4.79 35.2 ± 3.91	2.11 ± 1.05 2.02 ± 1.20
Zuo 2024, [61]	12 weeks	80	MWA + CHM: 40 WM: 40	46.05 ± 5.91 46.24 ± 6.11	6.51 ± 1.17 6.63 ± 1.21
Gao 2025, [62]	8 weeks	86	MA + CHM: 43 WM: 43	40.20 ± 5.40 39.85 ± 5.59	1.21 ± 0.24 1.28 ± 0.26
Huang 2025, [63]	4 weeks	100	FN + CHM: 50 WM: 50	41.23 ± 3.19 41.52 ± 3.69	3.96 ± 0.77 3.87 ± 0.62
Li 2025, [64]	8 weeks	60	FN: 30 WM: 30	31.77 ± 10.06 27.84 ± 8.45	8.39 ± 2.19 8.22 ± 2.55
Wang 2025, [65]	8 weeks	39	FN + CHM: 20 WM: 19	36.60 ± 10.58 38.74 ± 11.07	11.87 ± 5.97 10.21 ± 5.15

Abbreviations: CHM, Chinese herbal medicine; FN, fire needle; MA, manual acupuncture; MOX, moxibustion; MWA, microwave acupuncture; UR, ultraviolet radiation; WM, Western medicine; SD, standard deviation. All included trials were randomized controlled trials enrolling participants with stable psoriasis vulgaris or plaque psoriasis.

## Data Availability

No new data were created or analyzed in this study.

## Supplementary Materials

The following supporting information can be downloaded at: https://www.mdpi.com/article/10.3390/healthcare14213718/s1. Supplementary Materials contains the complete search strategies, characteristics of included studies and intervention details, extracted variables, study-level RoB 2 assessments, inconsistency and heterogeneity analyses, secondary-outcome network plots and forest plots, SUCRA rankings, league tables, CINeMA assessments, funnel plots, sensitivity analyses, and the PRISMA-NMA checklist.

## Abbreviations

CHM, Chinese herbal medicine; CINeMA, Confidence in Network Meta-Analysis; DLQI, Dermatology Life Quality Index; FN, fire-needle acupuncture; MA, manual acupuncture; MD, mean difference; MOX, moxibustion; MWA, microwave acupuncture; NB-UVB, narrowband ultraviolet B; NMA, network meta-analysis; OR, odds ratio; PASI, Psoriasis Area and Severity Index; PrI, prediction interval; RCT, randomized controlled trial; RoB 2, revised Cochrane risk-of-bias tool for randomized trials; RR, relative risk; SMD, standardized mean difference; SUCRA, surface under the cumulative ranking curve; TNF-α, tumor necrosis factor-α; UR, ultraviolet radiation; VEGF, vascular endothelial growth factor; WM, Western medicine; WM-S, systemic Western medicine; WM-T, topical Western medicine; WM-TS, combined topical and systemic Western medicine.
