# Supplementary material for: Comparative Effectiveness and Safety of Acupuncture-Related Therapies for Psoriasis Vulgaris: An Updated Systematic Review and Network Meta-Analysis
Source: Healthcare (Basel). 2026 Nov 2;14(21):3718. doi: 10.3390/healthcare14213718 (PMC13763462; doi:10.3390/healthcare14213718)
Supplement: Supplementary file 1 [file healthcare-14-03718-s001.zip › healthcare-4607517-supplementary.pdf]

Supplementary Materials

Comparative Effectiveness and Safety of Acupuncture-Related Therapies for Psoriasis Vulgaris: An Updated Systematic Review and Network Meta-analysis

Table of contents

Supplementary Section S1: Search strategy 2
Supplementary Section S2: Characteristics of included studies 11
Supplementary Section S3: Variables extracted from the included randomized controlled trials 24
Supplementary Section S4: Risk of bias of randomized clinical trials 25
Supplementary Section S5: Evaluation of inconsistency and heterogeneity 27
Supplementary Section S6: Network maps and forest plots of secondary outcomes 31
Supplementary Section S7: SUCRA and cumulative ranking plots 37
Supplementary Section S8: League tables of network meta-analysis estimates 51
Supplementary Section S9: CINeMA assessment 58
Supplementary Section S10: Funnel plots 65
Supplementary Section S11: Sensitivity analyses 69
Supplementary Section S12: PRISMA-NMA checklist 77

Supplementary Section S1: Search strategy

Table S1.1. Search strategy for PubMed.

#	Searches
1	(((((acupuncture[MeSH Terms]) OR (acupuncture points[Title/Abstract])) OR (acupuncture therapy[Title/Abstract])) OR (electroacupuncture[Title/Abstract])) OR (fire needle[Title/Abstract])) OR (moxibustion[Title/Abstract])
2	(((((Psoriasis[MeSH Terms]) OR (Psoriases[Title/Abstract])) OR (Pustulosis of Palms[Title/Abstract] AND Soles[Title/Abstract])) OR (Pustulosis Palmaris et Plantaris[Title/Abstract])) OR (Palmoplantaris Pustulosis[Title/Abstract])) OR (Pustular Psoriasis of Palms and Soles[Title/Abstract])
3	#1 AND #2
4	((((meta analysis[MeSH Terms]) OR (meta analysis as topic[MeSH Terms])) OR (meta analysis[Publication Type])) OR (review[Publication Type])) OR (meta analysis[Title/Abstract])
5	#3 NOT #4
6	(((((((randomized controlled trial[Publication Type]) OR (controlled clinical trial[Publication Type])) OR (clinical trials as topic[MeSH Terms])) OR (random allocation[MeSH Terms])) OR (randomized controlled trials as topic[MeSH Terms])) OR (clinical trial[Publication Type])) OR (clinical trial[Text Word]))
7	#5 AND #6

Table S1.2. Search strategy for the Cochrane Central Register of Controlled Trials (CENTRAL).
(CENTRAL)

#	Searches
1	MeSH descriptor: [Acupuncture Therapy] explode all trees OR MeSH descriptor: [Acupuncture Points] explode all trees OR MeSH descriptor: [Electroacupuncture] explode all trees OR MeSH descriptor: [Moxibustion] explode all trees OR acupuncture:ti,ab,kw OR "acupuncture point*":ti,ab,kw OR "acupuncture therap*":ti,ab,kw OR electroacupuncture:ti,ab,kw OR "electro-acupuncture":ti,ab,kw OR "fire needle*":ti,ab,kw OR "fire needling":ti,ab,kw OR "fire acupuncture":ti,ab,kw OR moxibustion:ti,ab,kw
2	MeSH descriptor: [Psoriasis] explode all trees OR psoriasi*:ti,ab,kw OR psoriases:ti,ab,kw OR "pustulosis of palms and soles":ti,ab,kw OR "pustulosis palmaris et plantaris":ti,ab,kw OR "palmoplantar pustulosis":ti,ab,kw OR "palmoplantaris pustulosis":ti,ab,kw OR "pustular psoriasis of palms and soles":ti,ab,kw
3	#1 AND #2
4	MeSH descriptor: [Meta-Analysis] explode all trees OR MeSH descriptor: [Review] explode all trees OR "meta analysis":ti,ab,kw OR meta-analysis:ti,ab,kw OR metaanalysis:ti,ab,kw OR review:ti,ab,kw OR "systematic review":ti,ab,kw
5	#3 NOT #4
6	MeSH descriptor: [Randomized Controlled Trials as Topic] explode all trees OR MeSH descriptor: [Random Allocation] explode all trees OR MeSH descriptor: [Clinical Trials as Topic] explode all trees OR random*:ti,ab,kw OR randomized:ti,ab,kw OR randomly:ti,ab,kw OR placebo:ti,ab,kw OR trial:ti,ab,kw OR controlled:ti,ab,kw
7	#5 AND #6

Table S1.3. Search strategy for Embase.

#	Searches
1	exp acupuncture/ OR exp acupuncture point/ OR exp acupuncture therapy/ OR exp electroacupuncture/ OR exp moxibustion/ OR acupuncture.ti,ab. OR acupuncture point*.ti,ab. OR acupuncture therap*.ti,ab. OR electroacupuncture.ti,ab. OR electro-acupuncture.ti,ab. OR fire needle*.ti,ab. OR fire needling.ti,ab. OR fire acupuncture.ti,ab. OR moxibustion.ti,ab.
2	exp psoriasis/ OR psoriasi*.ti,ab. OR psoriases.ti,ab. OR palmoplantar pustulosis.ti,ab. OR pustulosis palmaris et plantaris.ti,ab. OR pustulosis of palms and soles.ti,ab. OR pustular psoriasis of palms and soles.ti,ab.
3	#1 AND #2
4	exp meta analysis/ OR exp systematic review/ OR meta analysis.pt. OR systematic review.pt. OR review.pt. OR meta analys*.ti,ab. OR metaanalys*.ti,ab. OR systematic review*.ti,ab. OR review.ti.
5	#3 NOT #4
6	exp randomized controlled trial/ OR exp controlled clinical trial/ OR exp clinical trial/ OR exp randomization/ OR randomized controlled trial.pt. OR controlled clinical trial.pt. OR clinical trial.pt. OR random*.ti,ab. OR randomly.ti,ab. OR trial.ti,ab. OR placebo.ti,ab. OR controlled.ti,ab.
7	#5 AND #6

Table S1.4. Search strategy for Web of Science.

#	**Searches**
1	TS=(acupuncture OR "acupuncture point*" OR "acupuncture therap*" OR electroacupuncture OR "electro-acupuncture" OR "fire needle*" OR "fire needling" OR "fire acupuncture" OR moxibustion)
2	TS=(psoriasis OR psoriases OR psoriatic OR "pustulosis of palms and soles" OR "pustulosis palmaris et plantaris" OR "palmoplantar pustulosis" OR "palmoplantaris pustulosis" OR "pustular psoriasis of palms and soles")
3	#1 AND #2
4	TS=("meta analysis" OR meta-analysis OR metaanalysis OR "systematic review" OR review) OR DT=(Review)
5	#3 NOT #4
6	TS=(random* OR randomized OR randomised OR randomly OR placebo OR trial OR "clinical trial" OR "controlled trial" OR "randomized controlled trial" OR "randomised controlled trial" OR RCT)
7	#5 AND #6

Table S1.5. Search strategy for Scopus.

#	Searches
1	TITLE-ABS-KEY(acupuncture OR "acupuncture point*" OR "acupuncture therap*" OR electroacupuncture OR "electro-acupuncture" OR "fire needle*" OR "fire needling" OR "fire acupuncture" OR moxibustion)
2	TITLE-ABS-KEY(psoriasis OR psoriases OR psoriatic OR "pustulosis of palms and soles" OR "pustulosis palmaris et plantaris" OR "palmoplantar pustulosis" OR "palmoplantaris pustulosis" OR "pustular psoriasis of palms and soles")
3	#1 AND #2
4	TITLE-ABS-KEY("meta analysis" OR meta-analysis OR metaanalysis OR "systematic review" OR review) OR DOCTYPE(re)
5	#3 NOT #4
6	TITLE-ABS-KEY(random* OR randomized OR randomised OR randomly OR placebo OR trial OR "clinical trial" OR "controlled trial" OR "randomized controlled trial" OR "randomised controlled trial" OR RCT)
7	#5 AND #6

Table S1.6. Search strategy for China National Knowledge Infrastructure (CNKI).

#	Searches
1	(SU='银屑病' OR SU='寻常型银屑病' OR SU='寻常性银屑病' OR SU='斑块型银屑病' OR SU='白疕' OR SU='牛皮癣' OR SU='掌跖脓疱病' OR SU='掌跖脓疱症' OR SU='掌跖脓疱性银屑病' OR SU='掌跖脓疱型银屑病')
2	(SU='针灸' OR SU='针刺' OR SU='针刺疗法' OR SU='针灸疗法' OR SU='毫针' OR SU='体针' OR SU='火针' OR SU='火针疗法' OR SU='电针' OR SU='电针疗法' OR SU='艾灸' OR SU='灸法' OR SU='温针灸' OR SU='温针' OR SU='梅花针' OR SU='皮肤针' OR SU='微波针灸' OR SU='针' OR SU='灸' OR SU='穴')
3	#1 AND #2
4	(SU='Meta分析' OR SU='荟萃分析' OR SU='系统评价' OR SU='系统综述' OR SU='综述')
5	#3 NOT #4
6	(SU='随机对照试验' OR SU='随机对照研究' OR SU='随机临床试验' OR SU='随机' OR SU='临床试验' OR SU='对照试验')
7	#5 AND #6

Table S1.7. Search strategy for the Chinese Science and Technology Periodical Database (VIP).

#	Searches
1	(T=(银屑病 OR 寻常型银屑病 OR 寻常性银屑病 OR 斑块型银屑病 OR 白疕 OR 牛皮癣 OR 掌跖脓疱病 OR 掌跖脓疱症 OR 掌跖脓疱性银屑病 OR 掌跖脓疱型银屑病) OR K=(银屑病 OR 寻常型银屑病 OR 寻常性银屑病 OR 斑块型银屑病 OR 白疕 OR 牛皮癣 OR 掌跖脓疱病 OR 掌跖脓疱症 OR 掌跖脓疱性银屑病 OR 掌跖脓疱型银屑病) OR R=(银屑病 OR 寻常型银屑病 OR 寻常性银屑病 OR 斑块型银屑病 OR 白疕 OR 牛皮癣 OR 掌跖脓疱病 OR 掌跖脓疱症 OR 掌跖脓疱性银屑病 OR 掌跖脓疱型银屑病))
2	(T=(针灸 OR 针刺 OR 针刺疗法 OR 针灸疗法 OR 毫针 OR 体针 OR 火针 OR 火针疗法 OR 电针 OR 电针疗法 OR 艾灸 OR 灸法 OR 温针灸 OR 温针 OR 梅花针 OR 皮肤针 OR 微波针灸 OR 针 OR 灸 OR 穴) OR K=(针灸 OR 针刺 OR 针刺疗法 OR 针灸疗法 OR 毫针 OR 体针 OR 火针 OR 火针疗法 OR 电针 OR 电针疗法 OR 艾灸 OR 灸法 OR 温针灸 OR 温针 OR 梅花针 OR 皮肤针 OR 微波针灸 OR 针 OR 灸 OR 穴) OR R=(针灸 OR 针刺 OR 针刺疗法 OR 针灸疗法 OR 毫针 OR 体针 OR 火针 OR 火针疗法 OR 电针 OR 电针疗法 OR 艾灸 OR 灸法 OR 温针灸 OR 温针 OR 梅花针 OR 皮肤针 OR 微波针灸 OR 针 OR 灸 OR 穴))
3	#1 AND #2
4	(T=(Meta分析 OR 荟萃分析 OR 系统评价 OR 系统综述 OR 综述) OR K=(Meta分析 OR 荟萃分析 OR 系统评价 OR 系统综述 OR 综述) OR R=(Meta分析 OR 荟萃分析 OR 系统评价 OR 系统综述 OR 综述))
5	#3 NOT #4
6	(T=(随机对照试验 OR 随机对照研究 OR 随机临床试验 OR 随机 OR 临床试验 OR 对照试验) OR K=(随机对照试验 OR 随机对照研究 OR 随机临床试验 OR 随机 OR 临床试验 OR 对照试验) OR R=(随机对照试验 OR 随机对照研究 OR 随机临床试验 OR 随机 OR 临床试验 OR 对照试验))
7	#5 AND #6

Table S1.8. Search strategy for Wanfang Data.

#	Searches
1	主题:("银屑病" OR "寻常型银屑病" OR "寻常性银屑病" OR "斑块型银屑病" OR "白疕" OR "牛皮癣" OR "掌跖脓疱病" OR "掌跖脓疱症" OR "掌跖脓疱性银屑病" OR "掌跖脓疱型银屑病")
2	主题:("针灸" OR "针刺" OR "针刺疗法" OR "针灸疗法" OR "毫针" OR "体针" OR "火针" OR "火针疗法" OR "电针" OR "电针疗法" OR "艾灸" OR "灸法" OR "温针灸" OR "温针" OR "梅花针" OR "皮肤针" OR "微波针灸" OR "针" OR "灸" OR "穴")
3	#1 AND #2
4	主题:("Meta分析" OR "荟萃分析" OR "系统评价" OR "系统综述" OR "综述")
5	#3 NOT #4
6	主题:("随机对照试验" OR "随机对照研究" OR "随机临床试验" OR "随机" OR "临床试验" OR "对照试验")
7	#5 AND #6

Table S1.9. Search strategy for the Chinese Biomedical Literature Database (SinoMed).

#	Searches
1	(银屑病[常用字段] OR 寻常型银屑病[常用字段] OR 寻常性银屑病[常用字段] OR 斑块型银屑病[常用字段] OR 白疕[常用字段] OR 牛皮癣[常用字段] OR 掌跖脓疱病[常用字段] OR 掌跖脓疱症[常用字段] OR 掌跖脓疱性银屑病[常用字段] OR 掌跖脓疱型银屑病[常用字段])
2	(针灸[常用字段] OR 针刺[常用字段] OR 针刺疗法[常用字段] OR 针灸疗法[常用字段] OR 毫针[常用字段] OR 体针[常用字段] OR 火针[常用字段] OR 火针疗法[常用字段] OR 电针[常用字段] OR 电针疗法[常用字段] OR 艾灸[常用字段] OR 灸法[常用字段] OR 温针灸[常用字段] OR 温针[常用字段] OR 梅花针[常用字段] OR 皮肤针[常用字段] OR 微波针灸[常用字段] OR 针[常用字段] OR 灸[常用字段] OR 穴[常用字段])
3	#1 AND #2
4	(Meta分析[常用字段] OR 荟萃分析[常用字段] OR 系统评价[常用字段] OR 系统综述[常用字段] OR 综述[常用字段])
5	#3 NOT #4
6	(随机对照试验[常用字段] OR 随机对照研究[常用字段] OR 随机临床试验[常用字段] OR 随机[常用字段] OR 临床试验[常用字段] OR 对照试验[常用字段])
7	#5 AND #6

Supplementary Section S2: Characteristics of included studies

Table S2.1. Baseline characteristics of included studies.

Study	Design	Treatment duration	Number of participants	Randomised treatments	Age (mean ± SD), years	Disease duration (mean ± SD), years	Clinical stage/type
Wu 2011	RCT	12 weeks	60	MA+MOX: 30<br>WM: 30	37.38±11.36<br>39.25±10.21	0.76±0.61<br>0.72±0.54	Psoriasis vulgaris, stationary stage
Han 2014	RCT	3 weeks	80	MOX+UR: 40<br>UR: 40	46.2±6.8<br>45.6±7.3	3.98±0.90<br>3.62±0.87	Psoriasis vulgaris, stationary stage
Jie 2015	RCT	8 weeks	80	MA: 40<br>WM: 40	38.28±10.89<br>41.37±11.04	12.78±8.26<br>13.56±8.71	Psoriasis vulgaris, stationary stage
Zhang Cuixia 2016	RCT	8 weeks	95	FN+UR: 50<br>UR: 45	33.2±10.6<br>31.2±9.7	6.1±2.4<br>6.5±2.1	Psoriasis vulgaris, stationary stage
Zhang Chen 2016	RCT	8 weeks	76	FN+CHM: 38<br>WM: 38	51.12±3.87<br>53.02±3.31	7.12±1.02<br>6.54±1.42	Psoriasis vulgaris, stationary stage
Deng 2017	RCT	4 weeks	60	MOX: 30<br>WM: 30	40.4±10.9<br>41.7±11.5	5.7±10.6<br>5.4±11.1	Psoriasis vulgaris, stationary stage
Luo 2017	RCT	3 weeks	40	MOX+UR: 20<br>UR: 20	37.36±11.32<br>36.96±12.02	0.75±0.60<br>0.74±0.64	Psoriasis vulgaris, stationary stage
Wei 2017	RCT	8 weeks	55	MA+CHM: 27<br>WM: 28	32.73±5.36<br>33.24±5.19	4.45±2.38<br>4.39±2.31	Psoriasis vulgaris, stationary stage
Hu 2018	RCT	30 days	120	FN+CHM: 60<br>WM: 60	32.2±11.6<br>32.7±9.9	5.2±3.4<br>5.4±2.3	Psoriasis vulgaris, stationary stage
Lv 2019	RCT	2 months	80	FN+MA: 40<br>MA: 40	38±6<br>39±6	4.28±2.72<br>4.33±1.98	Psoriasis vulgaris, stationary stage

Study	Design	Treatment duration	Number of participants	Randomised treatments	Age (mean ± SD), years	Disease duration (mean ± SD), years	Clinical stage/type
Wang 2019	RCT	4 weeks	133	FN+CHM: 67	42.6±12.5	3.3±1.4	Psoriasis vulgaris,
				WM: 66	41.9±11.1	3.4±1.6	stationary stage
Huang 2020	RCT	12 weeks	80	MA+MOX: 40	46.93±4.11	4.14±1.72	Psoriasis vulgaris,
				UR: 40	45.62±3.57	4.52±2.98	stationary stage
Tong 2020	RCT	5 weeks	96	FN+UR: 48	38.05±5.44	2.37±1.12	Psoriasis vulgaris,
				UR: 48	39.48±5.28	2.32±1.34	stationary stage
Chen 2021	RCT	8 weeks	74	MOX: 38	43±9	13.6±9.8	Psoriasis vulgaris,
				WM: 36	41±12	15.8±12.0	stationary stage
Meng 2021	RCT	30 days	90	FN+WM: 45	38.6±11.3	14.2±9.5	Psoriasis vulgaris,
				WM: 45	38.6±11.3	14.2±9.5	stationary stage
Jiang 2022	RCT	8 weeks	63	MA+UR: 31	38±9	10.93±2.89	Psoriasis vulgaris,
				UR: 32	37±8	10.03±3.92	stationary stage
Xiao 2022a	RCT	4 weeks	62	FN: 31	42.13±15.20	0.51±0.32	Psoriasis vulgaris,
				WM: 31	45.24±14.02	0.48±0.25	stationary stage
Yin 2022	RCT	8 weeks	70	FN+MA: 35	40.57±7.88	7.74±2.85	Psoriasis vulgaris,
				WM: 35	43.80±6.56	8.43±3.69	stationary stage
Zhou 2022	RCT	8 weeks	65	MA+UR: 32	40.09±6.34	9.96±3.81	Psoriasis vulgaris,
				UR: 33	40.01±6.23	9.88±3.75	stationary stage
Xiao 2022b	RCT	8 weeks	80	FN: 40	42±16	0.52±0.39	Psoriasis vulgaris,
				WM: 40	45±14	0.47±0.27	stationary stage
Tian 2023	RCT	3 months	112	FN+CHM: 56	40.47±6.85	3.45±0.61	Psoriasis vulgaris,
				WM: 56	38.74±6.63	3.31±0.57	stationary stage
Tong 2023	RCT	4 weeks	97	FN+CHM: 48	39.6±6.89	2.03±1.15	Psoriasis vulgaris,
				WM: 49	37.8±6.53	2.17±1.30	stationary stage

Study	Design	Treatment duration	Number of participants	Randomised treatments	Age (mean ± SD), years	Disease duration (mean ± SD), years	Clinical stage/type
Wang 2023	RCT	12 weeks	124	MWA+CHM: 62<br>WM: 62	32.31±4.46<br>32.45±4.11	8.30±2.18<br>8.32±2.12	Psoriasis vulgaris, stationary stage
Yuan 2023	RCT	3 months	86	MA+CHM: 43<br>WM: 43	39.05±4.16<br>38.96±4.12	6.62±1.37<br>6.58±1.34	Psoriasis vulgaris, stationary stage
Cui 2024	RCT	12 weeks	120	MA+WM: 60<br>WM: 60	36.9±6.3<br>37.8±6.5	4.6±2.5<br>4.7±2.6	Psoriasis vulgaris, stationary stage
Qi 2024	RCT	8 weeks	130	MA+UR: 65<br>UR: 65	46.17±10.69<br>48.24±11.23	9.63±4.87<br>9.19±5.61	Psoriasis vulgaris, stationary stage
Wang 2024	RCT	1 month	60	FN+CHM: 30<br>WM: 30	45.39±9.47<br>43.76±8.83	2.96±0.80<br>2.68±0.70	Psoriasis vulgaris, stationary stage
Yang 2024	RCT	4 weeks	58	FN+CHM: 28<br>WM: 30	40.1±4.79<br>35.2±3.91	2.11±1.05<br>2.02±1.20	Psoriasis vulgaris, stationary stage
Zuo 2024	RCT	12 weeks	80	MWA+CHM: 40<br>WM: 40	46.05±5.91<br>46.24±6.11	6.51±1.17<br>6.63±1.21	Psoriasis vulgaris, stationary stage
Gao 2025	RCT	8 weeks	86	MA+CHM: 43<br>WM: 43	40.20±5.40<br>39.85±5.59	1.21±0.24<br>1.28±0.26	Psoriasis vulgaris, stationary stage
Huang 2025	RCT	4 weeks	100	FN+CHM: 50<br>WM: 50	41.23±3.19<br>41.52±3.69	3.96±0.77<br>3.87±0.62	Psoriasis vulgaris, stationary stage
Li 2025	RCT	8 weeks	60	FN: 30<br>WM: 30	31.77±10.06<br>27.84±8.45	8.39±2.19<br>8.22±2.55	Psoriasis vulgaris, stationary stage
Wang 2025	RCT	8 weeks	39	FN+CHM: 20<br>WM: 19	36.60±10.58<br>38.74±11.07	11.87±5.97<br>10.21±5.15	Psoriasis vulgaris, stationary stage

Abbreviations: CHM, Chinese herbal medicine; FN, fire needle; MA, manual acupuncture; MOX, moxibustion; MWA, microwave acupuncture; UR, ultraviolet radiation; WM, Western medicine; RCT, randomized controlled trial; SD, standard deviation.

Table S2.2. Detailed descriptions of interventions in studies of acupuncture and moxibustion for psoriasis.

Study	Intervention Group	Detailed Intervention Procedure	Control Group Intervention	Detailed Intervention Procedure	Comparator regimen continued in the intervention arm
Wu 2011	**MA + MOX**	【MA】Filiform-needle acupuncture at back-shu points (Feishu, Geshu, Ganshu, Pishu, Shenshu) and additional points (Hegu, Quchi, Xuehai, etc.); needles retained for 30 min, once every other day.<br>【MOX】Mild moxibustion with a moxa stick at Shenshu and skin lesions, 2–3 min per site, once every other day.	**WM**	【WM】Oral acitretin capsules, 20 mg per dose with meals, once daily for 12 consecutive weeks.	NO
Han 2014	**MOX + UR**	【MOX】Sparrow-pecking moxibustion at Dazhui; mild moxibustion at bilateral Sanyinjiao, Xuehai, Quchi, and skin lesions, 2–3 min per point/site, once every other day.<br>【UR】Whole-body NB-UVB irradiation, initial dose 0.6 J/cm², 3 times per week.	**UR**	【UR】Whole-body NB-UVB irradiation alone, initial dose 0.6 J/cm², 3 times per week for 3 consecutive weeks.	YES
Jie 2015	**MA**	【MA】Filiform-needle acupuncture at back-shu points (Feishu, Xinshu, Geshu, Ganshu, Shenshu) plus surrounding needling of plaque lesions (oblique/horizontal insertion 0.2 cm from the edge); needles retained for 30 min, 3 times per week for 8 weeks.	**WM**	【WM】Topical calcipotriol ointment, once daily for 8 consecutive weeks.	NO
Zhang Cuixia 2016	**FN + UR**	【FN】A 0.5 mm × 25 mm thick fire needle was heated until red-white and rapidly inserted into Ashi points in the lesions (depth 3–5 mm), once weekly for 4 sessions.<br>【UR】308-nm excimer laser irradiation, initial dose 3 × MED, twice weekly for 8 weeks.	**UR**	【UR】308-nm excimer laser irradiation alone, initial dose 3 × MED, twice weekly for 8 consecutive weeks.	YES
Zhang Chen 2016	**FN + CHM**	【FN】A 0.34 mm × 25 mm filiform fire needle was heated until red-white and rapidly inserted vertically into the lesions, once weekly.<br>【CHM】Oral self-formulated Xiaoyin formula, 1 decoction daily, taken morning and evening.	**WM**	【WM】Oral compound glycyrrhizin tablets, 50 mg per dose, 3 times daily for 8 consecutive weeks.	NO

Study	Intervention Group	Detailed Intervention Procedure	Control Group Intervention	Detailed Intervention Procedure	Comparator regimen continued in the intervention arm
Deng 2017	**MOX**	【MOX】Zhuang medicated moxibustion at sunflower-pattern points (around and at the center of the lesion) and at Quchi, Xuehai, Zusanli, etc.; one treatment course per week for 4 consecutive weeks.	**WM**	【WM】Oral compound glycyrrhizin capsules, 2 capsules per dose, 3 times daily for 4 consecutive weeks.	NO
Luo 2017	**MOX + UR**	【MOX】Mild moxibustion with a moxa stick/moxibustion combined with irradiation.<br>【UR】Whole-body NB-UVB irradiation, initial dose 0.25 J/cm², increased by 0.25 J/cm² each session, once every other day for 3 weeks.	**UR**	【UR】NB-UVB irradiation alone, initial dose 0.25 J/cm², once every other day for 3 consecutive weeks.	YES
Wei 2017	**MA + CHM**	【MA】A 0.35 mm × 40 mm filiform needle was inserted horizontally 1 cm outside the lesion margin for surrounding needling, with 3 cm between needles; needles retained for 30 min, 3 times per week.<br>【CHM】Oral Xuefu Zhuyu Decoction, 1 decoction daily in two divided doses (morning and evening) for 56 days.	**WM**	【WM】Topical 0.1% tazarotene cream, once nightly at bedtime for 56 consecutive days.	NO
Hu 2018	**FN + CHM**	【FN】A 0.8 mm medium-thick fire needle was heated until red-white and rapidly inserted into Ashi points (depth 1–2 mm), once every 5 days for 6 sessions.<br>【CHM】Topical Yinqiao Sanhuang ointment applied to the lesions twice daily for 30 consecutive days.	**WM**	【WM】Topical calcipotriol ointment, twice daily for 30 consecutive days.	NO
Lv 2019	**FN + MA**	【FN】A 0.30 mm × 25 mm filiform fire needle was heated until red and used to puncture the lesion surface.<br>【MA】Conventional body acupuncture (alternating Dazhui, Feishu, Geshu, Quchi, Xuehai, etc.); needles retained for 30 min, once daily for 40 days.	**MA**	【MA】Conventional body acupuncture alone, with the same acupoints, manipulation, and 30-min needle retention as the intervention group, once daily for 40 days.	YES

Study	Intervention Group	Detailed Intervention Procedure	Control Group Intervention	Detailed Intervention Procedure	Comparator regimen continued in the intervention arm
Wang 2019	**FN + CHM**	【FN】A 0.4 mm × 45 mm fire needle was heated until red and used to puncture thick plaque areas (0.6 cm between punctures), once every 3 days.<br>【CHM】Oral self-formulated Xiaoyin Decoction, 1 decoction daily in two divided doses (morning and evening).	**WM**	【WM】Topical calcipotriol ointment (twice daily) + oral methotrexate tablets (2.5 mg per dose, 1–2 times weekly) for 4 consecutive weeks.	NO
Huang 2020	**MA + MOX**	【MA】Acupuncture at Geshu, Ganshu, Pishu, Shenshu plus Hegu, Quchi, and Xuehai; needles retained for 25 min, once every other day.<br>【MOX】Mild moxibustion with a moxa stick at affected areas for 2–3 min per session, once every other day for 12 consecutive weeks.	**UR**	【UR】Whole-body ultraviolet phototherapy, once every other day for 12 consecutive weeks.	NO
Tong 2020	**FN + UR**	【FN】A 0.8 mm tungsten-manganese alloy fire needle was heated until white and used to puncture lesions vertically, once weekly.<br>【UR】308-nm excimer laser irradiation, initial dose 150 mJ/cm², once weekly for 5 weeks.	**UR**	【UR】308-nm excimer laser irradiation alone, once weekly at an initial dose of 150 mJ/cm² for 5 consecutive weeks.	YES
Chen 2021	**MOX**	【MOX】A Baixiao moxibustion device was placed at Ashi, Zusanli, Xuehai, and Qihai for moxibustion, 30 min per session, 3 times weekly for 8 consecutive weeks.	**WM**	【WM】Topical calcipotriol ointment, 0.25 g per application, once in the morning and once in the evening for 8 consecutive weeks.	NO
Meng 2021	**FN + WM**	【FN】A fine fire needle was heated until red-white and rapidly inserted into the left target lesion (0.5–1.0 cm between punctures), once every 5 days.<br>【WM】Topical calcipotriol ointment and halometasone ointment (each once daily) for 30 consecutive days.	**WM**	【WM】Topical calcipotriol ointment and halometasone ointment applied to the right target lesion (each once daily) for 30 consecutive days.	YES

Study	Intervention Group	Detailed Intervention Procedure	Control Group Intervention	Detailed Intervention Procedure	Comparator regimen continued in the intervention arm
Jiang 2022	**MA + UR**	【MA】Acupuncture at Quchi, Xuehai, Sanyinjiao, Feishu, and Geshu using even reinforcing-reducing manipulation; needles retained for 30 min, 3 times weekly.<br>【UR】NB-UVB irradiation, initial dose 0.4 J/cm², increased by 0.1 J/cm² each session, 3 times weekly for 8 weeks.	**UR**	【UR】NB-UVB irradiation alone, initial dose 0.4 J/cm², 3 times weekly for 8 consecutive weeks.	YES
Xiao 2022a	**FN**	【FN】At Shen time, acupoints selected according to the midnight-noon ebb-flow method and Ashi points were rapidly punctured with a fire needle, once weekly for 4 sessions (28 days).	**WM**	【WM】Topical calcipotriol ointment applied to affected areas twice daily for 4 consecutive weeks.	NO
Yin 2022	**FN + MA**	【FN】Fire-needle puncturing of plaques on the lower leg, once every 5 days.<br>【MA】Conventional acupuncture at Quchi, Xuehai, Zusanli, and Sanyinjiao; needles retained for 30 min, once every other day for 8 weeks.	**WM**	【WM】Topical halcinonide cream applied to affected areas twice daily for 8 consecutive weeks.	NO
Zhou 2022	**MA + UR**	【MA】Acupuncture at Quchi, Xuehai, Sanyinjiao, Geshu, and Feishu using even reinforcing-reducing manipulation; needles retained for 30 min, once every other day.<br>【UR】NB-UVB phototherapy, initial dose 0.4 J/cm², increased by 0.1 J/cm² each session, 3 times weekly for 8 weeks.	**UR**	【UR】NB-UVB phototherapy alone, initial dose 0.4 J/cm², 3 times weekly for 8 consecutive weeks.	YES
Xiao 2022b	**FN**	【FN】A 0.35 mm × 40 mm fire needle was heated until red and rapidly inserted from the center outward around the lesion (2–5 mm between punctures), once weekly for 8 weeks.	**WM**	【WM】Topical calcipotriol ointment, twice daily for 8 consecutive weeks.	NO
Tian 2023	**FN + CHM**	【FN】Clustered filiform fire needles (5 needles) were heated until red and rapidly used to puncture the lesion surface, once every 3 days, 20 min per session.<br>【CHM】Oral Liangxue Huoxue Decoction granules, 1 dose daily in two divided doses (morning and evening) for 3 months.	**WM**	【WM】Oral acitretin capsules, 30 mg/day for 3 consecutive months.	NO

Study	Intervention Group	Detailed Intervention Procedure	Control Group Intervention	Detailed Intervention Procedure	Comparator regimen continued in the intervention arm
Tong 2023	**FN + CHM**	【FN】A 0.5 mm × 5 cm Panlong fire needle was heated until red and used to puncture plaque lesions (0.2 cm between punctures), once every 7 days for 4 sessions.<br>【CHM】Topical Dailiu ointment applied to affected areas once nightly at bedtime for 4 consecutive weeks.	**WM**	【WM】Topical calcipotriol ointment, once daily for 4 consecutive weeks.	NO
Wang 2023	**MWA + CHM**	【MWA】Acupuncture at Fengchi, Neiguan, Waiguan, Zusanli, and Ashi points, connected to the antenna of a microwave acupuncture device for 20–30 min, once daily.<br>【CHM】Oral Tongbi Qushi Huoxue Decoction, 1 decoction daily in two divided doses (morning and evening) for 12 weeks.	**WM**	【WM】Oral compound glycyrrhizin tablets (3 times daily) + oral acitretin capsules (30 mg/day) for 12 weeks.	NO
Yuan 2023	**MA + CHM**	【MA】Acupuncture at Shenshu, Feishu, Geshu, and Ganshu using even reinforcing-reducing manipulation; needles retained for 20 min, 3 times weekly.<br>【CHM】Oral Niupixuan Decoction, 1 decoction daily in two divided doses (morning and evening) for 3 months.	**WM**	【WM】Oral acitretin capsules (20 mg per dose, twice daily) + topical calcipotriol ointment (twice daily) for 3 months.	NO
Cui 2024	**MA + WM**	【MA】Syndrome-differentiated acupuncture (Xuehai, Geshu, Sanyinjiao, Taixi, Feishu, Shenshu); needles retained for 30 min, once every other day.<br>【WM】Oral compound glycyrrhizin tablets (2 tablets/25 mg per dose, 3 times daily) for 12 weeks.	**WM**	【WM】Oral compound glycyrrhizin tablets (2 tablets/25 mg per dose, 3 times daily) for 12 consecutive weeks.	YES
Qi 2024	**MA + UR**	【MA】Acupuncture at Jiaji points corresponding to the affected zang-fu organs, with Quchi and Xuehai added according to symptoms; needles retained for 20 min, 3 times weekly.<br>【UR】NB-UVB phototherapy, initial dose 70% MED, increased by 10%–20% each session, 3 times weekly for 8 weeks.	**UR**	【UR】NB-UVB phototherapy alone, initial dose 70% MED, 3 times weekly for 8 consecutive weeks.	YES

Study	Intervention Group	Detailed Intervention Procedure	Control Group Intervention	Detailed Intervention Procedure	Comparator regimen continued in the intervention arm
Wang 2024	**FN + CHM**	【FN】A fire needle was heated until red and rapidly inserted into affected areas, followed by compression with a cotton ball for hemostasis, once every 5–7 days.<br>【CHM】Oral Xiaobi Decoction (buffalo horn, Rehmannia root, red peony root, Moutan bark, etc.), 1 decoction daily in three divided doses (morning, noon, and evening) for 1 month.	**WM**	【WM】Topical calcipotriol ointment, twice daily for 1 consecutive month.	NO
Yang 2024	**FN + CHM**	【FN】A 0.5 mm × 5 cm fire needle was heated until red and rapidly inserted into affected areas, once weekly for 4 sessions.<br>【CHM】Topical Daihuang ointment applied to affected areas once nightly at bedtime for 4 consecutive weeks.	**WM**	【WM】Topical calcipotriol ointment, once daily for 4 consecutive weeks.	NO
Zuo 2024	**MWA + CHM**	【MWA】Acupuncture at Waiguan, Neiguan, Fengchi, Zusanli, and Ashi points, connected to a microwave acupuncture device for 20–30 min, once daily.<br>【CHM】Oral Tongbi Qushi Huoxue Decoction, 1 decoction daily in two divided doses (morning and evening) for 12 weeks.	**WM**	【WM】Oral compound glycyrrhizin tablets + oral acitretin capsules (30 mg/day) for 12 consecutive weeks.	NO
Gao 2025	**MA + CHM**	【MA】Acupuncture at Quchi, Xuehai, Zusanli, Sanyinjiao, and Hegu using even reinforcing-reducing manipulation; needles retained for 30 min, 3 times weekly.<br>【CHM】Oral Huaidi Liangxue Decoction, 1 decoction daily in two divided doses (morning and evening) for 8 weeks.	**WM**	【WM】Topical 0.1% halometasone cream, twice daily for 8 consecutive weeks.	NO
Huang 2025	**FN + CHM**	【FN】A 0.35 mm × 40 mm fire needle was heated until red and used for radial surrounding puncture from the center of the lesion, once weekly for 4 sessions.<br>【CHM】Oral modified Qingying Decoction, 1 decoction daily in two divided doses (morning and evening) for 4 weeks.	**WM**	【WM】A thin layer of compound flumethasone ointment, twice daily for 4 consecutive weeks.	NO

Study	Intervention Group	Detailed Intervention Procedure	Control Group Intervention	Detailed Intervention Procedure	Comparator regimen continued in the intervention arm
Li 2025	**FN**	【FN】A customized Lingnan fire needle was heated until bright white and densely inserted into lesions (5 mm between punctures); Wanhua oil was applied to prevent burns, once weekly for 8 weeks.	**WM**	【WM】Topical calcipotriol ointment, twice daily for 8 consecutive weeks.	NO
Wang 2025	**FN + CHM**	【FN】A 0.3 mm × 25 mm filiform fire needle was heated until red and rapidly used to puncture the lesion surface (6 mm between punctures), once every 3 days.<br>【CHM】Oral Twelve-Ingredient Zeqi Granules, 1 sachet per dose, 3 times daily for 8 weeks.	**WM**	【WM】Oral Tripterygium glycoside tablets, 1–1.5 mg/kg/day in 3 divided doses for 8 consecutive weeks.	NO

Table S2.3. Adverse Events Reported in the Included Studies.

Study	Intervention	Adverse events, n/N	Reported adverse events	Comparator	Adverse events, n/N	Reported adverse events
Wu 2011	MA+MOX	0/30	None reported	WM	23/30	Dry mouth (10); pruritus (7); desquamation (6)
Jie 2015	MA	0/39	No adverse events reported	WM	0/40	No adverse events reported
Zhang Cuixia 2016	FN+UR	13/50	Erythema/swelling (8); burning pain (3); blistering (2)	UR	25/45	Pruritus (16); burning pain (4); blistering (5)
Luo 2017	MOX+UR	4/20	Erythema (2); pruritus (1); dryness (1)	UR	5/20	Erythema (3); pruritus (1); dryness (1)
Wei 2017	MA+CHM	4/27	Local redness (3); nausea (1)	WM	2/28	Nausea (2)
Hu 2018	FN+CHM	0/60	None reported	WM	3/60	Local erythema/irritation (3; treatment withdrawal)
Lv 2019	FN+MA	0/40	No serious adverse events reported	MA	0/40	No serious adverse events reported
Wang 2019	FN+CHM	0/67	No serious adverse events reported	WM	0/66	No serious adverse events reported
Huang 2020	MA+MOX	7/40	Pruritus (3); desquamation (1); dry mouth (3)	UR	15/40	Pruritus (5); desquamation (6); dry mouth (4)
Tong 2020	FN+UR	2/48	Rash/pruritus (2)	UR	5/48	Mild pruritus (5)
Chen 2021	MOX	0/38	None reported	WM	3/36	Local skin irritation/burning sensation (3)
Meng 2021	FN+WM	4/45	Transient dizziness (1); mild local infection (3)	WM	2/45	Erythema (2)
Tian 2023	FN+CHM	2/56	Gastrointestinal reaction (1); rash (1)	WM	4/56	Gastrointestinal reaction (2); rash (1); folliculitis (1)
Tong 2023	FN+CHM	0/48	None reported	WM	1/49	Worsened erythema/irritation (1; treatment withdrawal)
Yuan 2023	MA+CHM	8/43	Nausea (4); vomiting (3); fatigue (1)	WM	6/43	Nausea (3); vomiting (2); fatigue (1)
Qi 2024	MA+UR	7/65	Dizziness (1); pruritus (2); dryness (2); pain (2)	UR	6/65	Dizziness (1); pruritus (3); dryness (1); pain (1)
Gao 2025	MA+CHM	6/43	Dryness (1); pruritus (2); desquamation (1); dizziness (2)	WM	8/43	Dryness (2); pruritus (3); desquamation (2); dizziness (1)

Abbreviations: CHM, Chinese herbal medicine; FN, fire-needle acupuncture; MA, manual acupuncture; MOX, moxibustion; UR, ultraviolet radiation/phototherapy; WM, Western medicine.

Notes: Adverse-event definitions and reporting varied across studies. Where the original report stated only that no serious adverse events occurred, that wording was retained rather than interpreted as absence of all adverse events. Zero-event cells were retained in the network meta-analysis using the default 0.5 continuity correction implemented in the Stata network suite.

Table S2.4. Original Definitions of Total Response Rate and Responder Counts Used in the Network Meta-Analysis.

Study	Intervention	Comparator	Original response definition / extraction rule	Threshold category	Intervention responders, n/N	Comparator responders, n/N
Wu 2011	MA + MOX	WM	Cured (4) + markedly effective (17) vs cured (1) + markedly effective (15); PASI ≥70%.	PASI ≥70%	21/30	16/30
Jie 2015	MA	WM	Cured (0) + markedly effective (22) vs cured (0) + markedly effective (13); PASI ≥70%.	PASI ≥70%	22/40	13/39
Zhang Cuixia 2016	FN + UR	UR	Cured (27) + markedly effective (17) vs cured (18) + markedly effective (14); PASI ≥60%.	PASI ≥60%	44/50	32/45
Zhang Chen 2016	FN + CHM	WM	Cured (28) + markedly effective (5) vs cured (19) + markedly effective (4); PASI ≥60%.	PASI ≥60%	33/38	23/38
Wei 2017	MA + CHM	WM	Cured (6) + markedly effective (14) vs cured (3) + markedly effective (13); PASI ≥60%.	PASI ≥60%	20/27	16/28
Deng 2017	MOX	WM	Markedly effective cases (PASI ≥70%); no cured cases in either group.	PASI ≥70%	4/30	0/30
Luo 2017	MOX + UR	UR	Cured + markedly effective (overall marked-response rate 90% vs 75%); PASI ≥70%.	PASI ≥70%	18/20	15/20
Hu 2018	FN + CHM	WM	Cured (21) + markedly effective (32) vs cured (12) + markedly effective (28); PASI ≥60%.	PASI ≥60%	53/60	40/57
Lv 2019	FN + MA	MA	Cured (5) + markedly effective (31) vs cured (2) + markedly effective (20); PASI ≥70%.	PASI ≥70%	36/40	22/40
Wang 2019	FN + CHM	WM	Cured (22) + markedly effective (38); score improvement ≥55%.	Score improvement ≥55%	60/67	28/66
Huang 2020	MA + MOX	UR	Cured (26) + improved (11; improvement defined as PASI 60%-95%); PASI ≥60%.	PASI ≥60%	37/40	22/40

Study	Intervention	Comparator	Original response definition / extraction rule	Threshold category	Intervention responders, n/N	Comparator responders, n/N
Tong 2020	FN + UR	UR	At week 5: cured (17) + markedly effective (24) vs cured (10) + markedly effective (16); PASI ≥60%.	PASI ≥60%	41/48	26/48
Chen 2021	MOX	WM	Overall skin lesions: cured (16) + markedly effective (10); PASI ≥75%.	PASI ≥75%	26/38	18/36
Meng 2021	FN + WM	WM	Cured (9) + markedly effective (23) vs cured (4) + markedly effective (16); PASI ≥60%.	PASI ≥60%	32/45	20/45
Jiang 2022	MA + UR	UR	Cured (6) + markedly effective (11) vs cured (1) + markedly effective (9); PASI ≥60%.	PASI ≥60%	17/31	10/32
Xiao 2022a	FN	WM	Cured + markedly effective; PASI ≥60%.	PASI ≥60%	28/31	11/31
Yin 2022	FN + MA	WM	cured (16) + markedly effective (14) vs cured (6) + markedly effective (13); PASI ≥60%.	PASI ≥60%	21/35	19/35
Zhou 2022	MA + UR	UR	Cured + markedly effective; PASI ≥60%.	PASI ≥60%	19/32	12/33
Xiao 2022b	FN	WM	Cured + markedly effective; PASI ≥60%.	PASI ≥60%	30/40	17/40
Tian 2023	FN + CHM	WM	Controlled (18) + markedly effective (23); TCM syndrome score reduction ≥65%.	TCM syndrome score reduction ≥65%	41/56	25/56
Tong Dandan 2023	FN + CHM	WM	Cured (10) + markedly effective (26) vs cured (7) + markedly effective (11); PASI ≥60%.	PASI ≥60%	36/48	18/49
Wang 2023	MWA + CHM	WM	Cured (18) + markedly effective (30) vs cured (8) + markedly effective (28); PASI ≥60%.	PASI ≥60%	48/62	36/62
Yuan 2023	MA + CHM	WM	Markedly effective cases (PASI >60%); “effective” category defined as PASI 30%-60%.	PASI >60%	22/43	18/43
Cui 2024	MA + WM	WM	Cured (9) + markedly effective (27) vs cured (3) + markedly effective (18); PASI ≥60%.	PASI ≥60%	36/60	21/60
Qi 2024	MA + UR	UR	Cured (22) + markedly effective (24) vs cured (19) + markedly	PASI ≥60%	46/65	42/65

Study	Intervention	Comparator	Original response definition / extraction rule	Threshold category	Intervention responders, n/N	Comparator responders, n/N
			effective (23); PASI ≥60%.			
Wang Juan 2024	FN + CHM	WM	Cured (8) + effective (20); skin-lesion clearance ≥50%.	Skin-lesion clearance ≥50%	28/30	21/30
Yang 2024	FN + CHM	WM	Cured (8) + markedly effective (14) vs cured (6) + markedly effective (6); PASI ≥60%.	PASI ≥60%	22/28	12/30
Zuo 2024	MWA + CHM	WM	Cured (13) + markedly effective (19) vs cured (10) + markedly effective (13); PASI ≥60%.	PASI ≥60%	32/40	23/40
Huang 2025	FN + CHM	WM	Cured (23) + markedly effective (19) vs cured (12) + markedly effective (16); PASI ≥60%.	PASI ≥60%	42/50	28/50
Li 2025	FN	WM	Cured (17) + markedly effective (9) vs cured (11) + markedly effective (9); PASI ≥60%.	PASI ≥60%	26/30	20/30
Wang Jiajia 2025	FN + CHM	WM	Cured (2) + markedly effective (8) vs cured (1) + markedly effective (6); PASI ≥60%.	PASI ≥60%	10/20	7/19

Abbreviations: CHM, Chinese herbal medicine; FN, fire-needle acupuncture; MA, manual acupuncture; MOX, moxibustion; MWA, microwave acupuncture; PASI, Psoriasis Area and Severity Index; TCM, traditional Chinese medicine; UR, ultraviolet radiation/phototherapy; WM, Western medicine.

Notes: Definitions of total response rate varied across the included studies. This table presents the original response criteria recorded in the extraction sheet and the corresponding responder counts used in the network meta-analysis. Han 2014 and Gao 2025 did not provide extractable dichotomous response data and were not included in the total response rate network.

Supplementary Section S3: Variables extracted from the included randomized controlled trials

Table S3.1. Variables extracted from the included randomized controlled trials.

Data category	List of variables
Study	Primary author, year of publication, study design, treatment duration, sample size in each group
Patients	Age, disease duration, psoriasis type/stage
Interventions	Type of acupuncture, treatment frequency, combined therapy, comparator intervention
Efficacy outcomes	PASI score, total response rate, DLQI, pruritus score, TNF-α, VEGF
Adverse events	Various adverse events reported in included trials

Abbreviations: DLQI, Dermatology Life Quality Index; PASI, Psoriasis Area and Severity Index; VEGF, vascular endothelial growth factor

Supplementary Section S4: Risk of bias of randomized clinical trials

Figure S1.1. Overall risk of bias presented as percentage of each risk-of-bias item across all included studies. Green = Low risk, yellow = Some concerns, red = High risk.

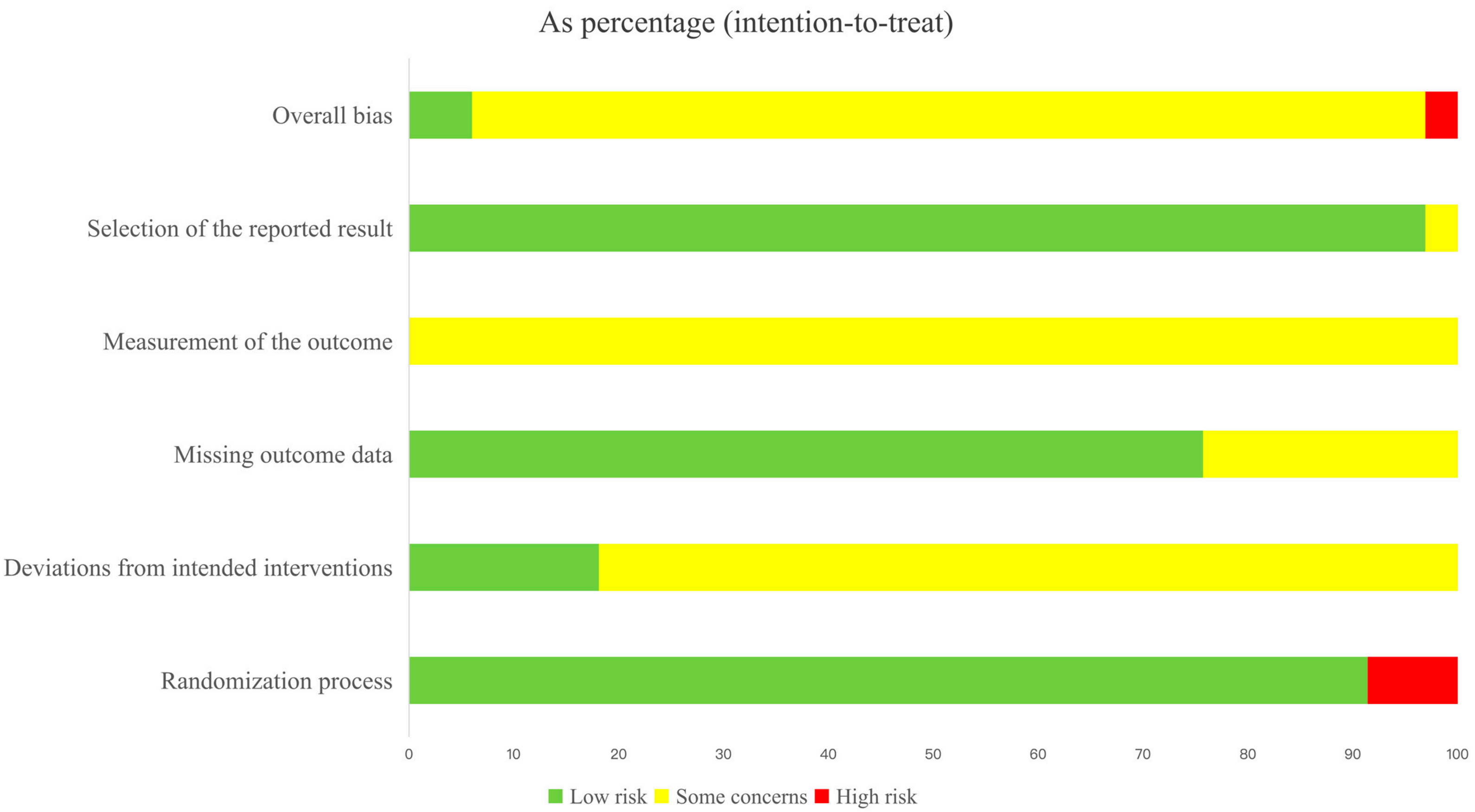

Table S4.1. Study-level risk-of-bias assessment using the Cochrane Risk of Bias 2 (RoB 2) tool for randomized controlled trials.

Unique ID	Randomization process	Deviations from intended interventions	Missing outcome data	Measurement of the outcome	Selection of the reported result	Overall
**Wu Jiaping 2011**	Low	Some concerns	Low	Some concerns	Low	Some concerns
**Han Xuan 2014**	Low	Some concerns	Low	Some concerns	Low	Some concerns
**Jie Si 2015**	Low	Low	Low	Some concerns	Low	Some concerns
**Zhang Cuixia 2016**	Low	Some concerns	Low	Some concerns	Low	Some concerns
**Zhang Chen 2016**	Low	Some concerns	Low	Some concerns	Low	Some concerns
**Deng Guiyan 2017**	Low	Low	Low	Some concerns	Low	Some concerns
**Luo Lingang 2017**	Low	Some concerns	Low	Some concerns	Low	Some concerns
**Wei Fuqiao 2017**	Low	Some concerns	Some concerns	Some concerns	Low	Some concerns
**Hu Suye 2018**	Low	Low	Some concerns	Some concerns	Low	Some concerns
**Lv Shiqi 2019**	Low	Low	Some concerns	Some concerns	Low	Some concerns
**Wang Bei 2019**	Low	Some concerns	Low	Some concerns	Low	Some concerns
**Huang Mei 2020**	Low	Some concerns	Low	Some concerns	Low	Some concerns
**Tong Li 2020**	Low	Some concerns	Low	Some concerns	Low	Some concerns
**Chen Zhaoxia 2021**	Low	Low	Some concerns	Some concerns	Low	Some concerns
**Meng Huijuan 2021**	High	Some concerns	Some concerns	Some concerns	Some concerns	High
**Jiang Yi 2022**	Low	Some concerns	Some concerns	Some concerns	Low	Some concerns
**Xiao Xue 2022a**	Low	Some concerns	Low	Some concerns	Low	Some concerns
**Yin Wenhao 2022**	Low	Some concerns	Low	Some concerns	Low	Some concerns
**Zhou Qiaoying 2022**	Low	Some concerns	Low	Some concerns	Low	Low
**Xiao Xue 2022b**	Low	Some concerns	Low	Some concerns	Low	Some concerns
**Tian Xinke 2023**	Low	Some concerns	Low	Some concerns	Low	Some concerns
**Tong Dandan 2023**	Low	Some concerns	Some concerns	Some concerns	Low	Some concerns
**Wang Le 2023**	Low	Some concerns	Low	Some concerns	Low	Some concerns
**Yuan Hang 2023**	Low	Some concerns	Low	Some concerns	Low	Some concerns
**Cui Yu 2024**	Low	Some concerns	Low	Some concerns	Low	Some concerns
**Qi Zhifeng 2024**	Low	Some concerns	Low	Some concerns	Low	Some concerns
**Wang Juan 2024**	Low	Some concerns	Low	Some concerns	Low	Some concerns
**Yang Yating 2024**	Low	Some concerns	Some concerns	Some concerns	Low	Some concerns
**Zuo Xiaolin 2024**	Low	Low	Low	Some concerns	Low	Some concerns
**Gao Dengxiong 2025**	Low	Some concerns	Low	Some concerns	Low	Some concerns
**Huang Dongming 2025**	Low	Some concerns	Low	Some concerns	Low	Low
**Li Jingjing 2025**	Low	Some concerns	Low	Some concerns	Low	Some concerns
**Wang Jiajia 2025**	Low	Some concerns	Low	Some concerns	Low	Some concerns

Supplementary Section S5: Evaluation of inconsistency and heterogeneity

Table S5.1. Global consistency.

Clinical outcome	Chi square	P value	τ^2 network	I^2
PASI	0.38	0.5381	1.46	93.83%
Total response rate	4.28	0.0786	0.02	43.68%
DLQI	4.77	0.0589	0.14	61.87%
Pruritus score	58.64	0.0000	0.28	81.07%
TNF-α	17.87	0.0000	0.00	0
VEGF	4.88	0.1271	0.17	76.14%
Adverse events	0.14	0.7056	0.00	0

Table S5.2. Node-splitting analysis for PASI: comparison of direct and indirect estimates.

Comparison	Direct		Indirect		Difference			τ² pairwise
	Coef.	Std. Err.	Coef.	Std. Err.	Coef.	Std. Err.	P>|z|	
FN v.s. WM	1.790723	0.7200233	1.384558	18.26692	0.4061648	18.2811	0.982	1.46
FN+CHM v.s. WM	1.742509	0.4148127	1.837719	66.68648	-0.0952105	66.68791	0.999	1.46
FN+MA v.s. MA	1.654328	1.259767	0.3134289	1.776684	1.340899	2.177985	0.538	1.52
FN+MA v.s. WM	1.018553	1.258534	2.359395	1.777333	-1.340842	2.177804	0.538	1.52
FN+UR v.s. UR	3.664317	0.8950956	1.523516	141.4854	2.140801	141.4887	0.988	1.46
FN+WM v.s. WM	0.9030284	1.229903	2.677235	200.0104	-1.774207	200.0144	0.993	1.46
MA v.s. WM	0.7051036	1.254114	-0.6352525	1.780459	1.340356	2.177807	0.538	1.52
MA+CHM v.s. WM	1.788425	0.7169177	1.791759	115.4834	-0.0033334	115.4859	1	1.46
MA+MOX v.s. UR	1.48567	1.236254	0.9417193	37.17187	0.5439507	37.19236	0.988	1.46
MA+MOX v.s. WM	0.6797063	1.238754	1.29263	34.84261	-0.6129239	34.86469	0.986	1.46
MA+UR v.s. UR	1.425683	0.7136988	3.766186	115.5544	-2.340504	115.5568	0.984	1.46
MA+WM v.s. WM	2.440924	1.233943	1.139193	200.003	1.301731	200.0071	0.995	1.46
MOX v.s. WM	0.5101228	0.8742048	3.070326	141.4532	-2.560203	141.4562	0.986	1.46
MOX+UR v.s. UR	1.589369	0.8842185	3.601422	141.4676	-2.012053	141.4707	0.989	1.46
MWA+CHM v.s. WM	1.105996	0.8692294	2.47433	141.4448	-1.368334	141.4476	0.992	1.46

Table S5.3. Node-splitting analysis for total response rate: comparison of direct and indirect estimates.

Comparison	Direct		Indirect		Difference			τ² pairwise
	Coef.	Std. Err.	Coef.	Std. Err.	Coef.	Std. Err.	P>|z|	
FN v.s. WM	-0.5176721	0.1444872	-0.3669613	18.87399	-0.1507108	18.87453	0.994	0.02
FN+CHM v.s. WM	-0.447097	0.0765371	-0.5882375	66.72678	0.1411405	66.72687	0.998	0.02
FN+MA v.s. MA	-0.4924765	0.2011685	0.2382251	0.3118893	-0.7307016	0.3711384	0.079	0.02
FN+MA v.s. WM	-0.1000836	0.2456504	-0.8307852	0.2782069	0.7307016	0.371138	0.079	0.02
FN+UR v.s. UR	-0.3200101	0.1406468	-1.211168	141.2328	0.891158	141.2329	0.995	0.02
FN+WM v.s. WM	-0.4700036	0.2456967	-0.5652354	199.917	0.0952318	199.9174	1	0.02
MA v.s. WM	-0.3383111	0.1921747	0.3923827	0.3175083	-0.7306938	0.3711373	0.079	0.02
MA+CHM v.s. WM	-0.2099797	0.1942453	-0.8253467	141.3377	0.6153669	141.3381	0.997	0.02
MA+MOX v.s. UR	-0.5198756	0.2145769	-0.3491135	39.98064	-0.1707622	39.98119	0.997	0.02
MA+MOX v.s. WM	-0.2719341	0.2588707	-0.4530017	37.11426	0.1810677	37.1152	0.996	0.02
MA+UR v.s. UR	-0.2708657	0.1530818	-1.261202	115.2502	0.9903363	115.2504	0.993	0.02
MA+WM v.s. WM	-0.5389965	0.2561735	-0.4964082	200.0249	-0.0425883	200.0253	1	0.02
MOX v.s. WM	-0.3136576	0.2519622	-0.7216167	200.0835	0.4079591	200.0839	0.998	0.02
MOX+UR v.s. UR	-2.197225	1.466474	0.6660807	200.0019	-2.863305	200.0179	0.989	0.02
MWA+CHM v.s. WM	-0.3069413	0.1478276	-0.7284282	141.5097	0.4214868	141.5098	0.998	0.02

Table S5.4 Node-splitting analysis for adverse events: comparison of direct and indirect estimates.

Comparison	Direct		Indirect		Difference			τ² pairwise
	Coef.	Std. Err.	Coef.	Std. Err.	Coef.	Std. Err.	P>|z|	
FN+CHM v.s. WM	0.9482564	0.6568183	2.365413	27.74588	-1.417156	27.75366	0.959	6.25E-14
FN+MA v.s. MA	-7.86E-12	2.012308	1.947398	200.057	-1.947398	200.0874	0.992	9.00E-18
FN+UR v.s. UR	1.210164	0.3922849	-7.732237	141.3588	8.9424	141.3604	0.95	1.12E-12
FN+WM v.s. WM	-0.7407752	0.8931103	2.638985	200.045	-3.37976	200.051	0.987	7.18E-16
MA v.s. WM	-0.0249224	2.012382	1.143998	89.46136	-1.168921	89.49305	0.99	1.66E-19
MA+CHM v.s. WM	-0.1408558	0.3790137	2.039238	115.4627	-2.180094	115.4645	0.985	1.92E-17
MA+MOX v.s. UR	1.039741	0.5289849	5.214742	48.52396	-4.175001	48.5265	0.931	1.90E-14
MA+MOX v.s. WM	5.252728	1.486122	1.052042	43.64519	4.200686	43.67288	0.923	1.64E-13
MA+UR v.s. UR	-0.1712451	0.5862715	-6.347308	199.8245	6.176063	199.8271	0.975	1.43E-19
MOX v.s. WM	2.085023	1.530209	-0.1870207	200.004	2.272044	200.0216	0.991	1.82E-18
MOX+UR v.s. UR	0.2876821	0.76103	-6.806502	200.1375	7.094184	200.1419	0.972	1.48E-17

Supplementary Section S6: Network maps and forest of secondary outcomes plots

Figure S2.1: Network map and forest plot for the Total response rate, with network effect estimates relative to WM. Node size is proportional to the total number of participants receiving each intervention, and line thickness is proportional to the number of studies contributing to each direct comparison.

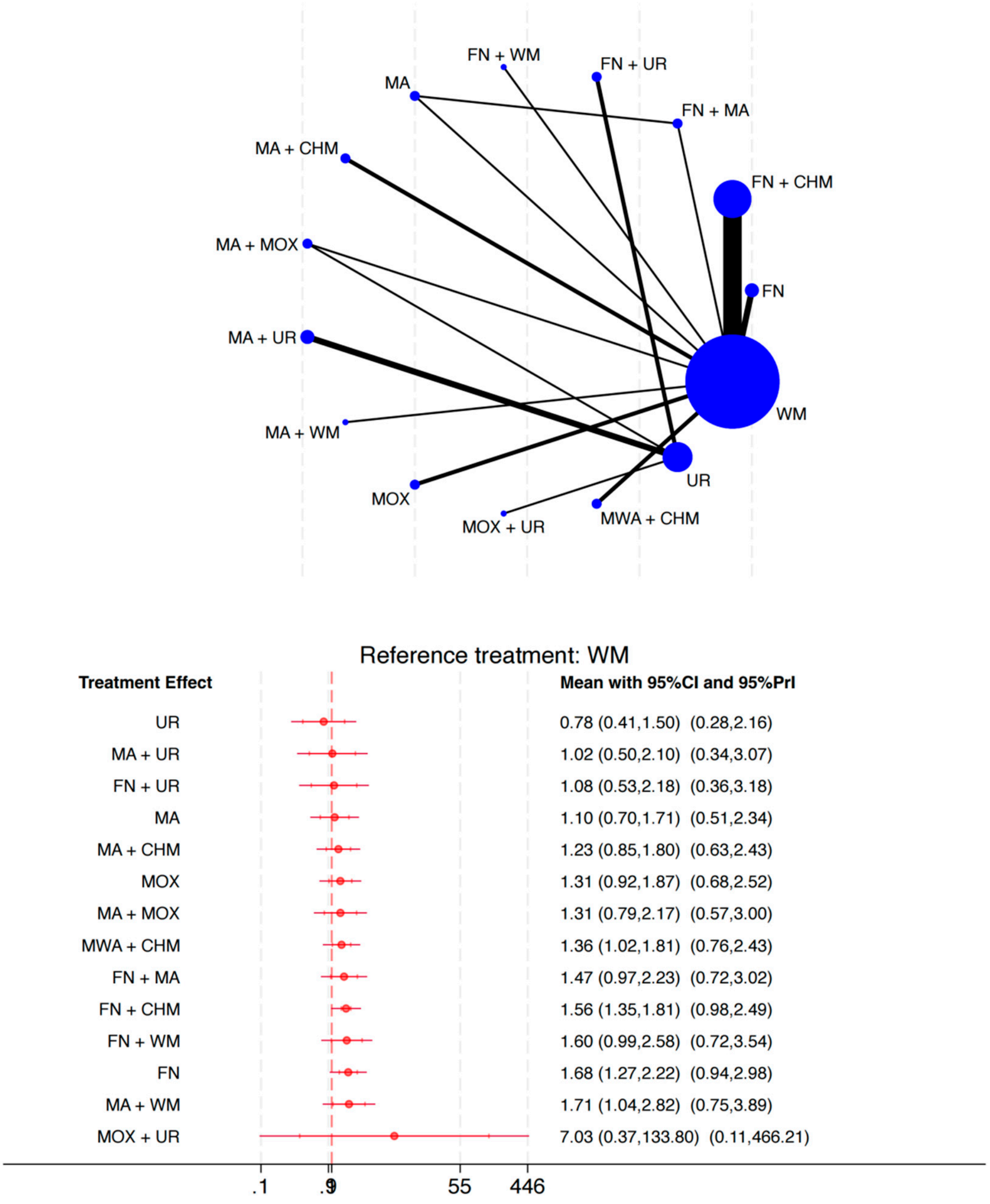

Figure S2.2: Network map and forest plot for the DLQI, with network effect estimates relative to WM. Node size is proportional to the total number of participants receiving each intervention, and line thickness is proportional to the number of studies contributing to each direct comparison.

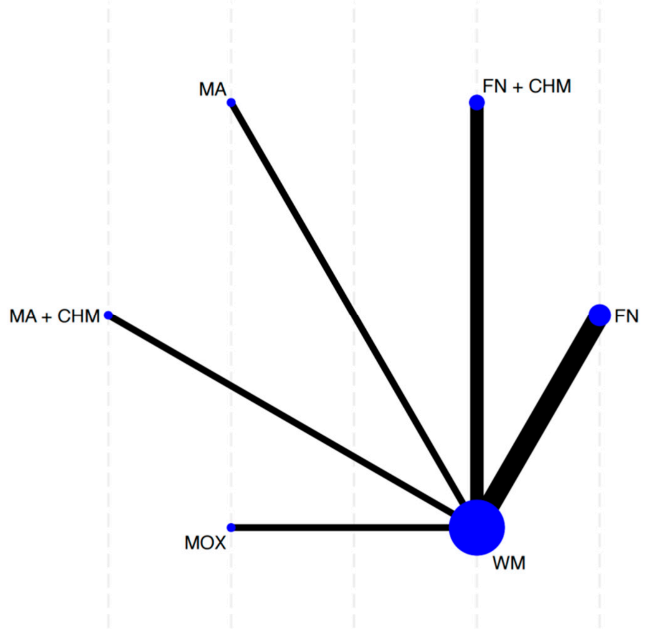

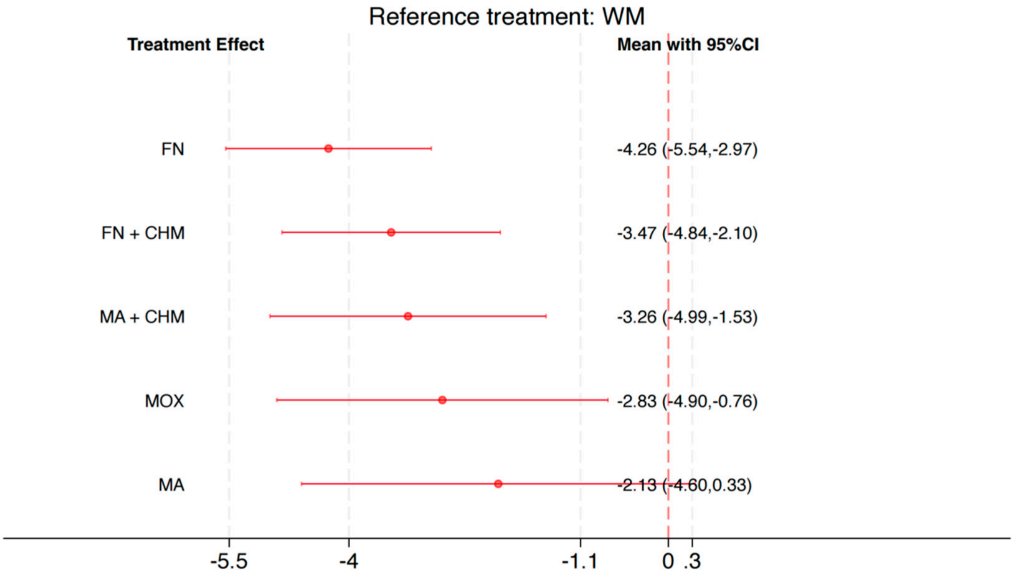

Figure S2.3: Network map and forest plot for the Pruritus score, with network effect estimates relative to WM. Node size is proportional to the total number of participants receiving each intervention, and line thickness is proportional to the number of studies contributing to each direct comparison.

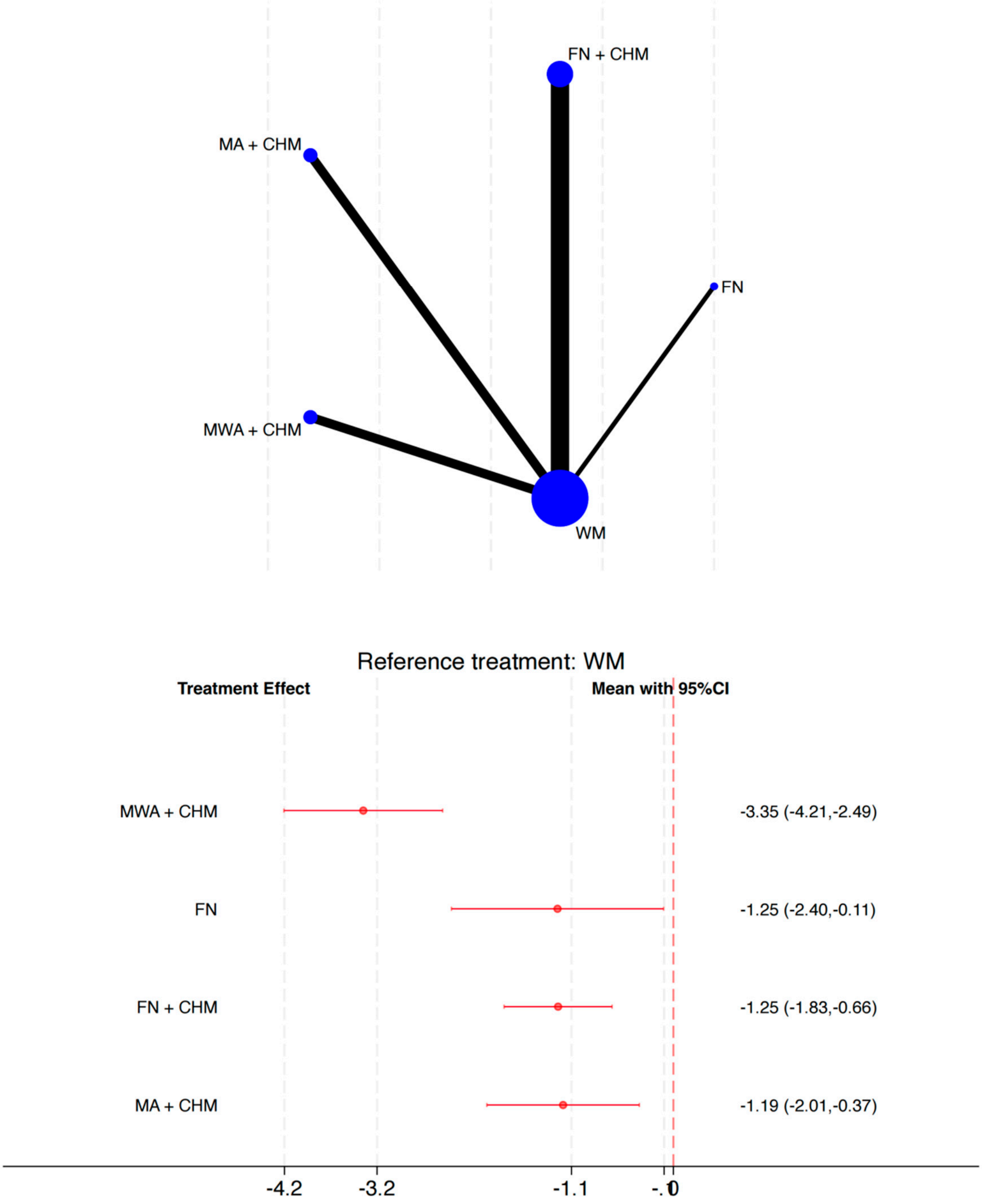

Figure S2.4: Network map and forest plot for the TNF-α, with network effect estimates relative to WM. Node size is proportional to the total number of participants receiving each intervention, and line thickness is proportional to the number of studies contributing to each direct comparison.

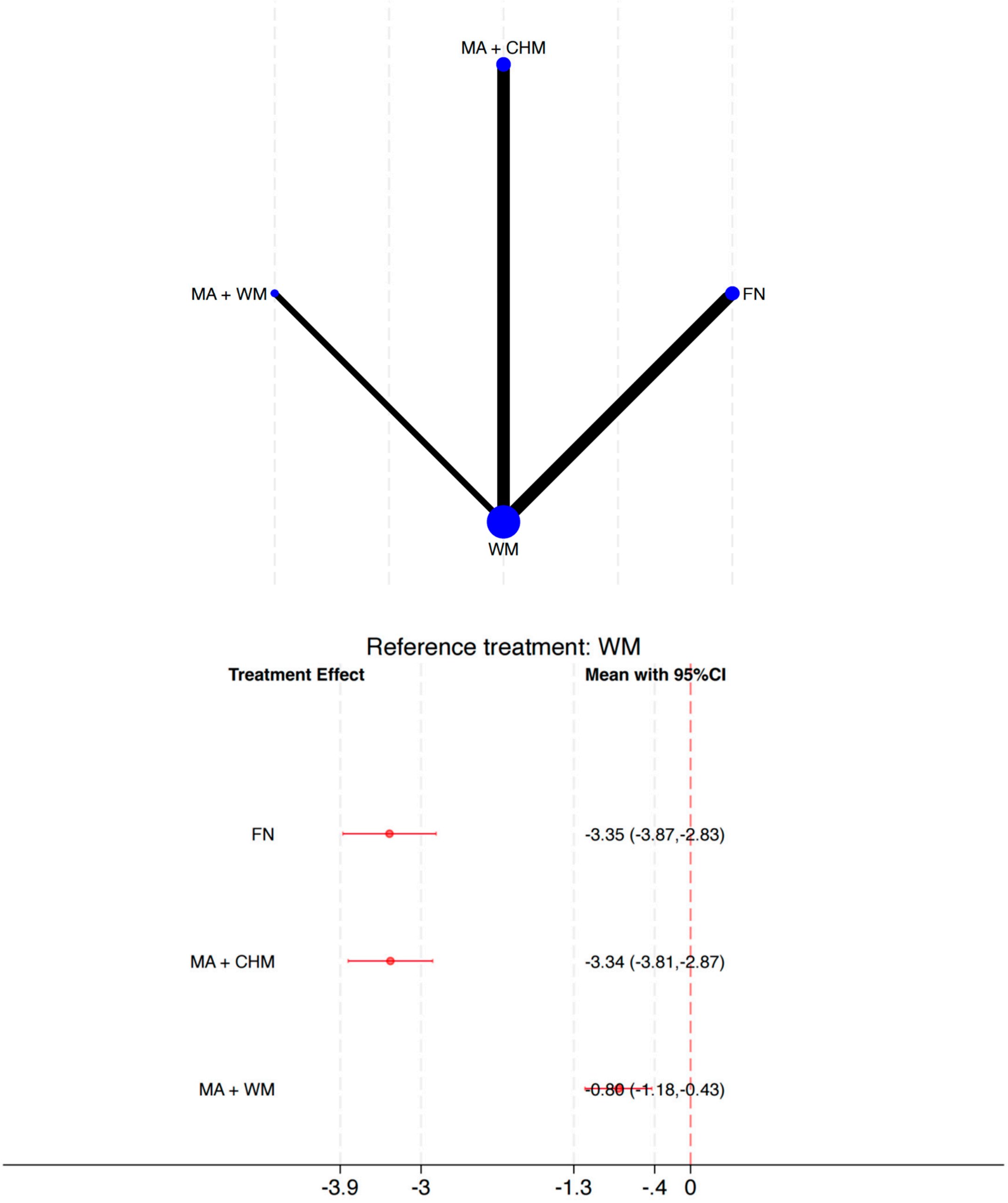

Figure S2.5: Network map and forest plot for the VEGF, with network effect estimates relative to WM. Node size is proportional to the total number of participants receiving each intervention, and line thickness is proportional to the number of studies contributing to

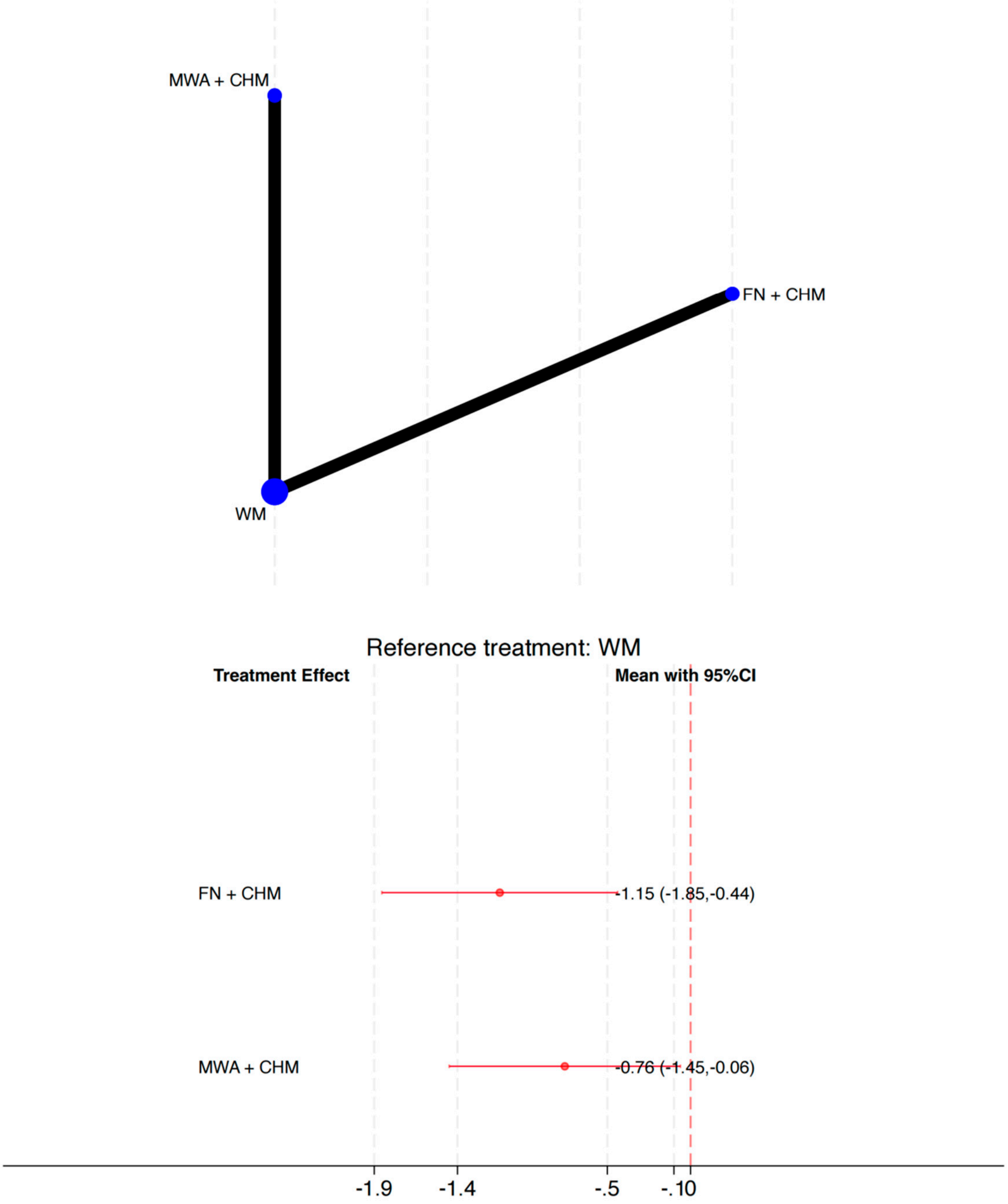

each direct comparison.

Figure S2.6: Network map and forest plot for the Adverse events, with network effect estimates relative to WM. Node size is proportional to the total number of participants receiving each intervention, and line thickness is proportional to the number of studies contributing to each direct comparison

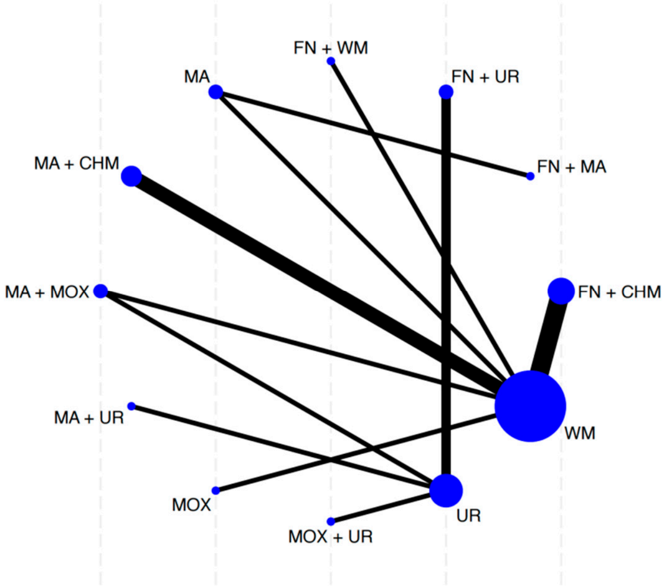

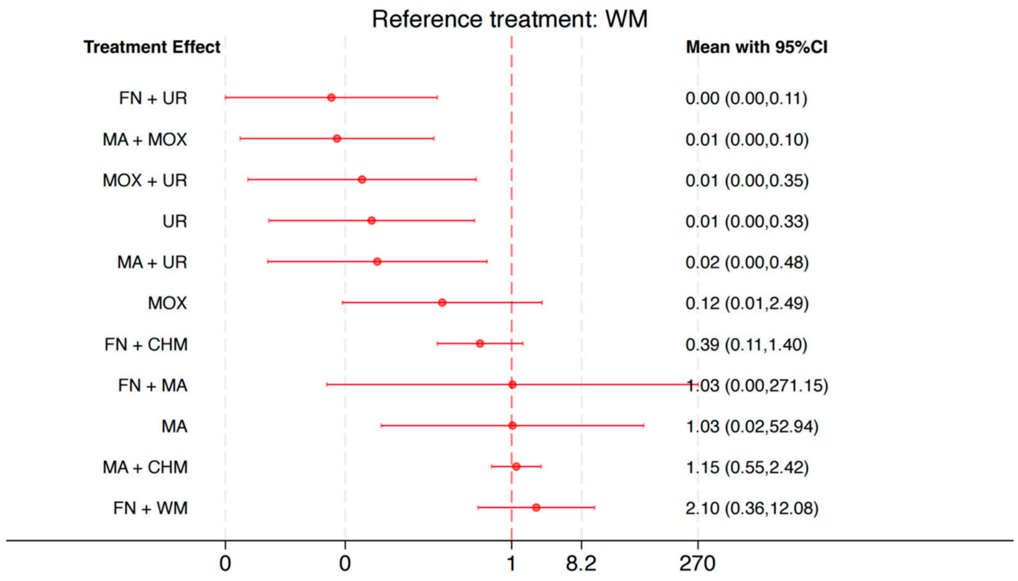

Supplementary Section S7: SUCRA and cumulative ranking plots

Figure S3.1. Cumulative ranking curves for treatments based on PASI.

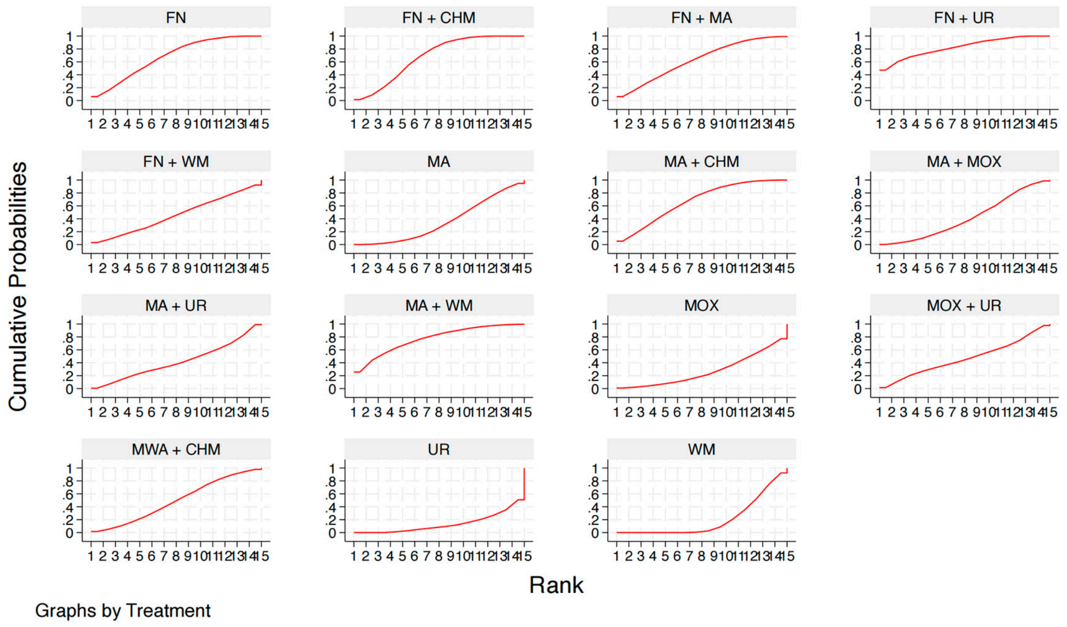

Table S6.1. SUCRA rankings of treatments for PASI.

Treatment	SUCRA	PrBest	MeanRank
**FN**	68.7	5.1	5.4
**FN + CHM**	67.7	1.9	5.5
**FN + MA**	58.7	4.9	6.8
**FN + UR**	83.4	47.3	3.3
**FN + WM**	46	4.4	8.6
**MA**	29.5	0.4	10.9
**MA + CHM**	67.8	5.4	5.5
**MA + MOX**	41.8	0.2	9.2
**MA + UR**	43.4	0.5	8.9
**MA + WM**	77.7	26	4.1
**MOX**	35.5	0.3	10
**MOX + UR**	46.1	1.6	8.5
**MWA + CHM**	50.1	1.9	8
**UR**	13.7	0	13.1
**WM**	20	0	12.2

Abbreviations: PASI, Psoriasis Area and Severity Index; SUCRA, surface under the cumulative ranking curve.

Figure S3.2. Cumulative ranking curves for treatments based on total response rate.

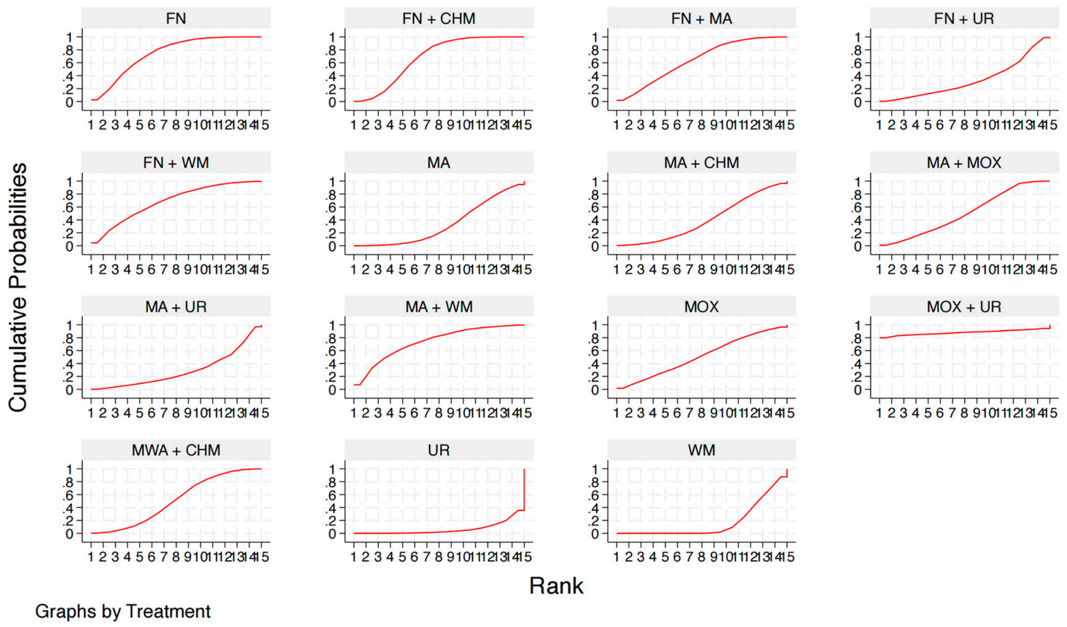

Table S6.2. SUCRA rankings of treatments for total response rate.

Treatment	SUCRA	PrBest	MeanRank
**FN**	75.5	4.1	4.4
**FN + CHM**	70.3	0.7	5.2
**FN + MA**	61.4	1.8	6.4
**FN + UR**	34	0.3	10.2
**FN + WM**	69.7	4.3	5.2
**MA**	29.2	0.1	10.9
**MA + CHM**	41.5	0.3	9.2
**MA + MOX**	51.5	0.6	7.8
**MA + UR**	30.1	0.1	10.8
**MA + WM**	74	7.3	4.6
**MOX**	47.7	0.3	8.3
**MOX + UR**	87.7	79.9	2.7
**MWA + CHM**	52.2	0.2	7.7
**UR**	6.9	0	14
**WM**	18.2	0	12.5

Abbreviation: SUCRA, surface under the cumulative ranking curve.

Figure S3.3. Cumulative ranking curves for treatments based on DLQI.

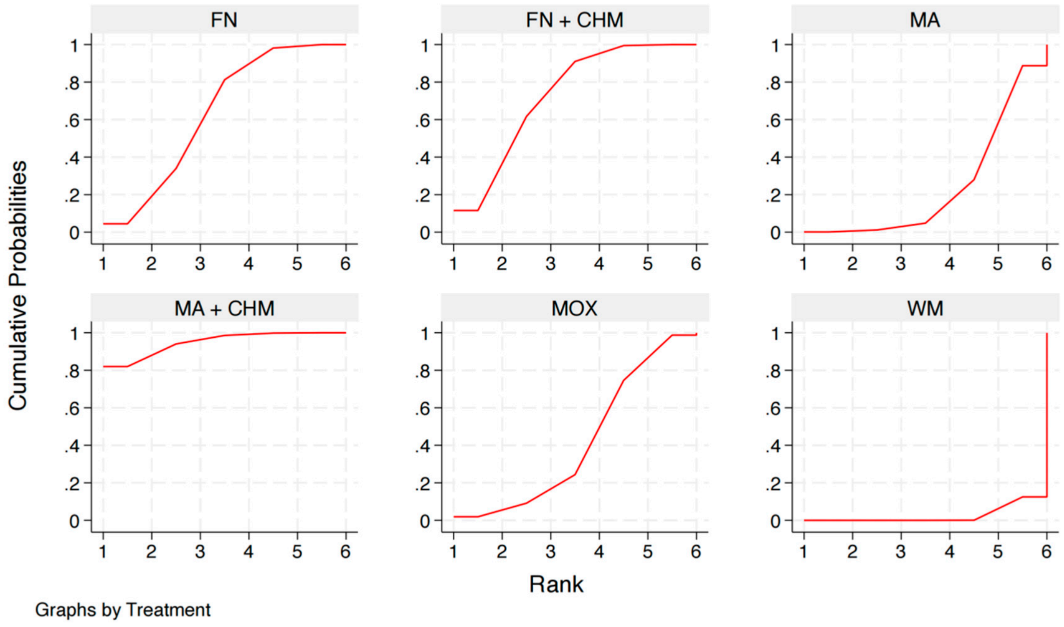

Table S6.3. SUCRA rankings of treatments for DLQI.

Treatment	SUCRA	PrBest	MeanRank
**MA+CHM**	94.9	82	1.3
**FN+CHM**	72.7	11.6	2.4
**FN**	63.6	4.4	2.8
**MOX**	41.7	1.9	3.9
**MA**	24.5	0.1	4.8
**WM**	2.5	0	5.9

Abbreviations: DLQI, Dermatology Life Quality Index; SUCRA, surface under the cumulative ranking curve.

Figure S3.4. Cumulative ranking curves for treatments based on pruritus score.

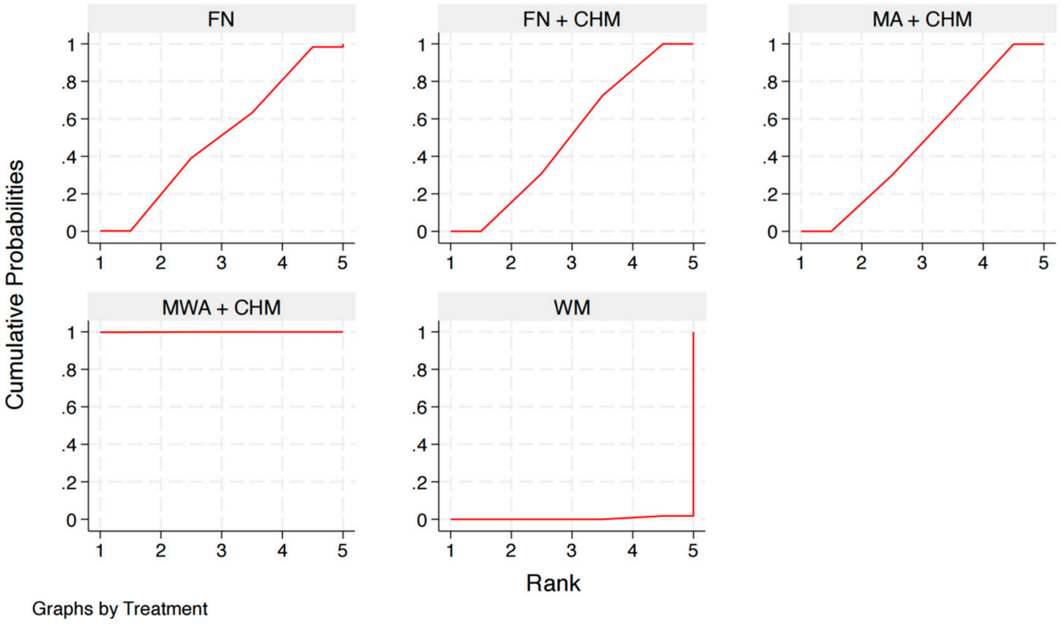

Table S6.4. SUCRA rankings of treatments for pruritus score.

Treatment	SUCRA	PrBest	MeanRank
**MWA+CHM**	99.9	99.8	1
**FN+CHM**	50.8	0	3
**FN**	50.2	0.2	3
**MA+CHM**	48.6	0	3.1
**WM**	0.4	0	5

Abbreviation: SUCRA, surface under the cumulative ranking curve.

Figure S3.5. Cumulative ranking curves for treatments based on TNF-α.

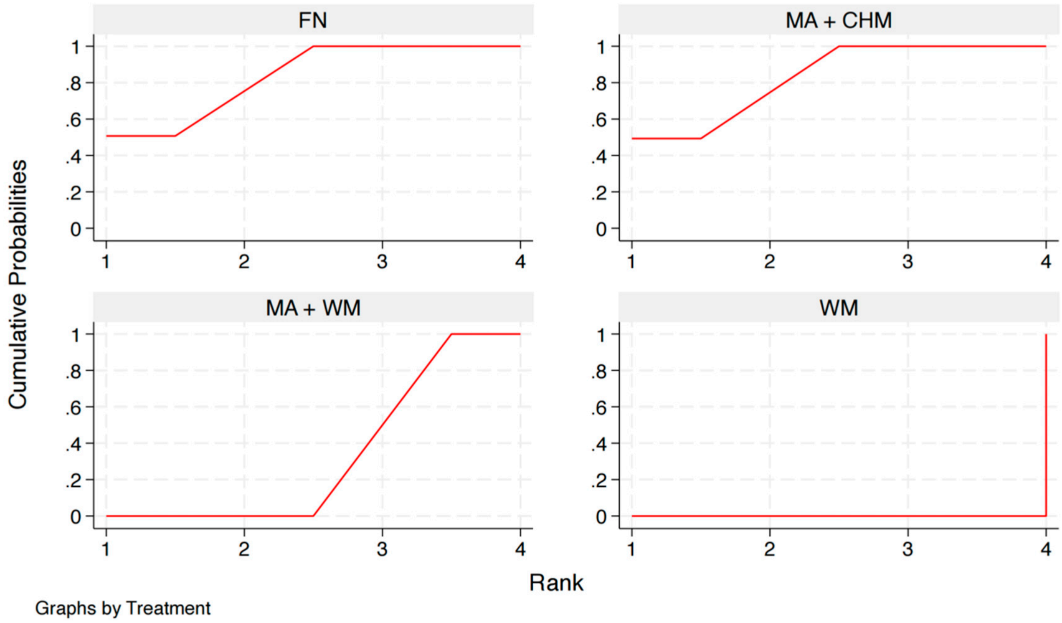

Table S6.5. SUCRA rankings of treatments for TNF-α.

Treatment	SUCRA	PrBest	MeanRank
**FN**	83.6	50.7	1.5
**MA+CHM**	83.1	49.3	1.5
**MA+WM**	33.3	0	3
**WM**	0	0	4

Abbreviations: TNF-α, tumor necrosis factor-alpha; SUCRA, surface under the cumulative ranking curve.

Figure S3.6. Cumulative ranking curves for treatments based on VEGF.

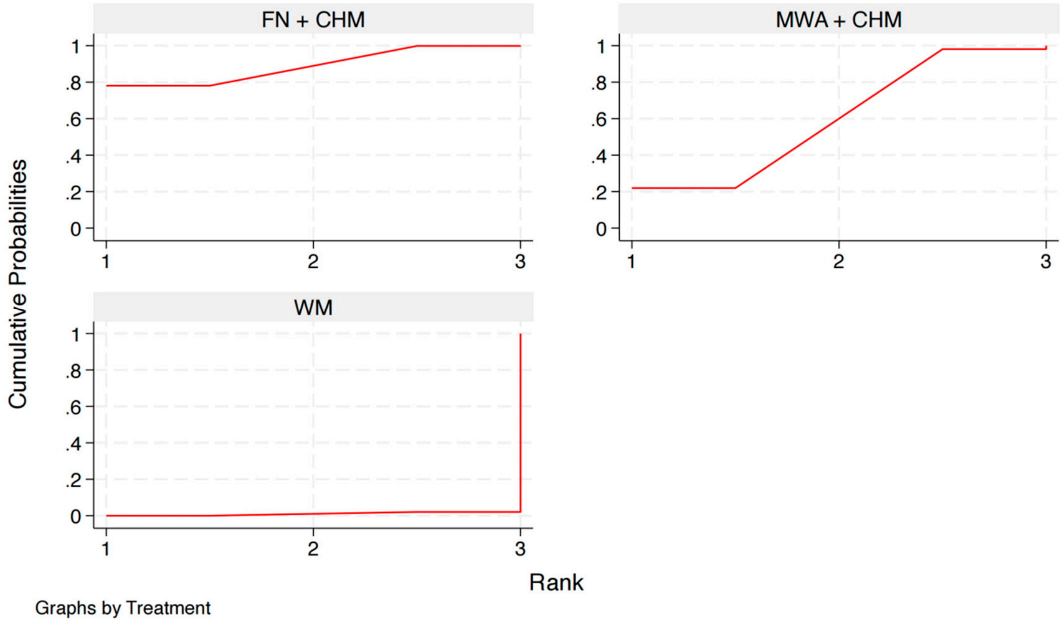

Table S6.6. SUCRA rankings of treatments for VEGF.

Treatment	SUCRA	PrBest	MeanRank
**FN+CHM**	89	78.1	1.2
**MWA+CHM**	60	22	1.8
**WM**	1	0	3

Abbreviations: VEGF, vascular endothelial growth factor; SUCRA, surface under the cumulative ranking curve.

Figure S3.7. Cumulative ranking curves for treatments based on adverse events.

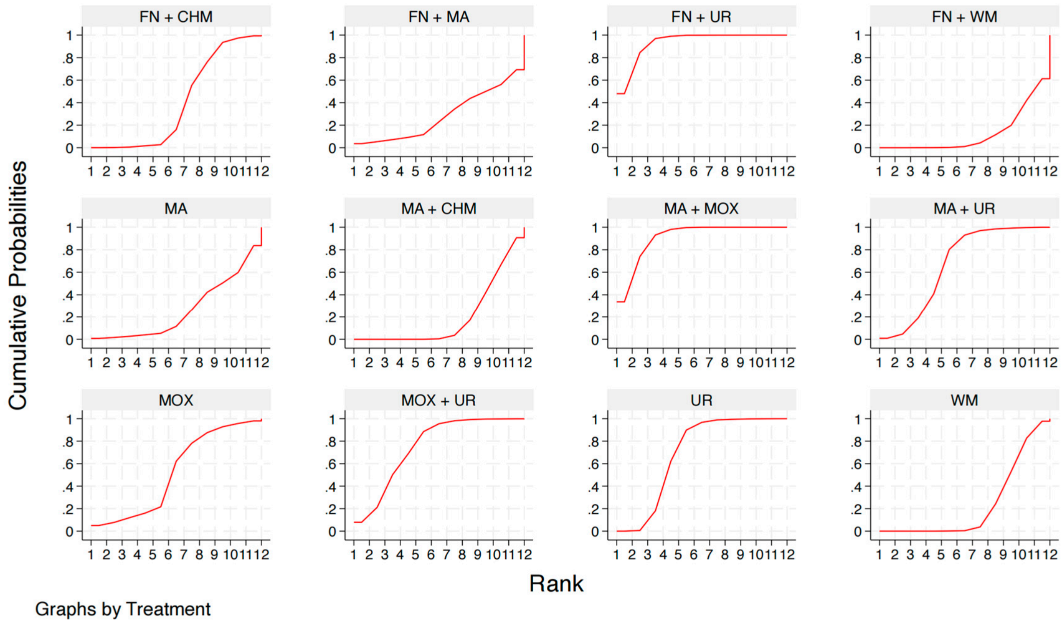

Table S6.7. SUCRA rankings of treatments for adverse events.

Treatment	SUCRA	PrBest	MeanRank
**FN+UR**	93.2	48.3	1.7
**MA+MOX**	90.6	32.2	2
**MOX+UR**	76	8.7	3.6
**UR**	69.1	0	4.4
**MA+UR**	66.5	0.9	4.7
**MOX**	52.4	5.2	6.2
**FN+CHM**	40.6	0.1	7.5
**FN+MA**	28.8	3.9	8.8
**MA**	26.6	0.5	9.1
**WM**	23.9	0	9.4
**MA+CHM**	20.1	0	9.8
**FN+WM**	12.2	0	10.7

Abbreviation: SUCRA, surface under the cumulative ranking curve.

Supplementary Section S8: League tables of network meta-analysis estimates

Table S7.1. PASI.

Each cell presents the effect of the treatment listed in the column relative to that listed in the row. Effect estimates are expressed as standardized mean differences (SMDs) with 95% confidence intervals (CIs). For example, the SMD for PASI between FN and FN+CHM was −0.03 (95% CI −1.64 to 1.59). Because lower PASI scores indicate greater improvement, an SMD <0 favors the treatment in the column, whereas an SMD >0 favors the treatment in the row. Abbreviations: SMD, standardized mean difference; PASI, Psoriasis Area and Severity Index.

FN														
-0.03<br>(-1.64,1.59)	FN+CHM													
-0.30<br>(-2.72,2.11)	-0.28<br>(-2.41,1.86)	FN+MA												
1.12<br>(-2.92,5.17)	1.15<br>(-2.74,5.05)	1.43<br>(-2.86,5.72)	FN+UR											
-0.87<br>(-3.65,1.91)	-0.84<br>(-3.38,1.70)	-0.56<br>(-3.67,2.55)	-1.99<br>(-6.50,2.51)	FN+WM										
-1.51<br>(-3.92,0.90)	-1.48<br>(-3.61,0.65)	-1.21<br>(-3.18,0.77)	-2.63<br>(-6.92,1.65)	-0.64<br>(-3.75,2.47)	MA									
0.02<br>(-1.96,2.00)	0.05<br>(-1.57,1.67)	0.32<br>(-2.10,2.74)	-1.11<br>(-5.16,2.95)	0.89<br>(-1.90,3.67)	1.53<br>(-0.89,3.94)	MA+CHM								
-1.07<br>(-3.85,1.70)	-1.04<br>(-3.58,1.50)	-0.77<br>(-3.88,2.34)	-2.20<br>(-5.17,0.78)	-0.20<br>(-3.61,3.20)	0.44<br>(-2.67,3.55)	-1.09<br>(-3.88,1.70)	MA+MOX							
-1.11<br>(-5.02,2.79)	-1.09<br>(-4.83,2.66)	-0.81<br>(-4.97,3.35)	-2.24<br>(-4.48,0.00)	-0.25<br>(-4.62,4.13)	0.40<br>(-3.76,4.55)	-1.13<br>(-5.05,2.79)	-0.04<br>(-2.82,2.74)	MA+UR						
0.67<br>(-2.12,3.46)	0.70<br>(-1.85,3.24)	0.97<br>(-2.14,4.09)	-0.45<br>(-4.96,4.06)	1.54<br>(-1.87,4.94)	2.18<br>(-0.94,5.29)	0.65<br>(-2.14,3.44)	1.74<br>(-1.67,5.15)	1.78<br>(-2.60,6.17)	MA+WM					
-1.26<br>(-3.47,0.95)	-1.23<br>(-3.12,0.66)	-0.96<br>(-3.57,1.65)	-2.38<br>(-6.56,1.79)	-0.39<br>(-3.34,2.56)	0.25<br>(-2.36,2.86)	-1.28<br>(-3.49,0.93)	-0.19<br>(-3.14,2.77)	-0.15<br>(-4.18,3.89)	-1.93<br>(-4.89,1.03)	MOX				
-0.95<br>(-4.99,3.08)	-0.92<br>(-4.81,2.96)	-0.65<br>(-4.93,3.63)	-2.07<br>(-4.53,0.39)	-0.08<br>(-4.58,4.41)	0.56<br>(-3.72,4.84)	-0.97<br>(-5.02,3.08)	0.12<br>(-2.84,3.08)	0.16<br>(-2.06,2.39)	-1.62<br>(-6.12,2.88)	0.31<br>(-3.86,4.48)	MOX+UR			
-0.67<br>(-2.87,1.53)	-0.64<br>(-2.52,1.25)	-0.36<br>(-2.97,2.24)	-1.79<br>(-5.96,2.38)	0.20<br>(-2.74,3.15)	0.84<br>(-1.76,3.45)	-0.68<br>(-2.89,1.52)	0.41<br>(-2.54,3.36)	0.45<br>(-3.59,4.48)	-1.33<br>(-4.29,1.62)	0.60<br>(-1.82,3.01)	0.29<br>(-3.88,4.45)	MWA+CHM		
-2.54<br>(-6.19,1.11)	-2.51<br>(-5.99,0.97)	-2.24<br>(-6.15,1.68)	-3.66<br>(-5.42,-1.91)	-1.67<br>(-5.83,2.48)	-1.03<br>(-4.95,2.88)	-2.56<br>(-6.22,1.10)	-1.47<br>(-3.87,0.94)	-1.43<br>(-2.82,-0.03)	-3.21<br>(-7.37,0.95)	-1.28<br>(-5.07,2.51)	-1.59<br>(-3.32,0.14)	-1.88<br>(-5.66,1.91)	UR	
-1.77<br>(-3.17,-0.37)	-1.74<br>(-2.55,-0.93)	-1.47<br>(-3.44,0.51)	-2.90<br>(-6.70,0.91)	-0.90<br>(-3.31,1.50)	-0.26<br>(-2.23,1.71)	-1.79<br>(-3.19,-0.39)	-0.70<br>(-3.11,1.71)	-0.66<br>(-4.32,3.00)	-2.44<br>(-4.85,-0.03)	-0.51<br>(-2.22,1.20)	-0.82<br>(-4.62,2.98)	-1.11<br>(-2.81,0.59)	0.77<br>(-2.62,4.15)	WM

Table S7.2. Total response rate.

Each cell presents the effect of the treatment listed in the column relative to that listed in the row. Effect estimates are expressed as risk ratios (RRs) with 95% confidence intervals (CIs). For example, the RR for the total response rate between FN and FN+CHM was 1.07 (95% CI 0.78 to 1.47). Because a higher total response rate indicates greater efficacy, an RR >1 favors the treatment in the column, whereas an RR <1 favors the treatment in the row. Abbreviation: RR, risk ratio.

FN														
1.07 (0.78,1.47)	FN+CHM													
1.14 (0.69,1.88)	1.06 (0.68,1.65)	FN+MA												
1.56 (0.73,3.33)	1.45 (0.71,2.99)	1.37 (0.60,3.10)	FN+UR											
1.05 (0.60,1.82)	0.98 (0.59,1.61)	0.92 (0.49,1.73)	0.67 (0.29,1.58)	FN+WM										
1.53 (0.91,2.58)	1.43 (0.89,2.28)	1.34 (0.92,1.95)	0.98 (0.43,2.26)	1.46 (0.76,2.81)	MA									
1.36 (0.85,2.18)	1.27 (0.84,1.90)	1.19 (0.68,2.09)	0.87 (0.39,1.94)	1.30 (0.71,2.39)	0.89 (0.49,1.60)	MA+CHM								
1.28 (0.72,2.27)	1.19 (0.70,2.01)	1.12 (0.58,2.15)	0.82 (0.50,1.35)	1.22 (0.61,2.44)	0.83 (0.43,1.64)	0.94 (0.50,1.76)	MA+MOX							
1.64 (0.76,3.53)	1.53 (0.74,3.17)	1.44 (0.63,3.29)	1.05 (0.70,1.57)	1.57 (0.66,3.71)	1.07 (0.46,2.49)	1.21 (0.54,2.72)	1.28 (0.77,2.14)	MA+UR						
0.98 (0.55,1.73)	0.91 (0.54,1.53)	0.86 (0.45,1.64)	0.63 (0.26,1.49)	0.93 (0.47,1.86)	0.64 (0.33,1.25)	0.72 (0.38,1.35)	0.77 (0.38,1.56)	0.60 (0.25,1.43)	MA+WM					
1.28 (0.81,2.01)	1.19 (0.81,1.75)	1.12 (0.65,1.94)	0.82 (0.37,1.81)	1.22 (0.67,2.21)	0.84 (0.47,1.48)	0.94 (0.56,1.58)	1.00 (0.54,1.85)	0.78 (0.35,1.74)	1.31 (0.71,2.41)	MOX				
0.24 (0.01,4.60)	0.22 (0.01,4.25)	0.21 (0.01,4.10)	0.15 (0.01,2.74)	0.23 (0.01,4.50)	0.16 (0.01,3.07)	0.18 (0.01,3.42)	0.19 (0.01,3.41)	0.15 (0.01,2.61)	0.24 (0.01,4.84)	0.19 (0.01,3.63)	MOX+UR			
1.23 (0.83,1.84)	1.15 (0.83,1.59)	1.08 (0.65,1.79)	0.79 (0.37,1.70)	1.18 (0.67,2.05)	0.81 (0.47,1.37)	0.91 (0.56,1.46)	0.97 (0.54,1.72)	0.75 (0.35,1.63)	1.26 (0.71,2.24)	0.96 (0.61,1.52)	5.17 (0.27,99.82)	MWA+CHM		
2.15 (1.05,4.37)	2.00 (1.02,3.91)	1.88 (0.87,4.08)	1.38 (1.05,1.81)	2.05 (0.91,4.60)	1.40 (0.64,3.10)	1.58 (0.74,3.36)	1.68 (1.11,2.55)	1.31 (0.97,1.76)	2.19 (0.97,4.99)	1.68 (0.80,3.53)	9.00 (0.51,159.22)	1.74 (0.85,3.55)	UR	
1.68 (1.27,2.22)	1.56 (1.35,1.81)	1.47 (0.97,2.23)	1.08 (0.53,2.18)	1.60 (0.99,2.58)	1.10 (0.70,1.71)	1.23 (0.85,1.80)	1.31 (0.79,2.17)	1.02 (0.50,2.10)	1.71 (1.04,2.82)	1.31 (0.92,1.87)	7.03 (0.37,133.80)	1.36 (1.02,1.81)	0.78 (0.41,1.50)	WM

Table S7.3. DLQI.

Each cell presents the effect of the treatment listed in the column relative to that listed in the row. Effect estimates are expressed as mean differences (MDs) with 95% confidence intervals (CIs). For example, the MD for DLQI between FN and FN+CHM was −0.78 (95% CI −2.62 to 1.05). Because lower DLQI scores indicate better health-related quality of life, an MD <0 favors the treatment in the column, whereas an MD >0 favors the treatment in the row. Abbreviations: MD, mean difference; DLQI, Dermatology Life Quality Index.

FN					
-0.78<br>(-2.62,1.05)	FN+CHM				
-2.13<br>(-4.91,0.65)	-1.34<br>(-4.16,1.48)	MA			
-1.00<br>(-3.15,1.16)	-0.21<br>(-2.41,1.99)	1.13<br>(-1.88,4.14)	MA+CHM		
-1.43<br>(-3.87,1.01)	-0.64<br>(-3.13,1.84)	0.70<br>(-2.52,3.92)	-0.43<br>(-3.13,2.27)	MOX	
-4.26<br>(-5.54,-2.97)	-3.47<br>(-4.84,-2.10)	-2.13<br>(-4.60,0.33)	-3.26<br>(-4.99,-1.53)	-2.83<br>(-4.90,-0.76)	WM

Table S7.4. Pruritus score.

Each cell presents the effect of the treatment listed in the column relative to that listed in the row. Effect estimates are expressed as standardized mean differences (SMDs) with 95% confidence intervals (CIs). For example, the SMD for pruritus score between FN and FN+CHM was −0.01 (95% CI −1.29 to 1.28). Because lower pruritus scores indicate greater symptom improvement, an SMD <0 favors the treatment in the column, whereas an SMD >0 favors the treatment in the row. Abbreviation: SMD, standardized mean difference.

FN				
-0.01<br>(-1.29,1.28)	FN+CHM			
-0.06<br>(-1.47,1.35)	-0.05<br>(-1.06,0.95)	MA+CHM		
2.10<br>(0.67,3.53)	2.10<br>(1.07,3.14)	2.16<br>(0.97,3.35)	MWA+CHM	
-1.25<br>(-2.40,-0.11)	-1.25<br>(-1.83,-0.66)	-1.19<br>(-2.01,-0.37)	-3.35<br>(-4.21,-2.49)	WM

Table S7.5. TNF-α.

Each cell presents the effect of the treatment listed in the column relative to that listed in the row. Effect estimates are expressed as standardized mean differences (SMDs) with 95% confidence intervals (CIs). For example, the SMD for TNF-α between FN and MA+CHM was −0.01 (95% CI −0.71 to 0.69). Because lower TNF-α levels indicate reduced inflammation, an SMD <0 favors the treatment in the column, whereas an SMD >0 favors the treatment in the row. Abbreviations: SMD, standardized mean difference; TNF-α, tumor necrosis factor-alpha.

FN			
-0.01<br>(-0.71,0.69)	MA+CHM		
-2.55<br>(-3.19,-1.91)	-2.54<br>(-3.14,-1.94)	MA+WM	
-3.35<br>(-3.87,-2.83)	-3.34<br>(-3.81,-2.87)	-0.80<br>(-1.18,-0.43)	WM

Table S7.6. VEGF.

Each cell presents the effect of the treatment listed in the column relative to that listed in the row. Effect estimates are expressed as standardized mean differences (SMDs) with 95% confidence intervals (CIs). For example, the SMD for VEGF between FN + CHM and MWA + CHM was -0.49 (95% CI -1.40,0.42). Because lower VEGF levels are considered favorable, an SMD <0 favors the treatment in the column, whereas an SMD >0 favors the treatment in the row. Abbreviations: SMD, standardized mean difference; VEGF, vascular endothelial growth factor.

FN + CHM		
-0.49<br>(-1.40,0.42)	MWA + CHM	
-1.15<br>(-1.85,-0.44)	-0.76<br>(-1.45,-0.06)	WM

Table S7.7. Adverse events.

Each cell presents the effect of the treatment listed in the column relative to that listed in the row. Effect estimates are expressed as odds ratios (ORs) with 95% confidence intervals (CIs). For example, the OR for adverse events between MA+MOX and MA+UR was 0.30 (95% CI 0.06 to 1.40). Because fewer adverse events indicate better safety, an OR <1 favors the treatment in the column, whereas an OR >1 favors the treatment in the row. Abbreviation: OR, odds ratio.

FN+CHM											
0.38 (0.00,115.59)	FN+MA										
87.10 (2.81,2696.08)	230.74 (0.37,141977.96)	FN+UR									
0.18 (0.02,1.62)	0.49 (0.00,169.05)	0.00 (0.00,0.08)	FN+WM								
0.38 (0.01,23.92)	1.00 (0.02,51.62)	0.00 (0.00,0.69)	2.05 (0.03,153.09)	MA							
0.34 (0.08,1.49)	0.89 (0.00,247.42)	0.00 (0.00,0.10)	1.82 (0.27,12.20)	0.89 (0.02,49.29)	MA+CHM						
73.51 (3.05,1769.98)	194.74 (0.36,105142.58)	0.84 (0.23,3.07)	398.42 (13.35,11892.88)	194.75 (1.45,26198.09)	218.68 (10.85,4408.30)	MA+MOX					
21.88 (0.64,752.05)	57.97 (0.09,37743.69)	0.25 (0.06,1.00)	118.60 (2.84,4951.44)	57.97 (0.34,9895.42)	65.09 (2.22,1908.74)	0.30 (0.06,1.40)	MA+UR				
3.11 (0.12,81.39)	8.25 (0.01,4642.01)	0.04 (0.00,2.84)	16.87 (0.52,543.68)	8.25 (0.06,1170.22)	9.26 (0.42,203.49)	0.04 (0.00,2.77)	0.14 (0.00,12.26)	MOX			
34.62 (0.89,1349.05)	91.72 (0.13,64009.12)	0.40 (0.07,2.13)	187.64 (3.99,8826.02)	91.72 (0.49,17082.95)	102.99 (3.08,3443.50)	0.47 (0.08,2.90)	1.58 (0.24,10.40)	11.12 (0.12,1059.07)	MOX+UR		
25.97 (0.92,736.80)	68.80 (0.12,40424.11)	0.30 (0.14,0.64)	140.76 (4.04,4902.31)	68.80 (0.46,10312.05)	77.26 (3.22,1852.20)	0.35 (0.13,1.00)	1.19 (0.38,3.74)	8.34 (0.11,618.11)	0.75 (0.17,3.33)	UR	
0.39 (0.11,1.40)	1.03 (0.00,271.15)	0.00 (0.00,0.11)	2.10 (0.36,12.08)	1.03 (0.02,52.94)	1.15 (0.55,2.42)	0.01 (0.00,0.10)	0.02 (0.00,0.48)	0.12 (0.01,2.49)	0.01 (0.00,0.35)	0.01 (0.00,0.33)	WM

Supplementary Section S9: CINeMA assessment

We used the CINeMA web application to assess the certainty of evidence for each network estimate based on the following criteria:

A:Within-study bias: The overall RoB 2 judgment for each study was mapped to the CINeMA within-study bias categories, with low risk, some concerns, and high risk corresponding to low, moderate, and high risk, respectively. See Supplementary Section S4 (Table S4.1) for the study-level risk-of-bias assessment. The risk of bias for a pairwise comparison of each intervention is shown in Figures S4.1–S4.2.

Figure S4.1. Risk of bias contribution by treatment group in PASI.

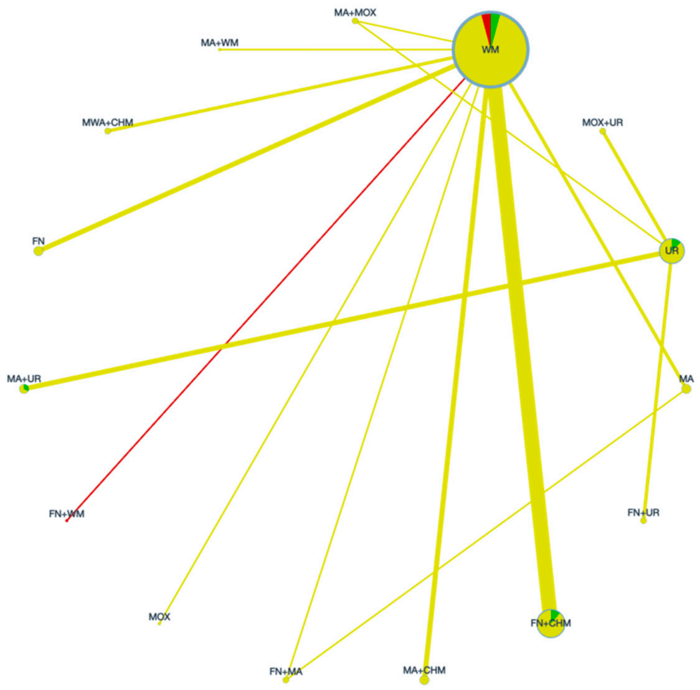

Figure S4.2. Overall risk of bias by treatment comparison in PASI.

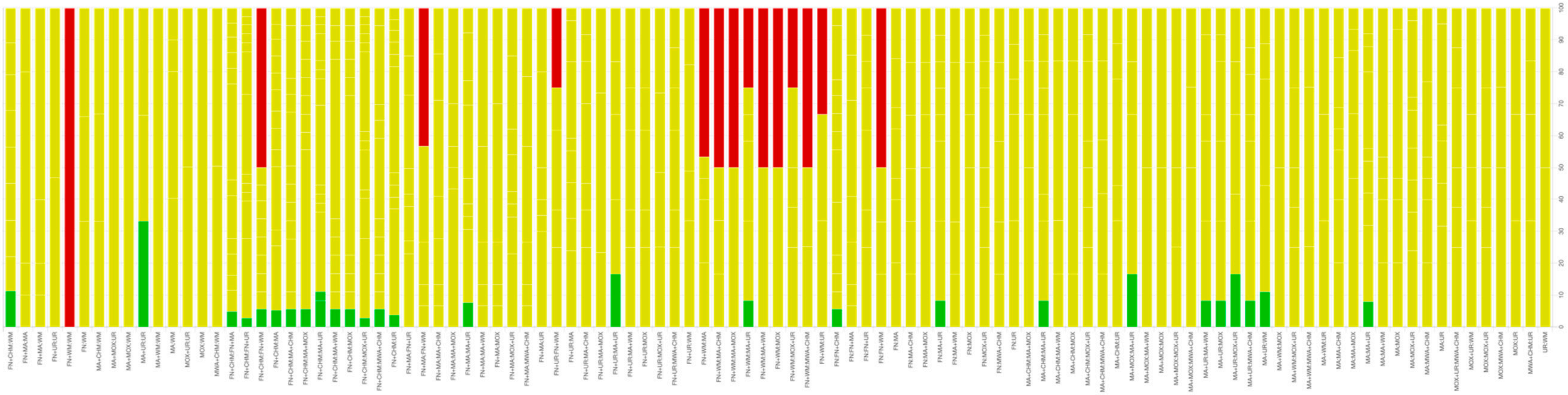

Table S8.1. Transitivity (Indirectness) Assessment.

	Baseline variables (Mean ± SD)		
Intervention	Age (years)	Disease duration (years)	Treatment duration (weeks)
FN	39.00 ± 14.86	2.85 ± 3.82	8.00 ± 0.00
FN+CHM	40.74 ± 9.96	4.23 ± 3.07	5.71 ± 2.91
FN+MA	39.20 ± 7.01	5.89 ± 3.26	8.00 ± 0.00
FN+UR	35.58 ± 8.78	4.27 ± 2.65	6.53 ± 1.51
FN+WM	38.60 ± 11.30	14.20 ± 9.50	4.00 ± 0.00
MA	38.64 ± 8.74	8.56 ± 7.33	8.00 ± 0.00
MA+CHM	37.98 ± 5.75	4.04 ± 2.78	9.52 ± 1.95
MA+MOX	42.84 ± 9.30	2.69 ± 2.16	12.00 ± 0.00
MA+UR	42.67 ± 9.99	10.03 ± 4.21	8.00 ± 0.00
MA+WM	36.90 ± 6.30	4.60 ± 2.50	12.00 ± 0.00
MOX	41.85 ± 9.89	10.11 ± 10.83	6.24 ± 2.00
MOX+UR	43.25 ± 9.46	2.90 ± 1.73	3.00 ± 0.00
MWA+CHM	37.70 ± 8.42	7.60 ± 2.04	12.00 ± 0.00
UR	41.26 ± 10.04	6.16 ± 4.60	7.12 ± 2.69
WM	39.62 ± 9.88	5.70 ± 5.94	7.72 ± 3.23

Abbreviations: FN, fire needle; CHM, Chinese herbal medicine; MA, manual acupuncture; MOX, moxibustion; MWA, microwave acupuncture; UR, ultraviolet radiation/phototherapy; WM, Western medicine.

Imprecision: Imprecision was assessed in CINeMA by comparing the 95% confidence interval of each network estimate with the prespecified clinical equivalence range of SMD −0.10 to 0.10. Judgments were made according to the CINeMA framework.

Heterogeneity: Heterogeneity was assessed by considering the 95% confidence interval and 95% prediction interval in relation to the same prespecified clinical equivalence range (SMD −0.10 to 0.10), following the CINeMA framework.

Incoherence: For comparisons with both direct and indirect evidence, incoherence was assessed using SIDE (node-splitting) analyses. The magnitude and uncertainty of the difference between direct and indirect estimates were considered in relation to the prespecified clinical equivalence range of SMD −0.10 to 0.10. The SIDE P value was considered as supporting statistical information rather than as the sole basis for grading. The global design-by-treatment interaction test was additionally used to assess inconsistency at the network level. For PASI, the global test showed no statistical evidence of inconsistency (P = 0.5381).

Table S8.2. CINeMA Results of PASI.

Comparison	Within-study bias	Reporting bias	Indirectness	Imprecision	Heterogeneity	Incoherence	Confidence rating
FN:WM	Some concerns	Undetected	Some concerns	No concerns	Major concerns	Not estimable	Very low
FN+CHM:WM	Some concerns	Suspected	Some concerns	No concerns	Major concerns	Not estimable	Very low
FN+MA:MA	Some concerns	Suspected	Some concerns	Major concerns	No concerns	No concerns	Very low
FN+MA:WM	Some concerns	Suspected	Some concerns	No concerns	Major concerns	No concerns	Very low
FN+UR:UR	Some concerns	Undetected	Some concerns	No concerns	No concerns	Not estimable	Low
FN+WM:WM	Major concerns	Suspected	Some concerns	Major concerns	No concerns	Not estimable	Very low
MA:WM	Some concerns	Suspected	Some concerns	Major concerns	No concerns	No concerns	Very low
MA+CHM:WM	Some concerns	Undetected	Some concerns	No concerns	Major concerns	Not estimable	Very low
MA+MOX:UR	Some concerns	Suspected	Some concerns	Major concerns	No concerns	Not estimable	Very low
MA+MOX:WM	Some concerns	Suspected	Some concerns	Major concerns	No concerns	Not estimable	Very low
MA+UR:UR	Some concerns	Undetected	Some concerns	No concerns	Major concerns	Not estimable	Very low
MA+WM:WM	Some concerns	Suspected	Some concerns	No concerns	Major concerns	Not estimable	Very low
MOX:WM	Some concerns	Suspected	Some concerns	Major concerns	No concerns	Not estimable	Very low
MOX+UR:UR	Some concerns	Undetected	Some concerns	No concerns	Major concerns	Not estimable	Very low

Comparison	Within-study bias	Reporting bias	Indirectness	Imprecision	Heterogeneity	Incoherence	Confidence rating
MWA+CHM:WM	Some concerns	Undetected	Some concerns	Major concerns	No concerns	Not estimable	Very low
FN:FN+CHM	Some concerns	Suspected	Some concerns	Major concerns	No concerns	Not estimable	Very low
FN:FN+MA	Some concerns	Suspected	Some concerns	Major concerns	No concerns	Not estimable	Very low
FN:FN+UR	Some concerns	Suspected	Some concerns	Major concerns	No concerns	Not estimable	Very low
FN:FN+WM	Some concerns	Suspected	Some concerns	Major concerns	No concerns	Not estimable	Very low
FN:MA	Some concerns	Suspected	Some concerns	Major concerns	No concerns	Not estimable	Very low
FN:MA+CHM	Some concerns	Undetected	Some concerns	Major concerns	No concerns	Not estimable	Very low
FN:MA+MOX	Some concerns	Suspected	Some concerns	Major concerns	No concerns	Not estimable	Very low
FN:MA+UR	Some concerns	Suspected	Some concerns	Major concerns	No concerns	Not estimable	Very low
FN:MA+WM	Some concerns	Suspected	Some concerns	Major concerns	No concerns	Not estimable	Very low
FN:MOX	Some concerns	Suspected	Some concerns	Major concerns	No concerns	Not estimable	Very low
FN:MOX+UR	Some concerns	Suspected	Some concerns	Major concerns	No concerns	Not estimable	Very low
FN:MWA+CHM	Some concerns	Undetected	Some concerns	Major concerns	No concerns	Not estimable	Very low
FN:UR	Some concerns	Suspected	Some concerns	Major concerns	No concerns	Not estimable	Very low
FN+CHM:FN+MA	Some concerns	Suspected	Some concerns	Major concerns	No concerns	Not estimable	Very low
FN+CHM:FN+UR	Some concerns	Suspected	Some concerns	Major concerns	No concerns	Not estimable	Very low
FN+CHM:FN+WM	Major concerns	Suspected	Some concerns	Major concerns	No concerns	Not estimable	Very low
FN+CHM:MA	Some concerns	Suspected	Some concerns	Major concerns	No concerns	Not estimable	Very low
FN+CHM:MA+CHM	Some concerns	Suspected	Some concerns	Major concerns	No concerns	Not estimable	Very low
FN+CHM:MA+MOX	Some concerns	Suspected	Some concerns	Major concerns	No concerns	Not estimable	Very low
FN+CHM:MA+UR	Some concerns	Suspected	Some concerns	Major concerns	No concerns	Not estimable	Very low
FN+CHM:MA+WM	Some concerns	Suspected	Some concerns	Major concerns	No concerns	Not estimable	Very low
FN+CHM:MOX	Some concerns	Suspected	Some concerns	Major concerns	No concerns	Not estimable	Very low
FN+CHM:MOX+UR	Some concerns	Suspected	Some concerns	Major concerns	No concerns	Not estimable	Very low
FN+CHM:MWA+CHM	Some concerns	Suspected	Some concerns	Major concerns	No concerns	Not estimable	Very low
FN+CHM:UR	Some concerns	Suspected	Some concerns	Major concerns	No concerns	Not estimable	Very low
FN+MA:FN+UR	Some concerns	Suspected	Some concerns	Major concerns	No concerns	Not estimable	Very low
FN+MA:FN+WM	Some concerns	Suspected	Some concerns	Major concerns	No concerns	Not estimable	Very low
FN+MA:MA+CHM	Some concerns	Suspected	Some concerns	Major concerns	No concerns	Not estimable	Very low
FN+MA:MA+MOX	Some concerns	Suspected	Some concerns	Major concerns	No concerns	Not estimable	Very low
FN+MA:MA+UR	Some concerns	Suspected	Some concerns	Major concerns	No concerns	Not estimable	Very low
FN+MA:MA+WM	Some concerns	Suspected	Some concerns	Major concerns	No concerns	Not estimable	Very low
FN+MA:MOX	Some concerns	Suspected	Some concerns	Major concerns	No concerns	Not estimable	Very low
FN+MA:MOX+UR	Some concerns	Suspected	Some concerns	Major concerns	No concerns	Not estimable	Very low
FN+MA:MWA+CHM	Some concerns	Suspected	Some concerns	Major concerns	No concerns	Not estimable	Very low

Comparison	Within-study bias	Reporting bias	Indirectness	Imprecision	Heterogeneity	Incoherence	Confidence rating
FN+MA:UR	Some concerns	Suspected	Some concerns	Major concerns	No concerns	Not estimable	Very low
FN+UR:FN+WM	Some concerns	Suspected	Some concerns	Major concerns	No concerns	Not estimable	Very low
FN+UR:MA	Some concerns	Suspected	Some concerns	Major concerns	No concerns	Not estimable	Very low
FN+UR:MA+CHM	Some concerns	Suspected	Some concerns	Major concerns	No concerns	Not estimable	Very low
FN+UR:MA+MOX	Some concerns	Suspected	Some concerns	Major concerns	No concerns	Not estimable	Very low
FN+UR:MA+UR	Some concerns	Undetected	Some concerns	No concerns	Major concerns	Not estimable	Very low
FN+UR:MA+WM	Some concerns	Suspected	Some concerns	Major concerns	No concerns	Not estimable	Very low
FN+UR:MOX	Some concerns	Suspected	Some concerns	Major concerns	No concerns	Not estimable	Very low
FN+UR:MOX+UR	Some concerns	Undetected	Some concerns	Some concerns	Some concerns	Not estimable	Very low
FN+UR:MWA+CHM	Some concerns	Suspected	Some concerns	Major concerns	No concerns	Not estimable	Very low
FN+UR:WM	Some concerns	Suspected	Some concerns	Major concerns	No concerns	Not estimable	Very low
FN+WM:MA	Some concerns	Suspected	Some concerns	Major concerns	No concerns	Not estimable	Very low
FN+WM:MA+CHM	Some concerns	Suspected	Some concerns	Major concerns	No concerns	Not estimable	Very low
FN+WM:MA+MOX	Some concerns	Suspected	Some concerns	Major concerns	No concerns	Not estimable	Very low
FN+WM:MA+UR	Some concerns	Suspected	Some concerns	Major concerns	No concerns	Not estimable	Very low
FN+WM:MA+WM	Some concerns	Suspected	Some concerns	Major concerns	No concerns	Not estimable	Very low
FN+WM:MOX	Some concerns	Suspected	Some concerns	Major concerns	No concerns	Not estimable	Very low
FN+WM:MOX+UR	Some concerns	Suspected	Some concerns	Major concerns	No concerns	Not estimable	Very low
FN+WM:MWA+CHM	Some concerns	Suspected	Some concerns	Major concerns	No concerns	Not estimable	Very low
FN+WM:UR	Some concerns	Suspected	Some concerns	Major concerns	No concerns	Not estimable	Very low
MA:MA+CHM	Some concerns	Suspected	Some concerns	Major concerns	No concerns	Not estimable	Very low
MA:MA+MOX	Some concerns	Suspected	Some concerns	Major concerns	No concerns	Not estimable	Very low
MA:MA+UR	Some concerns	Suspected	Some concerns	Major concerns	No concerns	Not estimable	Very low
MA:MA+WM	Some concerns	Suspected	Some concerns	Major concerns	No concerns	Not estimable	Very low
MA:MOX	Some concerns	Suspected	Some concerns	Major concerns	No concerns	Not estimable	Very low
MA:MOX+UR	Some concerns	Suspected	Some concerns	Major concerns	No concerns	Not estimable	Very low
MA:MWA+CHM	Some concerns	Suspected	Some concerns	Major concerns	No concerns	Not estimable	Very low
MA:UR	Some concerns	Suspected	Some concerns	Major concerns	No concerns	Not estimable	Very low
MA+CHM:MA+MOX	Some concerns	Suspected	Some concerns	Major concerns	No concerns	Not estimable	Very low
MA+CHM:MA+UR	Some concerns	Suspected	Some concerns	Major concerns	No concerns	Not estimable	Very low
MA+CHM:MA+WM	Some concerns	Suspected	Some concerns	Major concerns	No concerns	Not estimable	Very low
MA+CHM:MOX	Some concerns	Suspected	Some concerns	Major concerns	No concerns	Not estimable	Very low
MA+CHM:MOX+UR	Some concerns	Suspected	Some concerns	Major concerns	No concerns	Not estimable	Very low
MA+CHM:MWA+CHM	Some concerns	Undetected	Some concerns	Major concerns	No concerns	Not estimable	Very low
MA+CHM:UR	Some concerns	Suspected	Some concerns	Major concerns	No concerns	Not estimable	Very low

Comparison	Within-study bias	Reporting bias	Indirectness	Imprecision	Heterogeneity	Incoherence	Confidence rating
MA+MOX:MA+UR	Some concerns	Suspected	Some concerns	Major concerns	No concerns	Not estimable	Very low
MA+MOX:MA+WM	Some concerns	Suspected	Some concerns	Major concerns	No concerns	Not estimable	Very low
MA+MOX:MOX	Some concerns	Suspected	Some concerns	Major concerns	No concerns	Not estimable	Very low
MA+MOX:MOX+UR	Some concerns	Suspected	Some concerns	Major concerns	No concerns	Not estimable	Very low
MA+MOX:MWA+CHM	Some concerns	Suspected	Some concerns	Major concerns	No concerns	Not estimable	Very low
MA+UR:MA+WM	Some concerns	Suspected	Some concerns	Major concerns	No concerns	Not estimable	Very low
MA+UR:MOX	Some concerns	Suspected	Some concerns	Major concerns	No concerns	Not estimable	Very low
MA+UR:MOX+UR	Some concerns	Undetected	Some concerns	Major concerns	No concerns	Not estimable	Very low
MA+UR:MWA+CHM	Some concerns	Suspected	Some concerns	Major concerns	No concerns	Not estimable	Very low
MA+UR:WM	Some concerns	Suspected	Some concerns	Major concerns	No concerns	Not estimable	Very low
MA+WM:MOX	Some concerns	Suspected	Some concerns	Major concerns	No concerns	Not estimable	Very low
MA+WM:MOX+UR	Some concerns	Suspected	Some concerns	Major concerns	No concerns	Not estimable	Very low
MA+WM:MWA+CHM	Some concerns	Suspected	Some concerns	Major concerns	No concerns	Not estimable	Very low
MA+WM:UR	Some concerns	Suspected	Some concerns	Major concerns	No concerns	Not estimable	Very low
MOX:MOX+UR	Some concerns	Suspected	Some concerns	Major concerns	No concerns	Not estimable	Very low
MOX:MWA+CHM	Some concerns	Suspected	Some concerns	Major concerns	No concerns	Not estimable	Very low
MOX:UR	Some concerns	Suspected	Some concerns	Major concerns	No concerns	Not estimable	Very low
MOX+UR:MWA+CHM	Some concerns	Suspected	Some concerns	Major concerns	No concerns	Not estimable	Very low
MOX+UR:WM	Some concerns	Suspected	Some concerns	Major concerns	No concerns	Not estimable	Very low
MWA+CHM:UR	Some concerns	Suspected	Some concerns	Major concerns	No concerns	Not estimable	Very low
UR:WM	Some concerns	Suspected	Some concerns	Major concerns	No concerns	Not estimable	Very low

Supplementary Section S10: Funnel plots

The figures show the assessment of small-study effects in studies evaluating acupuncture-related treatments for psoriasis. The funnel plots include trials comparing at least one acupuncture-related treatment with another acupuncture-related therapy, Western medicine, phototherapy, or a combination treatment.

Figure S5.1. Funnel plot for PASI.

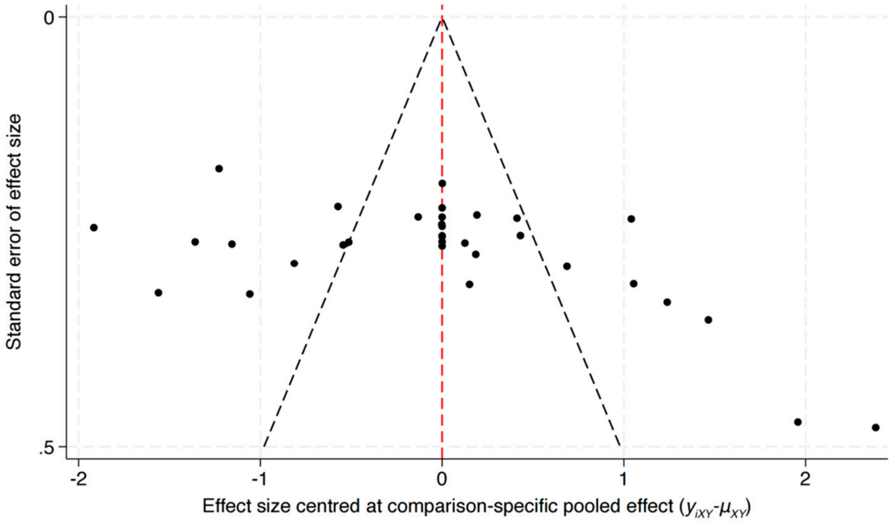

Figure S5.2. Funnel plot for total response rate.

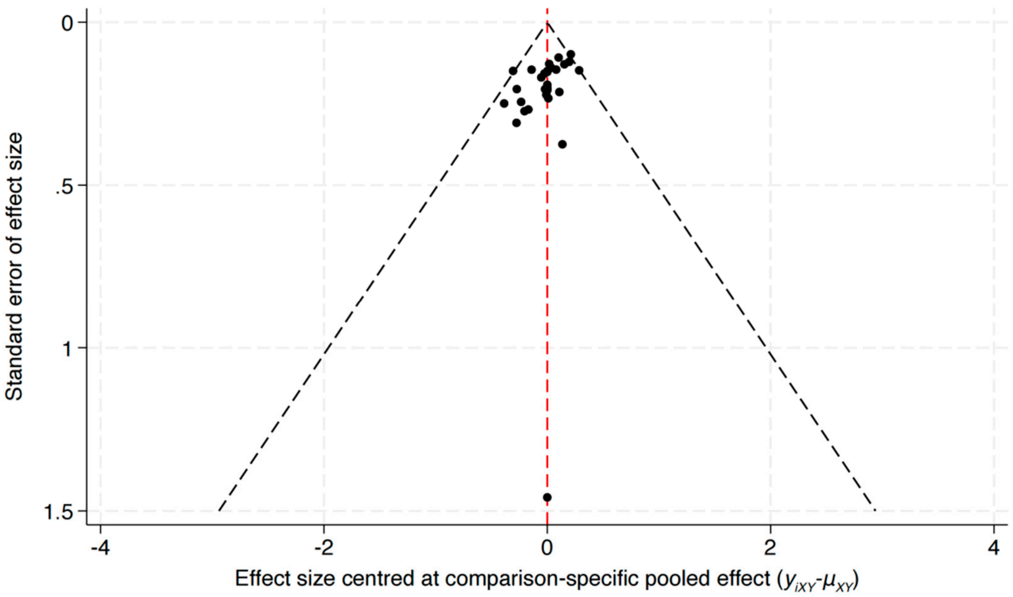

Figure S5.3. Funnel plot for DLQI.

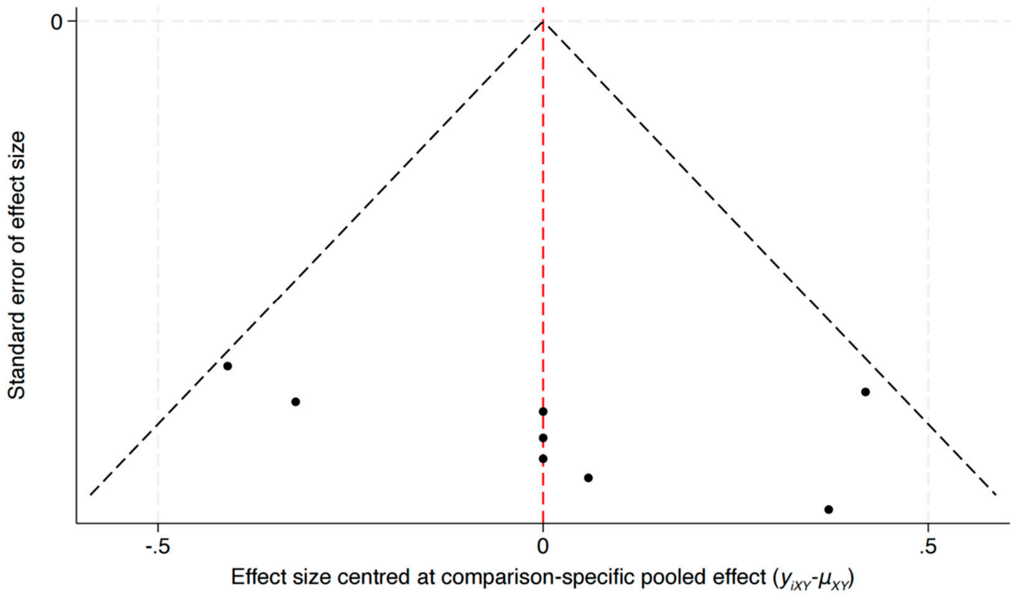

Figure S5.4. Funnel plot for pruritus score.

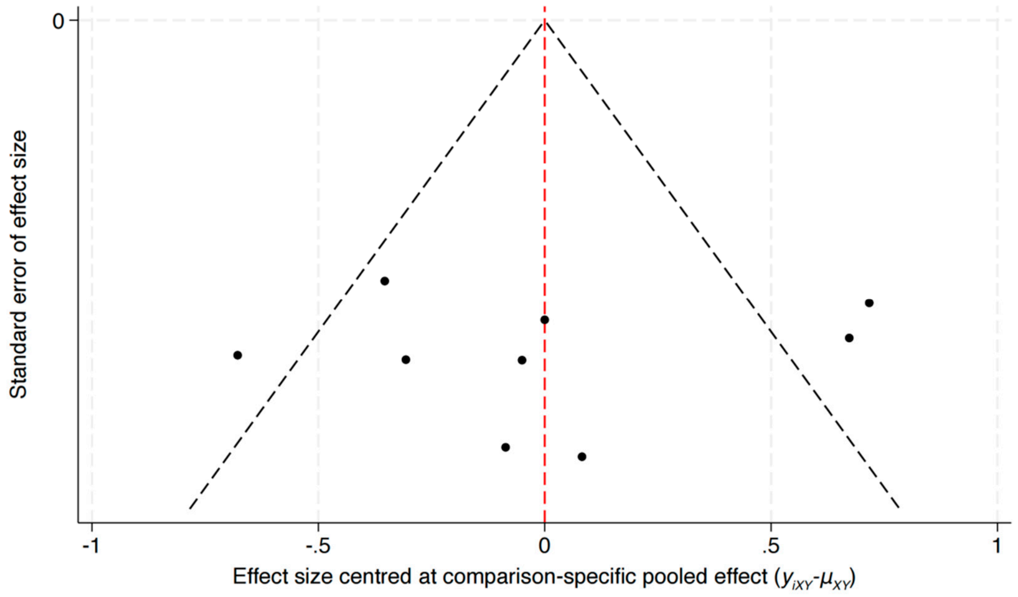

Figure S5.5. Funnel plot for TNF-α.

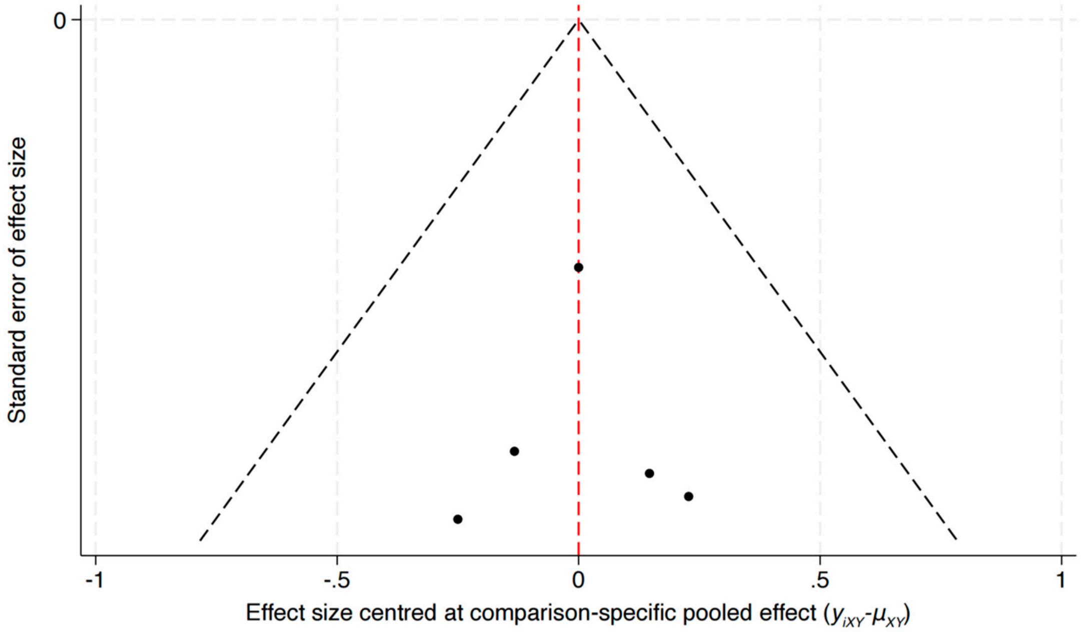

Figure S5.6. Funnel plot for VEGF.

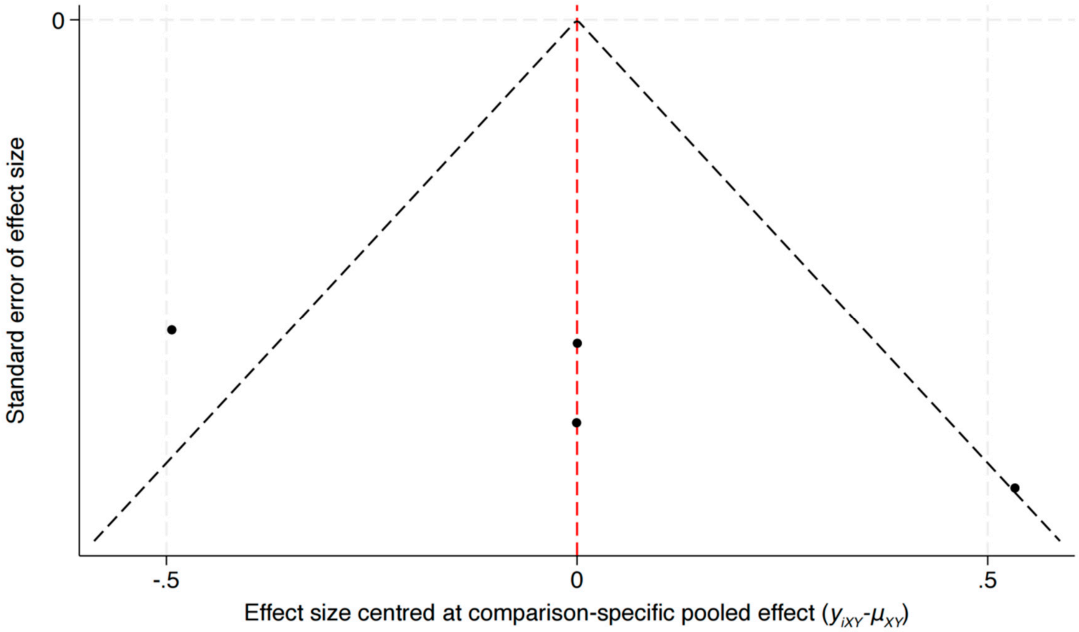

Figure S5.7. Funnel plot for adverse events.

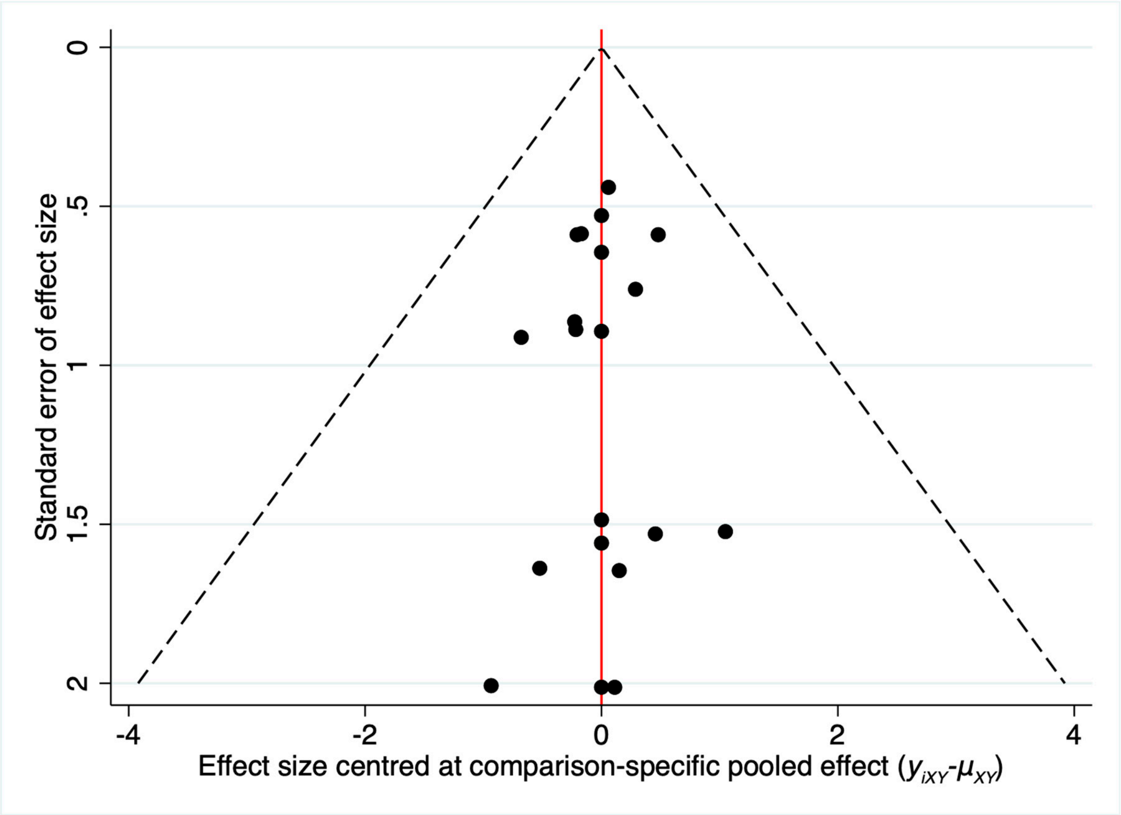

Supplementary Section S11: Sensitivity analyses

Two sensitivity analyses were performed for the PASI outcome to assess the robustness of the primary network meta-analysis. The first sensitivity analysis examined the influence of comparator-node definitions by separating Western medicine (WM) and ultraviolet radiation (UR) into more specific treatment categories. The second sensitivity analysis restricted the network to acupuncture monotherapy or acupuncture add-on therapy to evaluate whether the inclusion of complex combination interventions influenced the main findings.

S11.1. Sensitivity analysis based on comparator-node definitions

In the primary PASI network, different forms of Western medicine and ultraviolet radiation were grouped into the broad WM and UR nodes, respectively. To evaluate the influence of comparator-node definitions, WM was further classified as topical Western medicine (WM-T), systemic Western medicine (WM-S), or combined topical and systemic Western medicine (WM-TS), while UR was separated into NB-UVB and excimer therapy. After disaggregation, the two excimer trials (Zhang Cuixia 2016 and Tong Li 2020) formed a disconnected component and were therefore excluded from this sensitivity network. The analysis was repeated using the same random-effects network meta-analysis model and standardized mean difference (SMD) as in the primary analysis.

Figure S6.1. Flow diagram of the comparator-node sensitivity analysis.

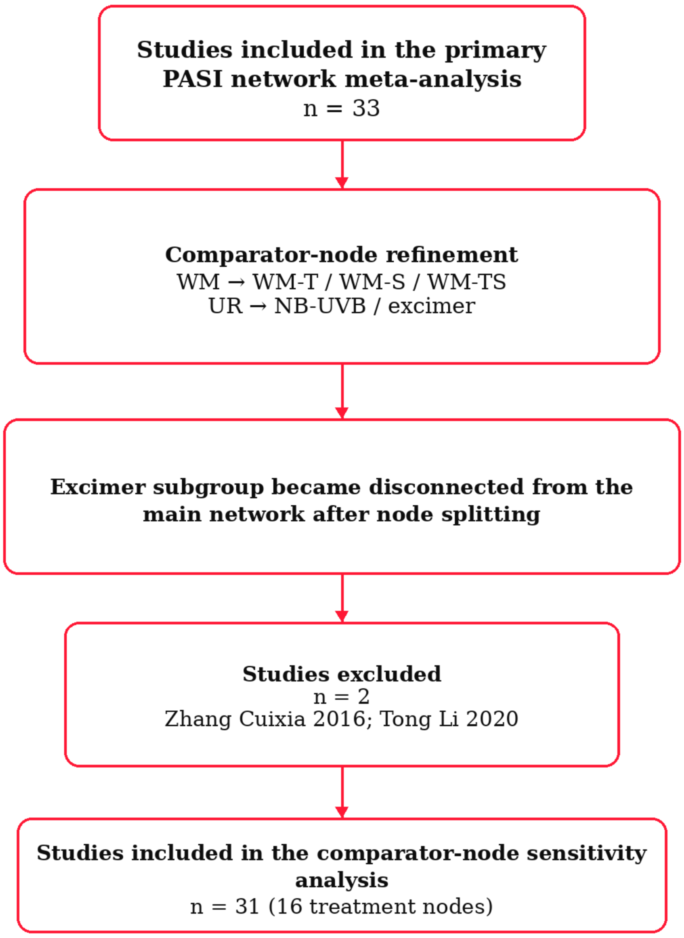

Table S9.1. Baseline characteristics of studies included in the comparator-node sensitivity analysis.

Study	Design	Treatment duration	Number of participants	Randomised treatments	Age (mean ± SD), years	Disease duration (mean ± SD), years	Clinical stage/type
Wu 2011	RCT	12 weeks	60	MA+MOX: 30<br>WM-S: 30	37.38±11.36<br>39.25±10.21	0.76±0.61<br>0.72±0.54	Psoriasis vulgaris, stationary stage
Han 2014	RCT	3 weeks	80	MOX+NB-UVB: 40<br>NB-UVB: 40	46.2±6.8<br>45.6±7.3	3.98±0.90<br>3.62±0.87	Psoriasis vulgaris, stationary stage
Jie 2015	RCT	8 weeks	80	MA: 40<br>WM-T: 40	38.28±10.89<br>41.37±11.04	12.78±8.26<br>13.56±8.71	Psoriasis vulgaris, stationary stage
Zhang Chen 2016	RCT	8 weeks	76	FN+CHM: 38<br>WM-S: 38	51.12±3.87<br>53.02±3.31	7.12±1.02<br>6.54±1.42	Psoriasis vulgaris, stationary stage
Deng 2017	RCT	4 weeks	60	MOX: 30<br>WM-S: 30	40.4±10.9<br>41.7±11.5	5.7±10.6<br>5.4±11.1	Psoriasis vulgaris, stationary stage
Luo 2017	RCT	3 weeks	40	MOX+NB-UVB: 20<br>NB-UVB: 20	37.36±11.32<br>36.96±12.02	0.75±0.60<br>0.74±0.64	Psoriasis vulgaris, stationary stage
Wei 2017	RCT	8 weeks	55	MA+CHM: 27<br>WM-T: 28	32.73±5.36<br>33.24±5.19	4.45±2.38<br>4.39±2.31	Psoriasis vulgaris, stationary stage
Hu 2018	RCT	30 days	120	FN+CHM: 60<br>WM-T: 60	32.2±11.6<br>32.7±9.9	5.2±3.4<br>5.4±2.3	Psoriasis vulgaris, stationary stage
Lv 2019	RCT	2 months	80	FN+MA: 40<br>MA: 40	38±6<br>39±6	4.28±2.72<br>4.33±1.98	Psoriasis vulgaris, stationary stage
Wang 2019	RCT	4 weeks	133	FN+CHM: 67<br>WM-TS: 66	42.6±12.5<br>41.9±11.1	3.3±1.4<br>3.4±1.6	Psoriasis vulgaris, stationary stage
Huang 2020	RCT	12 weeks	80	MA+MOX: 40<br>NB-UVB: 40	46.93±4.11<br>45.62±3.57	4.14±1.72<br>4.52±2.98	Psoriasis vulgaris, stationary stage
Chen 2021	RCT	8 weeks	74	MOX: 38<br>WM-T: 36	43±9<br>41±12	13.6±9.8<br>15.8±12.0	Psoriasis vulgaris, stationary stage
Meng 2021	RCT	30 days	90	FN+WM-T: 45<br>WM-T: 45	38.6±11.3<br>38.6±11.3	14.2±9.5<br>14.2±9.5	Psoriasis vulgaris, stationary stage
Jiang 2022	RCT	8 weeks	63	MA+NB-UVB: 31<br>NB-UVB: 32	38±9<br>37±8	10.93±2.89<br>10.03±3.92	Psoriasis vulgaris, stationary stage
Xiao 2022a	RCT	8 weeks	62	FN: 31<br>WM-T: 31	42.13±15.20<br>45.24±14.02	0.51±0.32<br>0.48±0.25	Psoriasis vulgaris, stationary stage
Yin 2022	RCT	8 weeks	70	FN+MA: 35<br>WM-T: 35	40.57±7.88<br>43.80±6.56	7.74±2.85<br>8.43±3.69	Psoriasis vulgaris, stationary stage
Zhou 2022	RCT	8 weeks	65	MA+NB-UVB: 32<br>NB-UVB: 33	40.09±6.34<br>40.01±6.23	9.96±3.81<br>9.88±3.75	Psoriasis vulgaris, stationary stage
Xiao 2022b	RCT	8 weeks	80	FN: 40<br>WM-T: 40	42±16<br>45±14	0.52±0.39<br>0.47±0.27	Psoriasis vulgaris, stationary stage
Tian 2023	RCT	3 months	112	FN+CHM: 56<br>WM-S: 56	40.47±6.85<br>38.74±6.63	3.45±0.61<br>3.31±0.57	Psoriasis vulgaris, stationary stage
Tong 2023	RCT	4 weeks	97	FN+CHM: 48<br>WM-T: 49	39.6±6.89<br>37.8±6.53	2.03±1.15<br>2.17±1.30	Psoriasis vulgaris, stationary stage
Wang 2023	RCT	12 weeks	124	MWA+CHM: 62<br>WM-S: 62	32.31±4.46<br>32.45±4.11	8.30±2.18<br>8.32±2.12	Psoriasis vulgaris, stationary stage
Yuan 2023	RCT	3 months	86	MA+CHM: 43<br>WM-TS: 43	39.05±4.16<br>38.96±4.12	6.62±1.37<br>6.58±1.34	Psoriasis vulgaris, stationary stage
Cui 2024	RCT	12 weeks	120	MA+WM-S: 60<br>WM-S: 60	36.9±6.3<br>37.8±6.5	4.6±2.5<br>4.7±2.6	Psoriasis vulgaris, stationary stage
Qi 2024	RCT	8 weeks	130	MA+NB-UVB: 65<br>NB-UVB: 65	46.17±10.69<br>48.24±11.23	9.63±4.87<br>9.19±5.61	Psoriasis vulgaris, stationary stage
Wang 2024	RCT	1 month	60	FN+CHM: 30<br>WM-T: 30	45.39±9.47<br>43.76±8.83	2.96±0.80<br>2.68±0.70	Psoriasis vulgaris, stationary stage
Yang 2024	RCT	4 weeks	58	FN+CHM: 28<br>WM-T: 30	40.1±4.79<br>35.2±3.91	2.11±1.05<br>2.02±1.20	Psoriasis vulgaris, stationary stage

Study	Design	Treatment duration	Number of participants	Randomised treatments	Age (mean ± SD), years	Disease duration (mean ± SD), years	Clinical stage/type
Zuo 2024	RCT	12 weeks	80	MWA+CHM: 40<br>WM-S: 40	46.05±5.91<br>46.24±6.11	6.51±1.17<br>6.63±1.21	Psoriasis vulgaris, stationary stage
Gao 2025	RCT	8 weeks	86	MA+CHM: 43<br>WM-T: 43	40.20±5.40<br>39.85±5.59	1.21±0.24<br>1.28±0.26	Psoriasis vulgaris, stationary stage
Huang 2025	RCT	4 weeks	100	FN+CHM: 50<br>WM-T: 50	41.23±3.19<br>41.52±3.69	3.96±0.77<br>3.87±0.62	Psoriasis vulgaris, stationary stage
Li 2025	RCT	8 weeks	60	FN: 30<br>WM-T: 30	31.77±10.06<br>27.84±8.45	8.39±2.19<br>8.22±2.55	Psoriasis vulgaris, stationary stage
Wang 2025	RCT	8 weeks	39	FN+CHM: 20<br>WM-S: 19	36.60±10.58<br>38.74±11.07	11.87±5.97<br>10.21±5.15	Psoriasis vulgaris, stationary stage

Abbreviations: CHM, Chinese herbal medicine; FN, fire needle; MA, manual acupuncture; MOX, moxibustion; MWA, microwave acupuncture; NB-UVB, narrowband ultraviolet B; WM-S, systemic Western medicine; WM-T, topical Western medicine; WM-TS, combined topical and systemic Western medicine; RCT, randomized controlled trial; SD, standard deviation; NR, not reported in the provided source.

Table S9.2. Sensitivity analysis based on comparator-node definitions.

	PASI												
	**FN**	**FN+CHM**	**FN+MA**	**FN+WM-T**	**MA**	**MA+CHM**	**MA+MOX**	**MA+NB-UVB**	**MA+WM-S**	**MOX**	**MOX+NB-UVB**	**MWA+CHM**	**NB-UVB**
**Main estimate**	-1.77 (-3.17, -0.37)	-1.74 (-2.55, -0.93)	-1.47 (-3.44, 0.51)	-0.90 (-3.31, 1.50)	-0.26 (-2.23, 1.71)	-1.79 (-3.19, -0.39)	-0.70 (-3.11, 1.71)	-0.66 (-4.32, 3.00)	-2.44 (-4.85, -0.03)	-0.51 (-2.22, 1.20)	-0.82 (-4.62, 2.98)	-1.11 (-2.81, 0.59)	0.77 (-2.62, 4.15)
**Sensitivity analysis**	-1.77 (-3.07, -0.47)	-1.78 (-2.72, -0.84)	-1.47 (-3.30, 0.36)	-0.90 (-3.13, 1.33)	-0.26 (-2.09, 1.56)	-2.02 (-3.46, -0.59)	-0.57 (-3.23, 2.09)	-0.53 (-4.22, 3.17)	-2.32 (-4.98, 0.35)	-0.45 (-2.19, 1.29)	-0.69 (-4.51, 3.12)	-0.98 (-3.12, 1.16)	0.90 (-2.56, 4.36)

Main-analysis reference: WM. Comparator-node sensitivity-analysis reference: WM-T. Effect estimates are standardized mean differences (SMDs) with 95% confidence intervals (CIs). In the sensitivity analysis, WM was disaggregated into WM-T, WM-S, and WM-TS, and UR-containing nodes were refined to NB-UVB where applicable. The two excimer-laser studies were excluded because the excimer subgroup became disconnected from the main network.

Abbreviations: CHM, Chinese herbal medicine; FN, fire-needle acupuncture; MA, manual acupuncture; MOX, moxibustion; MWA, microwave acupuncture; NB-UVB, narrowband ultraviolet B; UR, ultraviolet radiation; WM, Western medicine; WM-S, systemic Western medicine; WM-T, topical Western medicine.

Table S9.3. SUCRA rankings of treatments in the comparator-node sensitivity analysis.

Treatment	SUCRA	PrBest	MeanRank
**MA+WM-S**	81.1	37.1	3.8
**MA+CHM**	79.9	17.3	4
**FN+CHM**	75.3	2.7	4.7
**FN**	73.5	8.7	5
**FN+MA**	65.5	8.7	6.2
**MWA+CHM**	52.8	2.7	8.1
**FN+WM-T**	51	5.8	8.3
**MOX+NB-UVB**	49.7	9.1	8.5
**MA+NB-UVB**	45.8	4.9	9.1
**WM-TS**	45.3	0.9	9.2
**MA+MOX**	43.9	1.3	9.4
**MOX**	39.6	0.2	10.1
**MA**	34.6	0.5	10.8
**WM-T**	25.1	0	12.2
**WM-S**	22.2	0	12.7
**NB-UVB**	14.6	0	13.8

Abbreviation: SUCRA, surface under the cumulative ranking curve.

S11.2. Sensitivity analysis restricted to acupuncture-focused interventions with non-acupuncture comparators

A second sensitivity analysis was conducted to evaluate the findings in a more acupuncture-focused network. Eligible studies evaluated acupuncture monotherapy, combinations of acupuncture techniques, or acupuncture add-on regimens, with a non-acupuncture comparator (WM or UR). Fourteen studies involving CHM-containing interventions were excluded. In addition, Lv Shiqi 2019 (FN+MA vs MA) was excluded because the comparator itself was an active acupuncture intervention, whereas this sensitivity analysis was restricted to non-acupuncture comparators. The analysis was repeated using the same random-effects model and SMD as in the primary analysis.

Figure S6.2. Flow diagram of the acupuncture-focused sensitivity analysis.

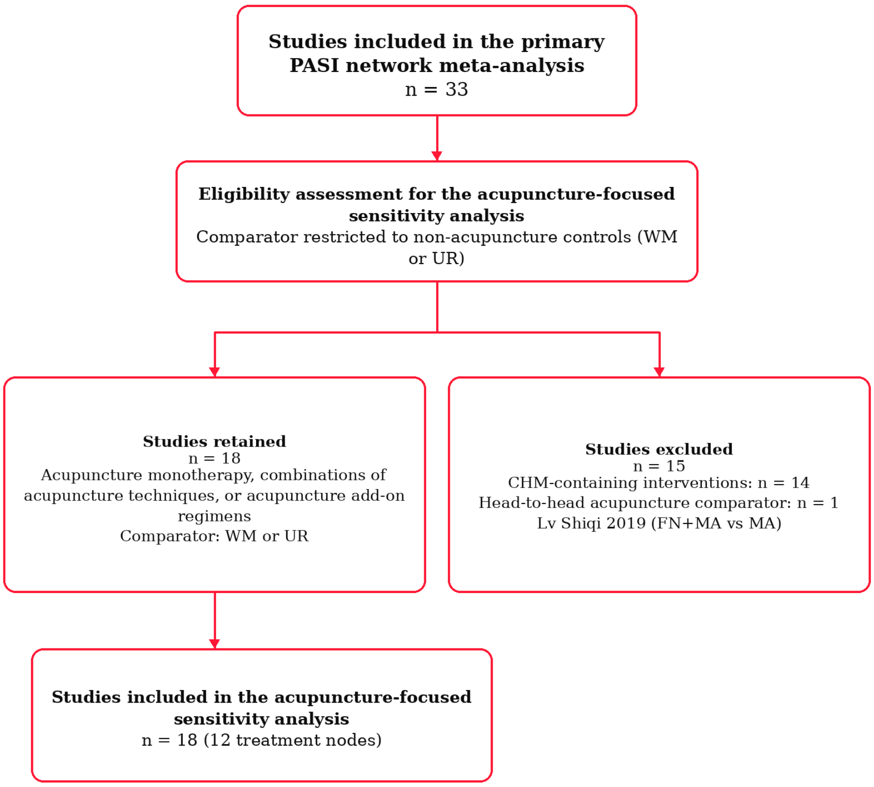

Table S9.4 Baseline characteristics of studies included in the acupuncture-focused sensitivity analysis.

Study	Design	Treatment duration	Number of participants	Randomised treatments	Age (mean ± SD), years	Disease duration (mean ± SD), years	Clinical stage/type
Wu 2011	RCT	12 weeks	60	MA+MOX: 30<br>WM: 30	37.38±11.36<br>39.25±10.21	0.76±0.61<br>0.72±0.54	Psoriasis vulgaris, stationary stage
Han 2014	RCT	3 weeks	80	MOX+UR: 40<br>UR: 40	46.2±6.8<br>45.6±7.3	3.98±0.90<br>3.62±0.87	Psoriasis vulgaris, stationary stage
Jie 2015	RCT	8 weeks	80	MA: 40<br>WM: 40	38.28±10.89<br>41.37±11.04	12.78±8.26<br>13.56±8.71	Psoriasis vulgaris, stationary stage
Zhang Cuixia 2016	RCT	8 weeks	95	FN+UR: 50<br>UR: 45	33.2±10.6<br>31.2±9.7	6.1±2.4<br>6.5±2.1	Psoriasis vulgaris, stationary stage
Deng 2017	RCT	4 weeks	60	MOX: 30<br>WM: 30	40.4±10.9<br>41.7±11.5	5.7±10.6<br>5.4±11.1	Psoriasis vulgaris, stationary stage
Luo 2017	RCT	3 weeks	40	MOX+UR: 20<br>UR: 20	37.36±11.32<br>36.96±12.02	0.75±0.60<br>0.74±0.64	Psoriasis vulgaris, stationary stage
Huang 2020	RCT	12 weeks	80	MA+MOX: 40<br>UR: 40	46.93±4.11<br>45.62±3.57	4.14±1.72<br>4.52±2.98	Psoriasis vulgaris, stationary stage
Tong 2020	RCT	5 weeks	96	FN+UR: 48<br>UR: 48	38.05±5.44<br>39.48±5.28	2.37±1.12<br>2.32±1.34	Psoriasis vulgaris, stationary stage
Chen 2021	RCT	8 weeks	74	MOX: 38<br>WM: 36	43±9<br>41±12	13.6±9.8<br>15.8±12.0	Psoriasis vulgaris, stationary stage
Meng 2021	RCT	30 days	90	FN+WM: 45<br>WM: 45	38.6±11.3<br>38.6±11.3	14.2±9.5<br>14.2±9.5	Psoriasis vulgaris, stationary stage
Jiang 2022	RCT	8 weeks	63	MA+UR: 31<br>UR: 32	38±9<br>37±8	10.93±2.89<br>10.03±3.92	Psoriasis vulgaris, stationary stage
Xiao 2022a	RCT	8 weeks	62	FN: 31<br>WM: 31	42.13±15.20<br>45.24±14.02	0.51±0.32<br>0.48±0.25	Psoriasis vulgaris, stationary stage
Yin 2022	RCT	8 weeks	70	FN+MA: 35<br>WM: 35	40.57±7.88<br>43.80±6.56	7.74±2.85<br>8.43±3.69	Psoriasis vulgaris, stationary stage
Zhou 2022	RCT	8 weeks	65	MA+UR: 32<br>UR: 33	40.09±6.34<br>40.01±6.23	9.96±3.81<br>9.88±3.75	Psoriasis vulgaris, stationary stage
Xiao 2022b	RCT	8 weeks	80	FN: 40<br>WM: 40	42±16<br>45±14	0.52±0.39<br>0.47±0.27	Psoriasis vulgaris, stationary stage
Cui 2024	RCT	12 weeks	120	MA+WM: 60<br>WM: 60	36.9±6.3<br>37.8±6.5	4.6±2.5<br>4.7±2.6	Psoriasis vulgaris, stationary stage
Qi 2024	RCT	8 weeks	130	MA+UR: 65<br>UR: 65	46.17±10.69<br>48.24±11.23	9.63±4.87<br>9.19±5.61	Psoriasis vulgaris, stationary stage
Li 2025	RCT	8 weeks	60	FN: 30<br>WM: 30	31.77±10.06<br>27.84±8.45	8.39±2.19<br>8.22±2.55	Psoriasis vulgaris, stationary stage

Abbreviations: FN, fire needle; MA, manual acupuncture; MOX, moxibustion; UR, ultraviolet radiation; WM, Western medicine; RCT, randomized controlled trial; SD, standard deviation; NR, not reported in the provided source.

Table S9.5. Acupuncture-focused sensitivity analysis of PASI.

	PASI										
	**FN**	**FN+MA**	**FN+UR**	**FN+WM**	**MA**	**MA+MOX**	**MA+UR**	**MA+WM**	**MOX**	**MOX+UR**	**UR**
**Main estimate**	-1.77 (-3.17, -0.37)	-1.47 (-3.44, 0.51)	-2.90 (-6.70, 0.91)	-0.90 (-3.31, 1.50)	-0.26 (-2.23, 1.71)	-0.70 (-3.11, 1.71)	-0.66 (-4.32, 3.00)	-2.44 (-4.85, -0.03)	-0.51 (-2.22, 1.20)	-0.82 (-4.62, 2.98)	0.77 (-2.62, 4.15)
**Sensitivity analysis**	-1.78 (-3.42, -0.14)	-1.02 (-3.86, 1.82)	-2.93 (-7.38, 1.51)	-0.90 (-3.73, 1.92)	-0.71 (-3.53, 2.12)	-0.71 (-3.52, 2.11)	-0.67 (-4.94, 3.60)	-2.44 (-5.27, 0.39)	-0.51 (-2.52, 1.49)	-0.83 (-5.27, 3.60)	0.76 (-3.20, 4.71)

Reference: WM. Effect estimates are standardized mean differences (SMDs) with 95% confidence intervals (CIs). The acupuncture-focused sensitivity analysis excluded 14 trials involving CHM-containing interventions and one head-to-head acupuncture comparison (Lv 2019, FN+MA vs MA).

Abbreviations: FN, fire-needle acupuncture; MA, manual acupuncture; MOX, moxibustion; UR, ultraviolet radiation; WM, Western medicin

Table S9.6. SUCRA rankings of treatments in the acupuncture-focused sensitivity analysis.

Treatment	SUCRA	PrBest	MeanRank
**FN+UR**	82.3	41.8	2.9
**MA+WM**	77.1	30.7	3.5
**FN**	68	7.2	4.5
**FN+MA**	51.8	6.3	6.3
**FN+WM**	49.3	5.4	6.6
**MOX+UR**	49	1.7	6.6
**MA+MOX**	46	0.8	6.9
**MA**	45.3	4.4	7
**MA+UR**	44.8	0.9	7.1
**MOX**	41.8	0.7	7.4
**WM**	27.7	0	9
**UR**	16.9	0	10.1

Abbreviation: SUCRA, surface under the cumulative ranking curve.

Supplementary Section S12: PRISMA-NMA checklist

Section/Topic	Item #	Checklist Item	Reported on Page #
**TITLE**			
Title	1	Identify the report as a systematic review *incorporating a network meta-analysis (or related form of meta-analysis).*	1
**ABSTRACT**			
Structured summary	2	Provide a structured summary including, as applicable:<br>**Background:** main objectives<br>**Methods:** data sources; study eligibility criteria, participants, and interventions; study appraisal; and *synthesis methods, such as network meta-analysis.*<br>**Results:** number of studies and participants identified; summary estimates with corresponding confidence/credible intervals; *treatment rankings may also be discussed. Authors may choose to summarize pairwise comparisons against a chosen treatment included in their analyses for brevity.*<br>**Discussion/Conclusions:** limitations; conclusions and implications of findings.<br>**Other:** primary source of funding; systematic review registration number with registry name.	1-2
**INTRODUCTION**			
Rationale	3	Describe the rationale for the review in the context of what is already known*, including mention of why a network meta-analysis has been conducted.*_	3
Objectives	4	Provide an explicit statement of questions being addressed, with reference to participants, interventions, comparisons, outcomes, and study design (PICOS).	4
**METHODS**			
Protocol and registration	5	Indicate whether a review protocol exists and if and where it can be accessed (e.g., Web address); and, if available, provide registration information, including registration number.	5
Eligibility criteria	6	Specify study characteristics (e.g., PICOS,	5

		length of follow-up) and report characteristics (e.g., years considered, language, publication status) used as criteria for eligibility, giving rationale. *Clearly describe eligible treatments included in the treatment network, and note whether any have been clustered or merged into the same node (with justification).*	
Information sources	7	Describe all information sources (e.g., databases with dates of coverage, contact with study authors to identify additional studies) in the search and date last searched.	6
Search	8	Present full electronic search strategy for at least one database, including any limits used, such that it could be repeated.	6&Supplementary Section S1
Study selection	9	State the process for selecting studies (i.e., screening, eligibility, included in systematic review, and, if applicable, included in the meta-analysis).	7
Data collection process	10	Describe method of data extraction from reports (e.g., piloted forms, independently, in duplicate) and any processes for obtaining and confirming data from investigators.	7
Data items	11	List and define all variables for which data were sought (e.g., PICOS, funding sources) and any assumptions and simplifications made.	8&Supplementary Section S3
**Geometry of the network**	**S1**	Describe methods used to explore the geometry of the treatment network under study and potential biases related to it. This should include how the evidence base has been graphically summarized for presentation, and what characteristics were compiled and used to describe the evidence base to readers.	8
Risk of bias within individual studies	12	Describe methods used for assessing risk of bias of individual studies (including specification of whether this was done at the study or outcome level), and how this information is to be used in any data synthesis.	7&Supplementary Section S4
Summary measures	13	State the principal summary measures (e.g., risk ratio, difference in means). *Also describe the use of additional summary measures assessed, such as treatment rankings and surface under the cumulative ranking curve (SUCRA) values, as well as modified approaches used to present summary findings from meta-analyses.*	8-9
Planned methods of analysis	14	Describe the methods of handling data and combining results of studies for each network meta-analysis. This should include, but not be limited to:<br>• *Handling of multi-arm trials;*<br>• *Selection of variance structure;*	8-9

		• *Selection of prior distributions in Bayesian analyses; and*<br>• *Assessment of model fit.*	
**Assessment of Inconsistency**	**S2**	Describe the statistical methods used to evaluate the agreement of direct and indirect evidence in the treatment network(s) studied. Describe efforts taken to address its presence when found.	8&Supplementary Section S5
Risk of bias across studies	15	Specify any assessment of risk of bias that may affect the cumulative evidence (e.g., publication bias, selective reporting within studies).	8
Additional analyses	16	Describe methods of additional analyses if done, indicating which were pre-specified. This may include, but not be limited to, the following:<br>• Sensitivity or subgroup analyses;<br>• Meta-regression analyses;<br>• *Alternative formulations of the treatment network; and*<br>• *Use of alternative prior distributions for Bayesian analyses (if applicable).*_	20&Supplementary Section S11
**RESULTS†**			
Study selection	17	Give numbers of studies screened, assessed for eligibility, and included in the review, with reasons for exclusions at each stage, ideally with a flow diagram.	9
**Presentation of network structure**	**S3**	Provide a network graph of the included studies to enable visualization of the geometry of the treatment network.	16&Supplementary Section S6
**Summary of network geometry**	**S4**	Provide a brief overview of characteristics of the treatment network. This may include commentary on the abundance of trials and randomized patients for the different interventions and pairwise comparisons in the network, gaps of evidence in the treatment network, and potential biases reflected by the network structure.	10,&16-20
Study characteristics	18	For each study, present characteristics for which data were extracted (e.g., study size, PICOS, follow-up period) and provide the citations.	10-13&Supplementary Section S2

Risk of bias within studies	19	Present data on risk of bias of each study and, if available, any outcome level assessment.	14&Supplementary Section S4
Results of individual studies	20	For all outcomes considered (benefits or harms), present, for each study: 1) simple summary data for each intervention group, and 2) effect estimates and confidence intervals. *Modified approaches may be needed to deal with information from larger networks.*	Supplementary Section S2
Synthesis of results	21	Present results of each meta-analysis done, including confidence/credible intervals. *In larger networks, authors may focus on comparisons versus a particular comparator (e.g. placebo or standard care), with full findings presented in an appendix. League tables and forest plots may be considered to summarize pairwise comparisons.* If additional summary measures were explored (such as treatment rankings), these should also be presented.	15-19&Supplementary Sections S6–S8
**Exploration for inconsistency**	**S5**	Describe results from investigations of inconsistency. This may include such information as measures of model fit to compare consistency and inconsistency models, *P* values from statistical tests, or	15
Risk of bias across studies	22	Present results of any assessment of risk of bias across studies for the evidence base being studied.	Supplementary Section S10
Results of additional analyses	23	Give results of additional analyses, if done (e.g., sensitivity or subgroup analyses, meta-regression analyses*, alternative network geometries studied, alternative choice of prior distributions for Bayesian analyses,* and so forth).	20&Supplementary Section S11
**DISCUSSION**			
Summary of evidence	24	Summarize the main findings, including the strength of evidence for each main outcome; consider their relevance to key groups (e.g., healthcare providers, users, and policy-makers).	20-23
Limitations	25	Discuss limitations at study and outcome level (e.g., risk of bias), and at review level (e.g., incomplete retrieval of identified research, reporting bias). *Comment on the validity of the assumptions, such as transitivity and consistency. Comment on any concerns regarding network geometry (e.g., avoidance of certain comparisons).*	24

Conclusions	26	Provide a general interpretation of the results in the context of other evidence, and implications for future research.	25
**FUNDING**			
Funding	27	Describe sources of funding for the systematic review and other support (e.g., supply of data); role of funders for the systematic review. This should also include information regarding whether funding has been received from manufacturers of treatments in the network and/or whether some of the authors are content experts with professional conflicts of interest that could affect use of treatments in the network.	26
